# New Exposure Biomarkers as Tools for Breast Cancer Epidemiology, Biomonitoring, and Prevention: A Systematic Approach Based on Animal Evidence

**DOI:** 10.1289/ehp.1307455

**Published:** 2014-05-12

**Authors:** Ruthann A. Rudel, Janet M. Ackerman, Kathleen R. Attfield, Julia Green Brody

**Affiliations:** 1 Silent Spring Institute, Newton, Massachusetts, USA; 2 Harvard School of Public Health, Harvard University, Boston, Massachusetts, USA

## Abstract

Background: Exposure to chemicals that cause rodent mammary gland tumors is common, but few studies have evaluated potential breast cancer risks of these chemicals in humans.

Objective: The goal of this review was to identify and bring together the needed tools to facilitate the measurement of biomarkers of exposure to potential breast carcinogens in breast cancer studies and biomonitoring.

Methods: We conducted a structured literature search to identify measurement methods for exposure biomarkers for 102 chemicals that cause rodent mammary tumors. To evaluate concordance, we compared human and animal evidence for agents identified as plausibly linked to breast cancer in major reviews. To facilitate future application of exposure biomarkers, we compiled information about relevant cohort studies.

Results: Exposure biomarkers have been developed for nearly three-quarters of these rodent mammary carcinogens. Analytical methods have been published for 73 of the chemicals. Some of the remaining chemicals could be measured using modified versions of existing methods for related chemicals. In humans, biomarkers of exposure have been measured for 62 chemicals, and for 45 in a nonoccupationally exposed population. The Centers for Disease Control and Prevention has measured 23 in the U.S. population. Seventy-five of the rodent mammary carcinogens fall into 17 groups, based on exposure potential, carcinogenicity, and structural similarity. Carcinogenicity in humans and rodents is generally consistent, although comparisons are limited because few agents have been studied in humans. We identified 44 cohort studies, with a total of > 3.5 million women enrolled, that have recorded breast cancer incidence and stored biological samples.

Conclusions: Exposure measurement methods and cohort study resources are available to expand biomonitoring and epidemiology related to breast cancer etiology and prevention.

Citation: Rudel RA, Ackerman JM, Attfield KR, Brody JG. 2014. New exposure biomarkers as tools for breast cancer epidemiology, biomonitoring, and prevention: a systematic approach based on animal evidence. Environ Health Perspect 122:881–895; http://dx.doi.org/10.1289/ehp.1307455

## Introduction

Breast cancer is the most common invasive malignancy among women in the United States, and the leading cause of death in women from their late 30s to early 50s (Brody et al. 2007b; Woloshin et al. 2008). The American Cancer Society (2010) estimated the global economic costs of premature death and disability from breast cancer at $88 billion/year. Incidence rates vary dramatically over time and geography, with breast cancer rates higher in recent generations and in more-developed countries. Treatment is arduous, debilitating, and expensive, costing $17 billion/year in the United States [Interagency Breast Cancer and Environment Research Coordinating Committee (IBCERCC) 2013]. Thus, the potential benefits of improving preventative efforts are large. Four authoritative panels have pointed to further study of environmental chemicals as a promising direction for prevention [Cogliano et al. 2011; IBCERCC 2013; Institute of Medicine (IOM) 2011; President’s Cancer Panel 2010].

The rationale for studying environmental chemicals and breast cancer draws, in part, on epidemiological findings for other, easier to study, exposures. Preventable risk factors for breast cancer include medical radiation, aspects of reproductive history, increased body weight after menopause, lack of physical exercise, alcohol consumption, combination hormone replacement therapy (HRT), combination hormonal contraceptives, prenatal diethylstilbestrol (DES) exposure, and probably tobacco smoke [Hoover et al. 2011; IBCERCC 2013; International Agency for Research on Cancer (IARC) 2012; IOM 2011]. Several of these risk factors represent chemical exposures, suggesting that exposure to chemicals with similar properties may also pose preventable risks. For example, alcohol shares properties with other solvents, and tobacco smoke is but one member of a large family of toxicologically similar combustion products, including vehicle exhaust and air pollution. Given that pharmaceutical hormones are linked to breast cancer, other hormonally active chemicals likely also affect risk.

In addition, toxicological studies show genotoxicity, hormonal activity, and increased mammary tumors in rodents after exposure to many chemicals used in industry and consumer products and found in air and water, indicating that these and other chemicals could affect breast cancer risk. We previously identified 216 chemicals that have been reported to increase mammary gland tumors in rodents (Rudel et al. 2007). Although the strength of the evidence linking these chemicals to breast cancer varies, most of them also show evidence of genotoxicity and tumors at other sites, strengthening the case that they may also be carcinogenic in humans (Rudel et al. 2007). Many researchers have concluded that rodent mammary gland development and carcinogenesis is generally a good model for humans, as discussed in the well-developed literature on the subject (Cardiff et al. 2002; Russo IH and Russo J 1996; Russo J and Russo IH 1993, 2004) and as reflected in the consensus statements from a recent workshop that included > 50 academic and government scientists, including 26 whose research focus is on mammary gland biology and toxicology (Rudel et al. 2011). The IOM, the IARC, the IBCERCC, and others recommend using toxicological data, such as the mammary carcinogen list (Rudel et al. 2007), to set priorities for further research and possible exposure reduction (Cogliano et al. 2011; IBCERCC 2013; IOM 2011).

To implement these recommendations, researchers need tools to track human exposure. Exposure biomarkers—chemicals or metabolites measured in biological media—are prime tools because they can approximate internal dose and identify highly exposed groups. Alternative exposure assessment methods are limited: Self-reports are rarely useful for environmental chemicals because people are often unaware of their exposures. Women’s work histories have not lent themselves to occupational assessments for breast cancer, although this is changing, and geographic location can be useful only in limited situations involving accidents or disasters or when environmental monitoring data are available. In addition to their use in epidemiological studies, exposure biomarkers are valuable for tracking exposure levels in the general population [e.g., via the National Health and Nutrition Examination Survey (NHANES) and in subgroups with unusual exposures or vulnerabilities] and for designing and assessing exposure reduction efforts.

Despite its potential power, the use of exposure biomarkers in breast cancer research has so far been limited to a few types of chemicals. This review was intended to expand epidemiology studying breast cancer and environment by bringing together needed tools. Our previous work used toxicological studies to identify priority chemicals for breast cancer studies based on biological plausibility (Rudel et al. 2007, 2011). The information we present here builds on that work by describing methods available for exposure assessments in epidemiological studies. Because reducing exposure to plausible breast carcinogens can help prevent breast cancer, we also highlight new priorities for biomonitoring programs to effectively monitor population exposure, identify highly exposed groups, and evaluate exposure reduction efforts.

To expand the use of exposure biomarkers relevant to breast cancer, we summarized biomonitoring measurement methods for chemicals that cause mammary gland tumors in animals. We focused on 102 chemicals to which large numbers of women are likely exposed. To inform the use of these biomarkers, we also summarized exposure levels in NHANES and in special populations and identified common exposure sources. We prioritized the chemicals and grouped them based on exposure, carcinogenic potential, and chemical structure. To facilitate discussion of the breast cancer relevance of rodent mammary gland carcinogens, we compared relevant human and animal evidence, and we discuss the strengths and limitations of this inference. Finally, we compiled a list of cohort studies with stored biological samples in which exposure biomarkers could readily be applied.

## Methods

*Chemical selection*. We previously identified 216 chemicals as potential breast carcinogens because they caused mammary gland tumors in rodent studies (Rudel et al. 2007), based on information from Gold et al. (2005), the National Toxicology Program (NTP 2005a, 2005b), *IARC Monographs* published in or before 2005 (Volumes 1–85; http://monographs.iarc.fr/ENG/Monographs/PDFs/), and the Chemical Carcinogenesis Research Information System (CCRIS; http://toxnet.nlm.nih.gov/newtoxnet/ccris.htm). We then identified 102 of these 216 as having broad exposure in the population because *a*) they are produced in high volumes (Rudel et al. 2007); *b*) > 5,000 women are occupationally exposed each year (Rudel et al. 2007); *c*) they are present in food, air pollution, or consumer products (Rudel et al. 2007); *d*) they are pharmaceuticals that have been prescribed to large numbers of women (Friedman et al. 2009); or *e*) they are pharmaceuticals often prescribed to pregnant women (Hoover et al. 2011).

*Systematic search and summary of exposure biomarkers*. We searched PubMed (http://www.ncbi.nlm.nih.gov/pubmed) to identify exposure biomarkers for the 102 selected chemicals. For each chemical, we searched for studies using a biomarker of exposure on occupationally or environmentally exposed populations or the general population, as well as recent (since 2000) studies of biomarker method development, which might use small numbers of human or animal samples. We excluded studies of metabolism and distribution, methods in environmental media (e.g., air, water, soil, dust), and biomarkers of early effect (e.g., oxidative stress, apoptosis) unless the effect was specific to that chemical. The search format was as follows: {(chemical name) OR [Chemical Abstracts Service number (CAS)]} AND (biomarker OR biological marker OR biological monitoring OR urine OR blood).

When the initial search returned > 400 results, we refined the search by adding keywords, depending on the nature of the irrelevant results. Additional keywords included “exposure” or “(chromatography OR spectrometry OR assay OR detection OR quantification OR quantitation)” or “(occupational OR population OR human).” If the initial search returned < 10 results, we also searched for ([chemical name] OR [CAS]) AND (chromatography OR spectrometry OR assay OR detection OR quantification OR quantitation). When the initial search returned no useful results, we also searched for “[chemical name] OR [CAS],” which in a few cases yielded relevant results that had not appeared in the initial search.

We reviewed NHANES reports, information on the Centers for Disease Control and Prevention (CDC) website (CDC 2014), and articles with information about NHANES results and methods in order to identify NHANES analytical methods currently used to measure exposure to the 102 mammary carcinogens of interest, as well as to identify methods that could easily be adapted to do so.

We then reviewed and summarized the abstracts, reports, and review articles retrieved by these searches. In preparing the summaries, we gave more careful attention to review articles (which we retrieved and read in their entirety), to more recent articles (since 2005, or since 2000 for chemicals that had fewer results), and to those that included information on analytical methods in the abstract. In a few cases, we included additional information from modified searches. The summaries followed a standard format that included the most sensitive method or methods found *a*) for each biological medium (primarily blood and urine); *b*) for the parent compound, metabolites, and adducts; and *c*) for general population and occupational settings. Although we searched for methods only for blood and urine, we included methods in other media (e.g., saliva, breast milk) if these appeared in the search results. We included quantification limits and concentrations reported in human populations.

Anticipated sources of exposure. We previously (Rudel et al. 2007) reported information on anticipated sources of exposure from Budavari et al. (1996), Department of Health and Human Services (DHHS 2004), Environmental Defense Fund (2004), Food and Drug Administration (2005), IARC (http://monographs.iarc.fr/ENG/Monographs/PDFs/), National Institute for Occupational Safety and Health 1990, NTP (2005a, 2005b), Pesticide Action Network (2000), U.S. Environmental Protection Agency (U.S. EPA 2005a, 2005b), and toxicological databases [CCRIS (Household Products Database; http://hpd.nlm.nih.gov/), HSDB (Hazardous Substances Data Bank; http://toxnet.nlm.nih.gov/newtoxnet/hsdb.htm), PubChem (http://pubchem.ncbi.nlm.nih.gov/), and Toxnet (Toxicology Data Network; http://toxnet.nlm.nih.gov/)]. In the present work, we added information from the Canadian Priority Substances List (Health Canada 2001), California Proposition 65 listings [California Office of Environmental Health Hazard Assessment (California OEHHA) 2014], the NTP *12th Report on Carcinogens* (NTP 2011), U.S. EPA Action Plans (U.S. EPA 2012), and European Chemicals Agency (ECHA) listings of substances of very high concern (ECHA 2013).

*U.S. population levels reported in the CDC exposure report*. Of the chemicals in our list, 23 are, have been, or will be included in NHANES (CDC 2009) as are a number of polycyclic aromatic hydrocarbons (PAHs) that may serve as reasonable proxies for exposure to the carcinogenic PAHs on our list. For these, we reviewed full papers and identified analytical methods for blood and urine, detection limits, and detection frequencies in NHANES analyses of the general population.

*Priorities for breast cancer–relevant epidemiology and biomonitoring*. We identified priority chemicals or chemical families based on exposure and carcinogenicity and then condensed the chemical list by combining chemicals with similar structures and measurement methods (e.g., nitro-PAHs, heterocyclic amines). We prioritized chemicals by whether we anticipated widespread exposure, there was suggestive evidence of breast cancer risk in epidemiological studies, or they were prioritized for attention by governmental agencies.

*Animal–human concordance for breast cancer*. To evaluate the strength of evidence supporting an inference that rodent mammary carcinogens are also likely to be human breast carcinogens, we compared human and animal evidence for agents identified as plausibly linked to breast cancer in major reviews. We based our assessments of human evidence on IARC assessments for nine agents (Cogliano et al. 2011; IARC 2012). For human evidence on heterocyclic amines and four organochlorines, we relied on other authoritative reviews (Brody et al. 2007b; Hoover et al. 2011; Michels et al. 2007), and for five nonhormonal pharmaceuticals, we relied on an observational study from Kaiser Permanente (Friedman et al. 2009). Animal study findings came from original research papers, NTP reports, and other government reports.

*Cohort studies*. We compiled a list of cohort studies that have collected biological samples and health data from women, so researchers can readily find opportunities to apply the exposure biomarkers prospectively. We identified studies by searching the National Institutes of Health (NIH) 2009 Computer Retrieval of Information on Scientific Projects (CRISP) (NIH 2009) and 2012 RePORTER (NIH 2012) databases with the terms “breast cancer cohort,” examining other online resources (Environmental Health Risk in European Birth Cohorts 2010; National Cancer Institute 2013, 2014), communicating with researchers studying women’s health, and examining articles listed in PubMed as “related” to those from studies previously identified. For each cohort, we collected the following information: institution(s), principal investigator(s), funder, study population, study period, exposure measurements, health outcomes, and study website. We verified the information with the study investigators or contact people identified on study websites.

## Results

We identified exposure-source information for all 102 of the rodent mammary carcinogens and exposure biomarker methods for 73. The CDC has measured or will soon measure biomarkers of exposure to 23 of these in the general U.S. population. We found 19 agents with evidence as human breast carcinogens that could be compared for concordance with animal studies. We identified 60 cohort studies, covering > 3.5 million women and girls, that have collected biological samples in which these biomarkers could be tested and evaluated in relation to breast cancer or pubertal development.

*Exposure biomarker methods*. We found exposure measurement methods for almost three-quarters of the 102 mammary carcinogens. Specifically, methods have been published for 73 of the chemicals, and biomarkers for 62 have been measured in humans, 45 of these in a nonoccupationally exposed population. Exposure to 23 (plus noncarcinogenic PAHs) has been or will soon be measured through validated methods in the NHANES study of the U.S. population (see Supplemental Material, Table S1). Some of the chemicals for which we did not find methods could be analyzed using existing methods for structurally related compounds.

Generally, the biomarker methods measure either the parent compound in blood or a metabolite—sometimes not specific to a single parent compound—in urine. DNA and protein adducts, consisting of the parent or metabolite bound to DNA or to a protein, are also widely used. In this review, metabolites are considered to be specific to the parent compound unless noted otherwise.

Measurements in the NHANES sample of the U.S. population (Table 1) show that some biomarkers are detected in most people, whereas others are rarely detected, although exposures may be more common in subpopulations. The CDC National Center for Environmental Health laboratory that performs these measurements often collaborates with epidemiologists to analyze biological samples in their studies.

**Table 1:** Rodent mammary gland carcinogens included in NHANES exposure surveillance.

Parent chemical	Blood biomarker	Urine biomarker	Detection frequency (%)	Reference
1,1-Dichloroethane	1,1-Dichloroethane	None	< 5	CDC 2009
1,2-Dibromo-3-chloropropane	1,2-Dibromo-3-chloropropane	None	< 5	CDC 2009
1,2-Dibromoethane	None	HEMA (nonspecific)^*a*^	71 (nonspecific metabolite)^*a*^	Alwis et al. 2012; Calafat et al. 1999
1,2-Dichloroethane	1,2-Dichloroethane	None	< 5	CDC 2009
1,2-Dichloropropane	1,2-Dichloropropane	None	< 5	CDC 2009
1,3-Butadiene	None	MA metabolites	NA	Alwis et al. 2012
Acrylamide	Hb adducts of acrylamide and glycidamide	MA metabolites	> 50 (blood)	Alwis et al. 2012
Acrylonitrile	None	HEMA (nonspecific)^*a*^; specific MA metabolite	71 (nonspecific metabolite)^*a*^	Alwis et al. 2012; Calafat et al. 1999
Atrazine	None	Atrazine mercapturate	< 5	CDC 2009
Benzene	Benzene	MA metabolites	> 50 (blood)	Alwis et al. 2012; CDC 2009
Carbon tetrachloride	Carbon tetrachloride	None	< 5, 2003–2004 (> 25, 2001–2002)	CDC 2009
Chlordane	Serum: oxychlordane, *trans*-nonachlor	None	> 50	CDC 2009
Dichlorvos	None	Dimethyl phosphate (nonspecific)^*b*^	> 25 (nonspecific metabolite)^*b*^	CDC 2009
Ethylene oxide	Hb adducts	HEMA (nonspecific)^*a*^	71 (nonspecific urinary metabolite)^*a*^	Alwis et al. 2012; Calafat et al. 1999; CDC 2008
Propylene oxide	Hb adducts	MA metabolites	NA	Alwis et al. 2012; CDC 2008
Fenvalerate	None	Phenoxybenzoic acid (nonspecific)^*c*^	75 (nonspecific metabolite)^*c*^	CDC 2009; Riederer et al. 2008
Methylene chloride	Methylene chloride	None	< 5	CDC 2009
Methyleugenol	Serum: methyleugenol	None	98	Barr et al. 2000
Nitrobenzene	Nitrobenzene	None	< 5	CDC 2009
PAHs^*d*^	None	22 metabolites (e.g., 1-hydroxypyrene)	98	CDC 2012; Li et al. 2008
PFOA	Serum: PFOA	None	> 50	CDC 2012
Styrene	Styrene	MA metabolite, mandelic acid	> 25 (blood)	Alwis et al. 2012; CDC 2009
Vinyl chloride	None	HEMA (nonspecific)^*a*^	71 (nonspecific metabolite)^*a*^	Alwis et al. 2012; Calafat et al. 1999
Vinylidene chloride (1,1-dichloroethene)	Vinylidene chloride	None	< 5	CDC 2009
Abbreviations: Hb, hemoglobin; HEMA, 2-hydroxyethyl mercapturic acid; MA, muconic acid; NA, not applicable; PFOA, perfluorooctanoic acid. ^***a***^A metabolite of vinyl chloride, ethylene oxide, 1,2-dibromoethane, acrylonitrile, and other electrophilic two-carbon compounds (Alwis et al. 2012; Calafat et al. 1999). ­^***b***^A metabolite of > 15 organophosphate insecticides, including dichlorvos, malathion, and methyl parathion (CDC 2009). ^***c***^A metabolite of ≥ 6 pyrethroid insecticides (CDC 2009). ^***d***^PAH metabolites have not been identified as rodent mammary carcinogens in this method, but exposure is likely to be correlated with carcinogenic PAHs or nitro-PAHs, in some cases.				

Supplemental Material, Table S1, summarizes known sources of exposure and methods for biomonitoring for each of the 102 rodent mammary gland carcinogens. These 102 carcinogens comprise a diverse set of chemicals and exposures, including components of automobile exhaust, gasoline, and air pollution (1,3-butadiene, benzene, PAHs, nitro-PAHs), chemicals in food and drinking water [acrylamide, ochratoxin A, heterocyclic amines, styrene, 3-chloro-4-(dichloromethyl)-5-hydroxy-2(5H)-furanone (MX)], chemicals in consumer products and building materials (flame retardants, aromatic amines, perfluorinated compounds), pharmaceuticals, and endocrine disruptors as well as some chemicals with important occupational exposures [halogenated solvents, ethylene oxide (EtO)]. From this list, we identified higher-priority chemicals or chemical families based on exposure and carcinogenicity, as indicated below, resulting in 17 chemicals or groups of related chemicals. We prioritized chemicals/chemical groups based on stronger, more consistent mammary tumor effects in animal studies, greater exposure potential, and availability of methods. Exposure sources and measurement methods for these 17 chemicals and chemical groups are summarized in Table 2, and each is discussed briefly below. The groups may contain some individual chemicals that are not high priority on their own but which can be measured by the same method as a prioritized chemical. Conversely, the 27 chemicals not part of the 17 groups are still of high interest for breast cancer studies, and some of them can be measured with—or could be incorporated into—existing methods. Chemical structures for all the chemicals along with additional information on regulation, governmental assessments, exposure, and health effects are collected in the Silent Spring Institute Mammary Carcinogens Review Database (http://sciencereview.silentspring.org/mamm_about.cfm).

**Table 2:** Priority chemicals for breast cancer–relevant epidemiology and biomonitoring.

Chemical	Common exposure sources	Biomarkers
1,3-Butadiene	Gasoline, vehicle exhaust, tobacco smoke, heating of some cooking oils	DNA and hemoglobin adducts in blood, derived from epoxide metabolites; mercapturic acid metabolites in urine
Acrylamide	Cooked food, tobacco smoke, water-treatment by-products, some consumer products	Hemoglobin adducts of acrylamide and glycidamide in blood; urinary mercapturic acid metabolites of acrylamide and glycidamide
Aromatic amines I: TDA and TDIs	Uncured or newly finished polyurethane foam, spray-in insulation, sealants and coatings, some breast implants	TDA and hemoglobin adducts in blood, TDA in urine (Most studies have tested occupationally exposed populations, but many find TDA in “unexposed” controls)
Aromatic amines II: benzidine and aniline dyes, combustion products, other	Hair and textile dyes; used in the production of paints, printing inks, liquid crystal displays, and inkjet and laser printers, and in the food industry	Parent compound in blood or urine; DNA and hemoglobin adducts in blood or breast milk
Benzene	Gasoline, vehicle exhaust, tobacco smoke, solvents	DNA and protein adducts in blood and dried blood spots; urinary metabolites sPMA (specific to benzene) and ttMA (metabolite of benzene and the common food preservative sorbate)
Halogenated organic solvents (e.g., methylene chloride)	Dry cleaning, spot remover, glues, degreasers, paint strippers, aerosol propellants, contaminated drinking water (Use is decreasing over time)	Parent compound in whole blood and urine Infrequently detected in blood from general population but widespread occupational exposure has been documented; parent compounds have been detected in urine from occupationally exposed populations, and methylene chloride has been detected in urine from general population
Ethylene oxide, propylene oxide	Tobacco smoke, food and medical sterilization, vehicle exhaust, paint	DNA and hemoglobin adducts in blood; mercapturic acid metabolites in urine
Flame retardants and degradation products [2,2-bis(bromomethyl)-1,3-propanediol, 2,3-dibromo-1-propanol]	Flame retardants; primarily used in plastics and foams	Parent compound or metabolite in urine
Heterocyclic amines	Grilled meat	Parent compound, protein adducts, and DNA adducts in blood; parent compound in urine and hair
Hormones and endocrine disruptors (e.g., endogenous and exogenous estrogens and estrogen mimics)	Pharmaceutical hormones, consumer products, and commercial chemicals with hormonal activity	Clinical and research methods are available to measure endogenous hormone levels in blood and urine; the MCF-7 cell proliferation assay has been used to measure estrogenic activity in extracts of adipose tissue from breast cancer cases and controls; development of methods to conduct this assay in blood, and to distinguish endogenous and exogenous estrogen signals, would allow integrated assessments of exposure to xenoestrogens
MX	Water disinfection	Urinary trihaloacetic acids are used as exposure biomarkers for chlorinated drinking water, but improved exposure biomarkers are needed for MX and other highly genotoxic disinfection by-products
Nitro-PAHs (e.g., 1-nitropyrene)	Diesel exhaust	Hemoglobin adducts in blood, metabolites in urine
Ochratoxin A	Mycotoxin in grains, nuts, pork; also present in moldy environments	Ochratoxin A and its metabolites in blood, urine, breast milk
PAHs (e.g., BaP)	Vehicle exhaust, tobacco smoke, charred food	Protein adducts and DNA adducts in blood; oxidized metabolites in urine; parent compounds measured in hair, breast milk (Improved exposure biomarkers are needed)
PFOA, related compounds	Grease-, water-, and stainproof coatings; contaminated drinking water	Parent compound in blood and breast milk
Pharmaceuticals (non­hormonal)	A number of over-the-counter, veterinary, and prescription medicines that induce mammary tumors	Few exposure biomarkers have been developed for use in the general population, but in many cases LC-MS/MS methods have been reported for the parent compound in plasma or metabolites in urine; in some cases exposure can be ascertained from self-report or medical records
Styrene	Building materials and consumer products made from polystyrene; indoor air, cigarette smoke, polystyrene food packaging	Parent compound in whole blood; urinary mercapturic and mandelic acid metabolites
Abbreviations: BaP, benzo[*a*]pyrene; LC-MS/MS, liquid chromatography–tandem mass spectrometry; PFOA, perfluorooctanoic acid; sPMA, *S*-phenylmercapturic acid; TDA, 2,4-toluene diamine; TDI, toluene diisocyanates; ttMA, *trans*, *trans*-muconic acid. For more information, including a list of chemicals in each group, see Supplemental Material, Table S1.		

1,3-Butadiene. 1,3-Butadiene exposure in the general population is primarily via inhalation of cigarette smoke, automobile exhaust, and gasoline fumes as well as via emissions from industrial facilities. Occupational exposure occurs in many industries, especially synthetic rubber manufacturing and petroleum refining, with > 12 billion pounds per year produced globally (Occupational Safety and Health Administration 2012). Many occupational studies, epidemiological studies, and studies comparing smokers and nonsmokers have used biomarkers of exposure to 1,3-butadiene in blood and urine. Common approaches include measuring DNA and hemoglobin adducts in blood (Il’yasova et al. 2009; Ogawa et al. 2006; Swenberg et al. 2011) and measuring mercapturic acid metabolites in urine (Alwis et al. 2012). The CDC is planning to measure the urinary mercapturic acid metabolites in future NHANES reports (Alwis et al. 2012). DNA and hemoglobin adducts are likely to represent cumulative exposure over several weeks or longer, whereas urinary metabolite levels reflect more recent exposures (Boogaard 2002; Carmella et al. 2009).

Acrylamide. Major sources of acrylamide exposure include diet (especially starchy foods cooked at high temperatures such as french fries) and tobacco smoke. Acrylamide exposures are believed to be low from other sources such as grouts, adhesives, and polyacrylamide gels used in many consumer products (e.g., diapers) and in drinking-water treatment. The CDC has measured the hemoglobin adducts of acrylamide and its metabolite glycidamide (CDC 2009) and plans to measure urinary mercapturic acid metabolites of acrylamide and glycidamide in NHANES participants (Alwis et al. 2012).

Aromatic amines. Aromatic amines contain nitrogen bound to benzene or other aromatic rings. They are important intermediates in the industrial synthesis of polyurethane, pesticides, dyes, and many other products. We identified 15 aromatic amines that produce mammary gland tumors in rodent cancer bioassays (see Supplemental Material, Table S1). Many other commercially important aromatic amines have not been tested for carcinogenicity in rodents. The 15 that caused rodent mammary tumors comprise 2,4-toluene diamine (TDA) and toluene diisocyanate (TDI), and 13 associated with azo dyes and combustion sources. Methods for biomonitoring them typically involve measuring the parent compound in blood or urine, or DNA or hemoglobin adducts of the parent compound (see Supplemental Material, Table S1).

TDA is an aromatic amine metabolite of TDI, which is used in the production of polyurethane foams and sealants. The U.S. EPA recently created an action plan for TDI because of the potential for exposure from spray-in foam insulation and sealants containing TDI (U.S. EPA 2011b). In addition to concerns about cancer, TDI exposure has been shown to cause asthma and lung damage (U.S. EPA 2011b). Exposures to trinitrotoluene and dinitrotoluene, used in explosives and munitions, also produce elevated levels of TDA in urine and blood (ECHA 2013). Exposure to TDA is usually assessed by measuring the chemical in urine or blood samples, and some studies have measured hemoglobin adducts of TDA (Jones et al. 2005). A study of women with polyurethane breast implants detected TDA in the urine of about half the study participants (Hester et al. 1997).

Azo dyes are derivatives of aromatic amines such as benzidine and aniline. Most of these are no longer used or produced in the United States, Europe, or Japan—in part because they are known to cause bladder cancer in humans (NTP 2011). However, many azo dyes are still produced and used in significant quantities, mostly in Asia (U.S. EPA 2010), in the production of textiles, hair dyes, paints, printing inks, paper, and pharmaceuticals; as reagents and biological stains in laboratories; in the food industries; and in liquid crystal displays, laser and inkjet printers, and electro-optical devices (U.S. EPA 2010). One study reported DNA–aminobiphenyl adducts in epithelial-cell DNA isolated from human breast milk in women who had used hair-coloring products (Ambrosone et al. 2007).

Some aromatic amines (e.g., 4-aminobiphenyl) are present in tobacco smoke and other combustion products (NTP 2011).

Benzene. The highest exposures to benzene are from gasoline (from, e.g., riding in a car, pumping gasoline, storing gasoline in a basement or attached garage) and tobacco smoke, although automobile exhaust and other forms of urban and industrial air pollution are also important exposure sources (Dodson et al. 2007; NTP 2011). Benzene is also used in some consumer products, including adhesive removers, paints, sealants, finishers, and engine fuel and oils (DHHS 2004). The CDC and others have measured benzene in blood samples taken from the general population, generally detecting it in all or most samples; however, this method requires a relatively large (3–10 mL) whole blood sample and only reflects recent exposure (Blount et al. 2006). Others have measured benzene in urine and breath, benzene metabolites in urine, and adducts to proteins and DNA in blood and dried blood spots (see Supplemental Material, Table S1). At present, the CDC plans to perform benzene biomonitoring in NHANES by measuring urinary mercapturic acid metabolites (Alwis et al. 2012).

Halogenated organic solvents. We identified methylene chloride and nine other organic solvents as causing mammary gland tumors in animal bioassays. These chemicals were widely used in the past, with uses including dry cleaning, hair-spray propellant, soil fumigants, food processing, gasoline additives, and paint and spot removers. Although their use has declined, occupational exposures are still common, and some consumer exposure remains. In past years, the CDC has measured blood levels of some of these chemicals in NHANES, but the method requires a relatively large (3–10 mL) whole blood sample, only reflects recent exposure, and is vulnerable to contamination by laboratory solvents (Blount et al. 2006) (see Supplemental Material, Table S1). Methods may be developed to measure parent compounds or metabolites of halogenated organic solvents in urine, but these will likely still reflect only recent exposures and be vulnerable to contamination. The CDC plans to measure urinary mercapturic acid metabolites of many volatile organic compounds (VOCs) in NHANES samples, and possibly some of these halogenated VOCs could be integrated into this method (Alwis et al. 2012) although the only metabolite of a halogenated mammary carcinogen to be measured by this method is 2-hydroxyethyl mercapturic acid (HEMA), a common metabolite of 1,2-dibromoethane, vinyl chloride, acrylonitrile, and EtO (Calafat et al. 1999; CDC 2009). Other potential nonspecific biomarkers include haloacetic acids and haloalcohols in urine.

EtO and 1,2-propylene oxide (PO). EtO is a gas used to sterilize medical equipment, food and spices, clothing, and musical instruments (NTP 2011). It has also been detected in tobacco smoke and auto exhaust (NTP 2011). EtO is a high-production-volume chemical used to manufacture many other chemicals such as ethylene glycol; thus, one exposure source might be air pollution near industrial facilities (NTP 2011). Occupational exposure has been widespread, including in health care settings, but exposure levels have been decreasing with time (NTP 2011). The CDC has measured the nonspecific urinary metabolite HEMA (Calafat et al. 1999; CDC 2009) and plans to add EtO-hemoglobin adducts to the current method that measures acrylamide- and glycidamide-derived hemoglobin adducts in NHANES blood samples (CDC 2008). DNA adducts of EtO have been measured in occupational and general population studies (e.g., Czene et al. 2002; Schettgen et al. 2010). Urine measurements reflect recent exposures, whereas DNA or protein adducts reflect cumulative exposure over weeks or months.

PO, another high-production-volume industrial chemical, is used to manufacture other chemicals (including polyurethane foam) and as a sterilant and fumigant and is used in some automotive and paint products (NTP 2011). Tobacco smoke is also a source of exposure (IARC 2010). The CDC plans to measure a PO-derived mercapturic acid in the urine of NHANES participants (Alwis et al. 2012). Hemoglobin and DNA adducts have also been used to measure exposures in some studies, and the CDC method for measuring acrylamide-derived hemoglobin adducts in blood was first developed to measure PO exposure (CDC 2008).

Flame retardants and metabolites. We identified a flame retardant and a flame retardant metabolite, with similar chemical structures, that are genotoxic and also potent multisite carcinogens, including in the mammary gland. 2,2-Bis(bromomethyl)-1,3-propanediol is a high-production-volume flame retardant used in polyester resins, plastic polymers, and rigid polyurethane foams (NTP 2011). It is expected to be very persistent in water; however, it is rapidly glucuronidated and excreted by rats, and less rapidly excreted by humans (Hoehle et al. 2009; Kong et al. 2011; Rad et al. 2010). The second chemical of interest is 2,3-dibromo-1-propanol, which is an impurity, degradation product, and metabolite of two flame retardants: tris(2,3-dibromopropyl) phosphate, formerly used in children’s pajamas before this use was restricted in 1977, and tetrabromobisphenol A bis (2,3-dibromopropyl ether), a high-production-volume chemical currently used in plastics (NTP 2002). Several flame retardants with similar or identical structures except for the substitution of chlorine for bromine atoms [e.g., tris (dichloropropyl) phosphate, also known as chlorinated tris] also seem likely to increase breast cancer risk. Although these chlorinated tris alkyl phosphates are known carcinogens (California OEHHA 2014), to our knowledge rodent studies have not reported mammary tumors.

Biomarkers for these halogenated tris-alkylphosphate flame retardants and their degradation products are not well developed. 2,3-Dibromo-1-propanol has been measured in urine by gas chromatography–mass spectrometry (GC-MS) (Blum et al. 1978; De Alwis et al. 2007) and bis(1,3-dichloro-2-propyl) phosphate, a metabolite of chlorinated tris, has been analyzed in urine by liquid chromatography–tandem mass spectrometry (LC-MS/MS) (Cooper et al. 2011). Knowledge gaps include pharmacokinetic studies to identify suitable metabolites for biomonitoring and methods development, including synthesis of analytical standards.

Heterocyclic amines. Both meat cooked at high temperatures and tobacco smoke contain heterocyclic amines (NTP 2011), four of which have been associated with mammary gland tumors in rodents. Although biomarkers of exposure have been developed and used in epidemiological studies of cancer risk, including for breast cancer, most epidemiological studies have used self-reported dietary intake and smoking as exposure measures. Common exposure biomarkers are protein or DNA adducts in blood or tissues, the parent compound in blood or hair, and the parent compound or metabolites in urine (see Supplemental Material, Table S1).

Endogenous and pharmaceutical hormones and other endocrine-disrupting chemicals (EDCs). Estrogens, progesterone, and DES—along with other hormones—cause mammary gland tumors in rodents, and a few weaker EDCs have shown modest mammary tumor effects in rodent bioassays (e.g., amsonic acid, which is used in the manufacture of fluorescent whitening agents) (Rudel et al. 2007). EDCs may also affect breast cancer risk via pathways that would not be evident in rodent cancer screens, for example, by altering mammary gland development (Rudel et al. 2011). Clinical laboratories commonly measure estradiol and other steroid hormone levels in blood by immunoassay, although such methods are imprecise and prone to interference from other hormones and hormone-binding proteins in serum or plasma (Blair 2010; Cao et al. 2004; Rosner et al. 2013). GC-MS and LC-MS/MS methods in blood and urine are more precise, sensitive, and specific (Blair 2010; Rosner et al. 2013; Stanczyk and Clarke 2010).

Chemicals known to be EDCs can be individually quantified. In addition, theoretically, functional tests can give estimates of total exposure to estrogenic activity or other forms of endocrine disruption; however, these methods are not yet well developed for use in biological samples. The MCF-7 cell proliferation assay has been used to measure estrogenic activity in extracts of adipose tissue in breast cancer cases and controls (Fernandez et al. 2007). The development of methods to conduct this assay in blood, and to distinguish endogenous and exogenous estrogen signals, would allow integrated assessments of exposure to xenoestrogens. In addition, functional assays in biological samples to measure EDC activity affecting nonestrogenic pathways would allow epidemiologists to study the impact of other hormonal mechanisms on breast carcinogenesis.

MX. MX is one of hundreds of genotoxic by-products of drinking-water disinfection. Although MX concentrations are typically much lower than those of regulated disinfection by-products, MX is more potently genotoxic and carcinogenic (McDonald and Komulainen 2005; Richardson et al. 2007). Exposure biomarkers are needed for MX and related disinfection by-products (Savitz 2012). A number of epidemiological studies of drinking-water disinfection exposures use urinary trihaloacetic acids, which are metabolites of other common disinfection by-products, as exposure biomarkers (Savitz 2012; Weisel et al. 1999). It is not known whether they are good proxies for the mutagenic disinfection by-products.

Nitro-PAHs. 1-Nitropyrene and other nitroPAHs are air pollutants thought to primarily come from diesel exhaust. Few studies have characterized general-population exposure levels, but biomarkers of exposure include urinary metabolites (e.g., 1-aminopyrene) (Huyck et al. 2010; Laumbach et al. 2009; Toriba et al. 2007) and hemoglobin adducts in blood (Zwirner-Baier and Neumann 1999). Six of these compounds—1,3-dinitropyrene, 1,8-dinitropyrene, 1-nitropyrene, 2-nitrofluorene, 4-nitropyrene, 6-nitrochrysene—are especially potent rodent mammary gland carcinogens (Rudel et al. 2007), making the development of better exposure biomarkers a high priority.

Ochratoxin A. Human exposure to the naturally occurring mycotoxin ochratoxin A occurs mainly through the consumption of contaminated grain, nuts, and pork products (IARC 1993; NTP 2011). Ochratoxin contamination of crops is more prevalent in some regions (e.g., the Balkan countries). Exposure has also been reported in individuals in the United States exposed to mold-contaminated environments (Hooper et al. 2009). Exposure biomarkers include measurement of the parent compound in blood, urine, breast milk, and other tissues using immunoassay methods with fluorescence detection as well as LC-MS/MS approaches (Hooper et al. 2009; Scott 2005). In a pilot study, Muñoz et al. (2009) suggested that levels of metabolites may be much higher than those of the parent compound in blood and urine and thus may be better exposure biomarkers.

PAHs. Exposure to PAHs, such as benzo[*a*]pyrene (BaP), occurs primarily through inhalation of tobacco smoke or polluted air and the ingestion of charred foods (NTP 2011). Of the five PAHs shown to cause mammary gland tumors in rodents, two—3-methylcholanthrene and 7,12-dimethylbenz[*a*]anthracene—are primarily used in research laboratories and three—BaP, dibenz[*a,h*]anthracene, and dibenzo[*def,p*]chrysene—are commonly measured products of combustion (see Supplemental Material, Table S1). However, air pollution and other combustion products are complex mixtures of many PAHs, most of which have not been evaluated for carcinogenicity. The most commonly used biomarkers of PAH exposure are DNA and protein adducts measured in blood; however, it is also possible to measure parent PAHs in blood and their hydroxy metabolites in urine. Some studies have used DNA or protein adducts that are specific to BaP (e.g., BaP diol epoxide–DNA or protein adducts), whereas others use nonspecific “bulky DNA adducts” or “BaP-like adducts” detected by 32-P–post labeling and immunoassays (Boysen and Hecht 2003; Käfferlein et al. 2010). Additional methods development is needed because researchers have had limited success differentiating between exposed and unexposed populations through the use of BaP-specific adducts, and it is unclear how measurements of PAH adducts or specific PAH concentrations relate either to specific exposures or to the overall carcinogenic potency of the complex mixture of PAHs in the environment (Shantakumar et al. 2005).

Perfluorooctanoic acid (PFOA). PFOA and other perfluorinated compounds (PFCs) are used in nonstick and stain-resistant coatings on rugs, furniture, clothes, and cookware as well as in fire-fighting applications, cosmetics, lubricants, paints, and adhesives. They are widely detected in blood samples in the United States (Lau et al. 2007). Despite the many consumer uses of this extensive and complex class of surfactant chemicals, the most important sources of exposure and health effects are not well defined. Exposure via use of consumer products is likely important, and in addition these compounds are common drinking-water and food contaminants (D’Hollander et al. 2010). PFOA has shown weak evidence of mammary tumors in rodents and has also been shown to disrupt estrogen, thyroid, and peroxisome proliferator–activated receptor (PPAR)–mediated hormonal signaling, as well as to alter mammary gland development (Lau et al. 2007; White et al. 2011). PFCs are persistent and are readily measured in blood in the general U.S. population as well as in groups exposed occupationally or through industrial contamination. NHANES has measured several PFCs, including PFOA, and reported detectable levels in the majority of the general population (Alwis et al. 2012).

Nonhormonal pharmaceuticals. A number of pharmaceuticals that are not prescribed as hormonally active drugs have been shown to cause rodent mammary tumors (Rudel et al. 2007). These include four chemotherapeutic agents, two veterinary drugs possibly present in food (2-amino-5-nitrothiazole and nitrofurantoin), the diuretic furosemide, the antifungal griseofulvin, several antiinfective agents, and two drugs that are no longer widely used (phenacetin, an over-the-counter pain reliever and the antihypertensive drug reserpine) (Rudel et al. 2007). Few exposure biomarkers can detect low-level exposures in the general population, but many studies have used LC-MS/MS to measure parent compounds in plasma, or metabolites in urine, to describe pharmacokinetics, monitor patients, and, in the case of at least one chemotherapeutic agent, monitor exposure of health care workers. In some cases, exposure can be ascertained from self-report or medical records (see Supplemental Material, Table S1).

Styrene. Exposure to styrene in the general population occurs at levels of micrograms per day due mainly to inhalation of indoor air and cigarette smoke and intake of food that has been in contact with polystyrene. Styrene is present in consumer products and building materials, including polystyrene, carpets, adhesives, hobby and craft supplies, and home maintenance products (IARC 2002; NTP 2011). NHANES and others have measured styrene and its metabolites and adducts in blood samples from the general population (Blount et al. 2006). Urinary mercapturic and mandelic acid metabolites will be included in future NHANES assessments (Alwis et al. 2012). A few studies have measured styrene in human saliva and breast milk (Blount et al. 2010; Sanchez et al. 2012).

Other chemicals. Twenty-seven additional high-exposure mammary gland carcinogens do not fall into the 17 priority categories described above but are still priorities for exposure and epidemiological studies (see Supplemental Material, Table S1). Exposure sources and biomarker methods for these are summarized in Supplemental Material, Table S1, along with methods for the chemicals in the groups described above. The 27 additional chemicals (see Supplemental Material, Table S1) include some pesticides, some chemicals that may be present in consumer products (e.g., acrylonitrile, nitrobenzene, dyes), and some food-related chemicals. For example, urethane is an industrial chemical but also a product of fermentation, methyl eugenol is a natural and artificial flavoring, and nitrosamines have been reported in some smoked meats but can also have industrial uses. Pesticides include captafol, clonitralid, 1,2-dibromochloropropane, dichlorvos, fenvalerate, malachite green, and sulfallate. Pesticides were not included as a group in Table 2 because they are not chemically similar and therefore require different methods for exposure measurement. About half of these pesticides have had some type of exposure biomarker method reported in the literature (e.g., Coronado et al. 2011; Riederer et al. 2008).

*Animal–human concordance*. Results of human breast cancer studies are generally consistent with rodent bioassays, although few agents have been studied in humans. There is consistent evidence in humans and animals for associations between breast cancer risk and hormonal pharmaceuticals, ionizing radiation, light at night/shift work, alcoholic beverages, EtO, heterocyclic amines/grilled meat, PAHs/tobacco smoke, and common industrial solvents (Brody et al. 2007a; Cogliano et al. 2011; Michels et al. 2007; Rudel et al. 2007). A single study by Kaiser Permanente evaluated breast cancer among women prescribed five nonhormonal pharmaceuticals that have been shown to cause rodent mammary tumors and found small but significantly increased risk for three of these, but no increased risk for two others (Friedman et al. 2009).

Table 3 also shows that animal and human evidence is consistently negative for some agents that do not induce mammary tumors in standard cancer bioassays. Studies of persistent organochlorine chemicals like dichlorodiphenyltrichloroethane (DDT), polychlorinated biphenyls (PCBs), and dioxin—which act as EDCs rather than as classical carcinogens—also show consistent findings between animal and human studies. These chemicals do not increase mammary gland tumors in standard cancer bioassays, which expose adult animals; similarly, human studies have generally not found significant relationships between adult serum concentrations and breast cancer risk. However, these EDCs may increase risk with early-life exposure by altering mammary gland development or hormone responsiveness, and there is limited evidence for this from both human and animal studies (Brody and Rudel 2008; Cohn et al. 2007; Rudel et al. 2011). Studies of genetically homogeneous animals have not evaluated the genetic differences in susceptibility that were suggested by human studies on PCBs and breast cancer (Brody et al. 2007b).

**Table 3:** Comparison of human and animal evidence for agents identified as plausibly linked to breast cancer in major reviews.^*a*^

Agent	Human breast	Rodent mammary	Reference (human)	Reference (rodent)
Estrogenic pharmaceuticals
HRT (estrogen–progestin)	Positive	Positive	Cogliano et al. 2011	Rudel et al. 2007
HRT (estrogen only)	Limited positive^*b*^	Positive	Cogliano et al. 2011	Rudel et al. 2007
Estrogen–progestin contraceptives	Positive	Positive	Cogliano et al. 2011	Rudel et al. 2007
DES (mother and daughter)	Positive	Positive	Cogliano et al. 2011; Hoover et al. 2011	Rudel et al. 2007
Other pharmaceuticals
Griseofulvin, furosamide, metronidazole	Limited positive^*b*^	Positive	Friedman et al. 2009	Rudel et al. 2007
Indomethacin, nitrofurantoin	Limited null finding^*c*^	Positive	Friedman et al. 2009	Rudel et al. 2007
Miscellaneous
Ionizing radiation	Positive	Positive	Cogliano et al. 2011	Rudel et al. 2007
Ethanol/drinking alcoholic beverages	Positive	Limited positive^*b*^	Cogliano et al. 2011	Oyesanmi et al. 2010
Heterocyclic amines in grilled/fried meat	Limited positive^*b*^	Positive	Michels et al. 2007	Rudel et al. 2007
Light at night/shift work/circadian	Limited positive^*b*^	Positive	Cogliano et al. 2011	Stevens 2009
EtO	Limited positive^*b*^	Positive	Cogliano et al. 2011	Rudel et al. 2007
PAHs (auto exhaust, cigarette smoke)	Limited positive^*b*^	Positive	Cogliano et al. 2011	Rudel et al. 2007
Common industrial solvents	Limited positive^*b*^	Positive	Brody et al. 2007a	Rudel et al. 2007
Persistent organochlorines
DDE [(DDT metabolite) in older adult blood)	Null	Null	Brody et al. 2007a; Snedeker 2001	NTP 1978
DDT (in blood at young age)	Limited positive^*b*^	No study	Brody et al. 2007a;^.^Cohn et al. 2007	—
PCBs (in older adult blood)	Null	Null	Brody et al. 2007a	NTP 2011
PCBs (in subpopulations with polymorphism)	Limited positive^*b*^	No relevant model	Brody et al. 2007a	—
TCDD/dioxin (in early life)	Limited positive^*b*^	Positive (with carcinogen challenge)	Brody et al. 2007a; Warner et al. 2011	Brown et al. 1998
Abbreviations: DDE, dichlorodiphenyldichloroethylene; TCDD, 2,3,7,8 tetrachlorodibenzo-*p*-dioxin. ^***a***^Agents with strongest human breast cancer evidence from reviews by IARC (2012b), Cogliano et al. (2011), Brody et al. (2007a), Hoover et al. (2011), Michels et al. (2007) and Friedman et al. (2009). Animal study findings from original research papers, NTP reports, and other government reports. ^***b***^Following Cogliano et al. (2011), “limited positive” indicates evidence sufficient to establish a credible causal relationship but not sufficient to rule out chance, bias, or confounding. ^***c***^A single study (Friedman et al. 2009) found no association between these two pharmaceuticals and breast cancer.				

*Breast cancer cohorts with archived biological samples*. We identified opportunities to apply novel exposure measures in breast cancer epidemiology in the form of 60 cohort studies and tissue banks that have collected biological samples from female participants years before ascertaining breast cancer incidence, breast cancer recurrence, or pubertal timing (see Supplemental Material, Tables S2 and S3).

The majority of these [42 studies, with a total of about 3.5 million enrolled women (see Supplemental Material, Table S2)] have ascertained or will ascertain breast cancer incidence as an outcome measure; one of these 42 studies also collected information on pubertal outcomes for the women’s daughters. An additional three studies, with a total of approximately 5,500 women enrolled, collected samples from women after a primary breast cancer diagnosis and are prospectively studying survival, recurrence, and contralateral primary breast cancer. A few of these studies have already included measurements of environmental chemicals (most commonly organochlorine pesticides) in blood, although many have so far used biological samples primarily to assess hormone levels and polymorphisms. The study populations, despite being mostly North American and western European, represent a wide variety of ethnic and demographic groups.

In addition, we identified 15 more cohort studies, with > 70,000 enrolled girls and young women (see Supplemental Material, Table S3), that collected biological samples and measured or will measure age at menarche, breast development, hormones, and other indicators of pubertal timing and reproductive health. Many of these studies have collected samples from their participants during infancy and from the girls’ mothers during pregnancy. Two of these have information on menopausal or other maternal outcomes. Assessing biomarkers of exposure to EDCs and other chemicals in these studies could provide valuable information about links between these chemicals and changes in breast development and pubertal timing. Given the importance of pubertal development to later-life breast health, and the well-established connection between early menarche and breast cancer risk, information about these end points could provide insights into the mechanisms by which early-life environmental exposures alter breast cancer risk.

Supplemental Material, Table S2, also includes 10 studies that, to our knowledge, have not collected biological samples from their participants but nonetheless provide opportunities for prospectively studying environmental exposures and breast cancer.

## Discussion

We compiled biomonitoring methods for 102 chemicals that are high priorities for breast cancer research and prevention efforts. We systematically identified these chemicals based on evidence that they cause mammary tumors in rodent bioassays and on production and use information that suggests current or historical exposure to the general population. We condensed the priority list into 17 chemical groups for immediate attention. Il’yasova et al. (2009) recently published a similar approach for expanding the scope of brain tumor epidemiology by prioritizing animal neurocarcinogens.

Our results show ample opportunity to expand breast cancer epidemiology beyond the small number of chemicals studied to date. Exposure biomarkers have been developed for > 70 chemicals that caused mammary tumors in rodent studies. These biomarkers can be studied in several existing breast cancer cohort studies that have both biological samples and extensive information on potentially confounding breast cancer risk factors. The exposure biomarkers also can be included in biomonitoring programs in order to identify highly exposed groups for further study and to guide and track exposure reduction. In addition, we identified some knowledge gaps in existing measurements that point to areas for future methods development.

The application of exposure biomarkers for mammary carcinogens requires thoughtful consideration of study design issues (see below). The first two parts of this discussion address interpretation of exposure biomarkers within the exposure-to-disease continuum articulated by Perera and Weinstein (2000). In this framework, exposure biomarkers (e.g., markers of internal dose) and early effect markers (e.g., DNA adducts) are considered intermediate points that can be related back to exposure and forward to disease (Perera and Weinstein 2000). First, we discuss strengths and weaknesses of selecting these chemicals for study based on animal evidence of their potential relationships with human disease. Second, we describe types of exposure biomarkers and factors that influence relationships with exposure sources. Within each of these discussions, we present new research related to novel analyses that move beyond a “one-chemical-at-a-time” approach to consider multiple exposures. Third, we identify the highest priority biomarkers and key study design strategies for breast cancer epidemiology. Fourth, we discuss using exposure biomarkers as tools for breast cancer prevention.

*Relationships between exposure biomarkers and disease*. In this review, we prioritized chemicals to measure in breast cancer studies based on toxicology and laboratory data. Before investing in the biomarkers we identified, researchers will want to consider the evidence that they are relevant to humans, so we discuss that evidence here. Laboratory evidence suggests at least three overlapping classes of chemicals that might increase breast cancer risk: *a*) chemicals that cause mammary gland tumors in animal cancer bioassays, primarily by damaging DNA, *b*) EDCs that accelerate the growth of mammary tumors through estrogenic or other pathways, and *c*) developmental toxicants that can alter development of the mammary gland in ways that permanently increase susceptibility (Brody and Rudel 2008). Many ubiquitous environmental pollutants common in workplaces, consumer products, and building materials fall into one or more of these categories (Brody 2010; Rudel et al. 2007, 2011). Although these classes provide a framework for identifying chemicals of interest, they are based on simplified descriptions of complex biological pathways. For example, although chemicals might disrupt mammary gland development or promote breast tumor formation or growth through estrogenic or genotoxic activity, these effects could also arise from other disruptions in the tissue microenvironment or in cell–cell communications (Boudreau et al. 2012; Sonnenschein and Soto 2013).

Relevance of animal carcinogens to human risk. Animal models of chemically induced cancer are the primary means of understanding and anticipating the effects of chemicals in humans. For pharmaceutical agents, animal studies guide development before human clinical trials can occur. For commercial chemicals and pollutants, particularly when human data are not available, they guide prevention strategies to reduce environmentally associated cancers by reducing exposures (Huff 1996; Rall 2000). The cancer bioassay is designed to identify genotoxic carcinogens. Although some chemicals induce positive responses in this test through nongenotoxic mechanisms, different tests are needed to identify carcinogens that act by promoting growth of existing tumors, by altering tissue structure during development (Birnbaum and Fenton 2003), by otherwise altering the tissue microenvironment (Boudreau et al. 2012), by disrupting cell–cell communication (Sonnenschein and Soto 2013), or by transgenerational epigenetic phenomena (Ruden et al. 2005). Gaps in knowledge about biological pathways that are important in breast development and carcinogenesis limit our ability to anticipate which chemicals may increase risk and to identify the developmental stages with the greatest susceptibility to chemical exposures.

The use of animal studies to identify human carcinogens is supported by observations about the overall concordance of human studies with animal tests. All known human carcinogens that have been adequately tested in animals are also carcinogenic in animal models and have at least one common organ site in both humans and the animal model (Huff 1993, 1999; Huff and Melnick 2006). Historically, about one-third of known human carcinogens were shown to be carcinogenic in animals before being confirmed as carcinogens in epidemiological studies, which means human cancers could have been prevented had exposures been reduced on the basis of animal evidence (Huff and Melnick 2006; IARC 2006). Based on these and other findings, IARC has concluded that “it is biologically plausible that agents for which there is sufficient evidence of carcinogenicity in experimental animals also present a carcinogenic hazard to humans…” and “in the absence of additional scientific information, these agents are considered to pose a carcinogenic hazard to humans” (Cogliano et al. 2011; IARC 2006).

Narrower inferences—that a chemical is not just carcinogenic, but carcinogenic in a specific target organ—can be more tentative because target organs for carcinogens are not necessarily the same across species. Thus, although it is likely that chemicals that cause mammary tumors in rats will also cause tumors in some organs in mice and humans, the mammary gland may not necessarily be the target in humans (Gold et al. 1991; Haseman and Huff 1987). Characterizing the relevance of various rodent models specifically to human breast cancer is still an area of active research (Rudel et al. 2007, 2011); however, at present, these rodent cancer bioassays provide the strongest evidence of potential breast cancer risk factors. In fact, Table 3 shows that there is substantial agreement between studies of human breast cancer and of rat mammary tumors for agents that have been studied in both.

However, not all rodent mammary carcinogens are equally carcinogenic. The chemicals vary in the strength of evidence that they are likely to be human carcinogens, and each must be evaluated with respect to potency, dose response, target sites, tumor incidence and multiplicity, anticipated induction period, and exposure routes and levels in humans. In general, most rodent mammary carcinogens also show evidence of genotoxicity and tumors at other target sites, and these observations support the inference that they would also be carcinogenic in humans (Rudel et al. 2007). However, some mammary carcinogens (e.g., chlordane) induce inconsistent responses in animals, whereas others (e.g., atrazine) induce tumors through mechanisms that reliable biological information suggests are not likely to be relevant in humans (Rudel et al. 2007).

Considering the toxicologic data, it is somewhat puzzling that studies of breast cancer risk associated with tobacco smoking have not been more consistently positive because many tobacco smoke constituents are mammary gland carcinogens, and specific mutations associated with these carcinogens are found at higher concentrations in breast tumors from smokers compared with nonsmokers (Conway et al. 2002). Several major reviews have concluded that exposure to tobacco smoke is associated with higher breast cancer risk (Collishaw et al. 2009; IARC 2012; IOM 2011; Reynolds 2013), but these conclusions have been controversial because many studies have not seen an association (reviewed by Palmer and Rosenberg 1993). In general, breast cancer risk shows the strongest associations with exposure during early life while breast tissue is less differentiated. Using “unexposed” groups that exclude nonsmokers with passive smoke exposure also strengthens the observed associations (Lash and Aschengrau 1999; Reynolds 2013). In addition, researchers have hypothesized that the inconsistent and weak findings may be due to the competing effect of the antiestrogenic activity of tobacco smoke constituents, which would be expected to reduce breast cancer risk (Lash and Aschengrau 1999; MacMahon et al. 1982; Xue et al. 2011). This hypothesis is supported by numerous studies that report unchanged or decreased breast cancer risk in current or recent smokers because the antiestrogenic effects should be strongest near diagnosis. Furthermore, recent results from the Women’s Health Initiative showed a positive association between smoking and postmenopausal breast cancer only among non-obese women (Luo et al. 2011). The authors speculated that the antiestrogenic effects of smoking counteract the effects of the estrogen produced by adipose in obese postmenopausal women.

Although we highlight the agents that have been most thoroughly evaluated in humans, more comprehensive reviews of the epidemiological literature on environmental chemicals and breast cancer are available elsewhere (Brody et al. 2007a; IBCERCC 2013; IOM 2011).

EDCs as potential breast carcinogens. One limitation of our focus on mammary carcinogens is that traditional rodent cancer bioassays may not be sensitive to the effects of EDCs because these studies only dose adult animals, do not evaluate altered susceptibility or tumor promotion, and terminate the studies while the animals are still fairly young, equivalent to about 65 years of age in humans (Huff et al. 2008; Rudel et al. 2011). Some endogenous and pharmaceutical hormones, such as estrogen, progesterone, and diethylstilbestrol, are associated with increased breast cancer risk in humans and in rodents (Cogliano et al. 2011; Hoover et al. 2011), and these observations have raised concerns that EDCs, including common consumer product chemicals that are weak estrogens or have other hormonal activity, could contribute to breast cancer risk (Brody and Rudel 2008; Dodson et al. 2012; Rudel et al. 2003). Although most commercial chemicals have not been screened for endocrine disruption, we identified 22 rodent mammary gland carcinogens that are also EDCs, including amsonic acid, PFOA, and chlordane.

Mixtures. Another limitation is our focus on measures of individual chemicals. A priority for future research is to develop biomarkers that integrate the effects of multiple exposures on the same biological pathway and that detect early effects of chemical exposure. Rather than measuring the level of an individual chemical, these methods can integrate effects of multiple agents and do not depend on a priori knowledge of each potentially relevant chemical. For example, some studies have sought to measure total exposure to exogenous estrogenic chemicals in adipose by using a functional *in vitro* assay for estrogenic activity (Fernandez et al. 2007; Rasmussen et al. 2003), and others have proposed a method to measure protein adducts formed by all reactive electrophiles (Rappaport et al. 2012) or measures of oxidative stress and DNA damage (e.g., Arlt and Schwerdtle 2011).

Similarly, environment-wide association studies (EWAS) adapt the strategies of discovery-based genome-wide association studies (GWAS) to identify environmental chemical exposures linked to disease. For example, Patel et al. (2010, 2013) used NHANES to conduct an EWAS study looking across all measured exposures for associations with diabetes. Because there are so many chemical exposures, and it is impossible to measure each one and anticipate its relationship with disease, these discovery-based techniques are an important tool for generating new hypotheses that can be tested in health studies (Rappaport 2011, 2012).

*Relationships between biomarkers and exposure*. In addition to understanding how biomarkers relate to disease, it is crucial to understand their relationship to exposure sources. Efforts to identify highly exposed populations, develop relevant epidemiological study designs, and reduce exposures all benefit from a deep understanding of the relationship between biomarkers and exposure sources. These relationships are influenced by pharmacokinetics that dictate the chemicals’ absorption, distribution, metabolism, and excretion (ADME). These ADME parameters, taken together with knowledge of exposure pathways, also provide insight into the relative advantages of using biological versus environmental measures for a particular application. A good understanding of ADME is essential for selecting useful biomarkers and interpreting biomarker measurements, and some key considerations are discussed below.

Types of exposure biomarkers: parent, metabolite, reactant/adduct. Exposure to some of the chemicals reviewed here can be assessed by measuring the parent compound. In other cases, especially when the parent compound is quickly metabolized or otherwise difficult to measure, a metabolite or a DNA or protein adduct may be preferable. Because DNA adducts are considered to represent an initial stage in a carcinogenic process, DNA adducts can provide information about biological effects as well as exposure (Izzotti et al. 1999).

For some exposures, the chemical most easily measured as a biomarker is not the biologically active moiety, and this can lead to exposure misclassification. For example, many epidemiological studies have used serum dichlorodiphenyldichloroethylene (DDE) levels as a proxy for exposure to DDT (Brody and Rudel 2008). However, serum DDE levels measured decades after DDT was banned largely reflect exposure to DDE via the food chain and not exposure to the more-active DDT (Snedeker 2001). The DNA-reactive metabolites of many genotoxic compounds are very short lived in the blood and so cannot be reliably measured; in such cases, longer-lasting protein or DNA adducts with the active metabolite often prove more informative. For carcinogenic PAHs, such as BaP, methods to measure the specific genotoxic metabolite independently or as a protein adduct are not sensitive enough for use in the general population, and common, less-specific ELISA methods do not reliably differentiate between exposed and unexposed people (Käfferlein et al. 2010). Urinary metabolites are often used in preference to the parent compound in blood because urine collection is less invasive; however, differences in urine metabolite levels can represent individual differences in metabolic capability in addition to differences in exposure, again leading to exposure misclassification if the biomarker is not the biologically active agent.

Some exposure biomarkers are common metabolites of multiple parent compounds. This can be an advantage in a health study if all the possible parent compounds are believed to act additively and with potency proportional to metabolite levels, but it can be a disadvantage if this is not the case or if the study is trying to characterize exposure to a single parent compound. For example, in NHANES, CDC measurements of HEMA in urine reflect possible exposure to several hazardous chemicals, including vinyl chloride, EtO, and ethylene dibromide (Calafat et al. 1999). Variation in HEMA levels can be associated with variation in exposure to any of the possible parent compounds.

For chemicals that are excreted rapidly, it can be difficult to find biomarkers that reflect a longer-term exposure, especially when exposures are episodic. For example, for chemicals with short biological half-lives, such as bisphenol A (BPA), urine concentrations from one person can vary over the course of a single day because exposure occurs at meals and the compound is rapidly metabolized and excreted (Teeguarden et al. 2011). This daily variation is so great that a single spot urine BPA level may not differentiate longer-term exposure among a group of people. Exposure biomarkers would ideally be biologically persistent enough that concentrations in the biological matrix are detectable and will reflect average exposure rather than time since exposure. Combining multiple spot samples is another way to estimate average exposure to rapidly metabolized chemicals. In other cases, a protein- or DNA-bound metabolite, or an effect marker such as DNA mutation in blood lymphocytes, may be long-lived and represent previous exposure after the parent compound or metabolite can no longer be detected (Manjanatha et al. 1996). For example, the hemoglobin adduct for EtO represents cumulative exposure over several months, whereas the urinary metabolite HEMA is short-lived and nonspecific (Alwis et al. 2012; Ogawa et al. 2006).

Although blood and urine concentrations are generally considered good proxies for internal dose at the target tissue, factors that can influence these relationships should be considered. For example, estradiol concentrations in blood and adipose tissue are not always well correlated (Falk et al. 2012). Similarly, studies of chemically induced DNA adducts and DNA damage in blood and mammary tissue have shown that blood samples are not always representative of levels in mammary tissue (Delclos et al. 1996; Izzotti et al. 1999; Manjanatha et al. 1996).

Blood and urine samples are the most common matrices for exposure monitoring, but methods are available to measure some biomarkers in other matrices such as exhaled breath and breast milk. Breast milk is a valuable medium for exposure monitoring because it can be collected noninvasively at substantial volume, the high fat content captures lipophilic compounds, and it reflects exposures to young women and infants. Other matrices that can be used for biomonitoring include fine-needle aspirates of breast ductal fluid (Mills et al. 2011), hair (e.g., Bessette et al. 2009), adipose tissue (e.g., Covaci et al. 2002; Dewailly et al. 1999; Falk et al. 2012), saliva (e.g., Bessette et al. 2010), exhaled breath (e.g., Cope et al. 2004; Plebani et al. 1999), and fingernails (Esteban and Castaño 2009).

Biological versus environmental measures. In epidemiological studies, biological exposure measures are often preferred to environmental measures such as chemical concentration in drinking water or house dust. In some cases, however, there are advantages to using environmental measures instead or in addition. Biological exposure measures are powerful when they can assess the biologically active component (parent or metabolite) in blood or at the target tissue and during the appropriate exposure window. Exposure misclassification commonly results from uses of biomarkers that do not fulfill these criteria, which can include measuring a component (parent or metabolite) that is not the active moiety (e.g., DDE rather than DDT), measuring in urine rather than blood (questionable proxy for circulating levels given interindividual differences in metabolism and excretion), and measuring after the critical exposure window. In some cases, chemical measurements in exposure media (e.g., drinking water, air, house dust) may provide a reliable estimate of intake. For example, systematically collected data can facilitate the estimation of contaminant levels in the tap water serving large numbers of study participants (Legay et al. 2011). Similarly, measurements of chemicals in house or other indoor dust may be representative of long-term exposure from indoor environments given that pollutants degrade slowly in dust, keeping concentrations in dust relatively constant over time (Quirós-Alcalá et al. 2011). People spend most of their time indoors (U.S. EPA 2011a), making air and dust in indoor environments important sources of exposure to many chemicals used in consumer products and building materials that have been linked to health effects, including cancer. Thus, in some studies it may be useful to collect environmental samples, such as house dust or air, in addition to biological samples.

Research needs. Chemical-specific pharmacokinetic studies, often performed in animals, are important tools for identifying the best exposure biomarkers and understanding relationships between exposures and biomarkers. For example, many researchers used monoethylhexylphthalate as a primary biomarker for diethylhexyl phthalate (DEHP) exposure until ADME studies indicated that it was a minor metabolite (6%) compared with oxidized DEHP metabolites, which represent 70% of excreted DEHP and are longer lived (Koch et al. 2006).

New “omics”-based chemical analytical approaches to exposure assessment offer the opportunity to discover important exposure biomarkers without having to know *a priori* what chemical or metabolite to target (Rappaport 2011, 2012). For example, metabolomics techniques, widely used to investigate pharmaceuticals’ effects on levels of small molecules in blood, have been used to identify exposure biomarkers (Park et al. 2012). These exposome approaches are an especially promising tool for identifying exposure biomarkers for complex mixtures such as vehicle exhaust. Proteomic techniques can also identify adducts as exposure biomarkers.

Many health studies rely on a limited number of available retrospective exposure measures, very few of which provide information about *in utero* exposures. One promising technique involves measuring chemicals in baby teeth, in which it appears chemicals are deposited and retained *in utero* and during early childhood (Camann et al. 2012). Additional development and validation of this approach could permit a wide range of chemicals to be detected and exposure assigned to particular developmental periods based on when particular tooth areas are formed.

*Exposure biomarkers in breast cancer epidemiology*. Given that the best-established risk factors for breast cancer are associated with fairly modest increases in risk (e.g., odds ratios < 2), it is likely that to be sensitive to the effects of environmental chemicals, breast cancer epidemiology will require thoughtful design informed by mechanistic evidence, and many kinds of exposures may ultimately not be suitable for study in humans. One of the most promising opportunities for finding associations is to study exposures to genotoxic carcinogens in younger women (< 50 years of age at diagnosis) with high exposures, and with follow-up of at least 10–20 years. Cohorts with occupational exposures, for example, may have elevations in risk high enough to be detected in epidemiological studies.

For exposures that do lend themselves to epidemiological study, careful choice of biomarkers is essential to capturing the relevant exposures, and in many cases improved biomarkers are needed. Many genotoxic rodent mammary carcinogens are metabolized rapidly, necessitating either repeated measurements or measurements of protein or DNA adducts to reflect exposure over an extended period. Studies of hormone disruption would be strengthened by integrated measures for hormonal activity to augment measurements of individual chemicals, an approach currently hampered by lack of knowledge about what chemicals might affect a given pathway and to what degree. Finally, measures to estimate *in utero* exposures retrospectively among women diagnosed in their 40s–60s could lead to more informative studies of this important life stage.

Epidemiological studies must also consider a number of challenging design problems beyond selecting appropriate exposure measures. Because multiple factors influence breast cancer risk (including many factors that are potentially confounding because they are correlated with demographics that also are related to environmental exposures), building on cohort studies that have already collected information on established risk factors is helpful. Empirical induction periods between exposure and breast cancer diagnosis range from > 50 years for *in utero* exposure to hormones (Hoover et al. 2011) to 10–20 years for classical genotoxic agents to < 5 years for hormone exposure later in life (IOM 2011), creating implications for the length of follow-up needed for studies of environmental chemicals that operate by different mechanisms.

Study designs matched to more sophisticated biological hypotheses will likely prove more informative than studies that do not take into account current understanding of the complexities of breast cancer. Effects of a particular agent may be limited to specific subdiseases within the heterogeneous outcome called “breast cancer.” For example, an analysis of 34 studies found that reproductive factors and body mass index (BMI) are only associated with hormone-receptor-positive tumors and not with basal type (triple negative) tumors (Yang et al. 2011). Other effects appear stronger in subgroups with particular risk factors, as evident in the repeated observations that body size has a stronger effect on the risk of breast cancer among postmenopausal women, and that the association with alcohol intake is stronger among women with certain polymorphisms in enzymes responsible for alcohol metabolism (IOM 2011). An additional example of effect modification is that breast tissue is more sensitive to ionizing radiation before the differentiation that occurs during a woman’s first full term pregnancy (Henderson et al. 2010; Land 1995).

Studies of other outcomes related to breast cancer incidence might avoid some of the difficulties inherent in studies of primary breast cancer incidence. Recurrence and hormonal status of breast cancers, as well as ability to lactate, likely respond to more recent exposures and provide information specific to individual biological pathways and breast cancer types. Assessments of puberty in girls, including age at menarche, hormonal status, and Tanner breast development staging, provide some information about the effects on early-life mammary gland development, which may have a profound impact on breast cancer risk later in life (Rudel et al. 2011). More sophisticated measures of breast development, if identified, would improve researchers’ ability to extrapolate from effects of early-life exposure to later breast cancer risk.

Epidemiological studies that are considering using archived biological samples to measure exposures must also carefully consider the validity of any new measurements. For example, researchers should evaluate both the stability of the analyte under the relevant sample collection and storage procedures as well as the possibility of background contamination from sampling apparatus or containers. Important considerations have been highlighted by Rothman et al. (1995), the Nurses’ Health Study (2014), and Calafat et al. (2013).

Exposure biomarkers as tools for breast cancer prevention. In light of the difficulties inherent in using environmental epidemiology to study cancer risk factors, a recent IARC review of human carcinogens concluded that “every Group 1 agent can be considered to represent cancers that might have been prevented had scientists been able to predict cancer hazards earlier or had public health authorities been willing to act more quickly when scientific information became available” (Cogliano et al. 2011; IARC 2012). This report and others (IBCERCC 2013; IOM 2011; President’s Cancer Panel 2010) all support efforts to use and improve predictive toxicology methods (animal, *in vitro, in silico*) to enable smarter decisions limiting exposures based on biologically plausible effects on breast cancer risk.

Biomonitoring data are becoming critically important in risk-based prioritization exercises that direct exposure control and additional research (Bevan et al. 2012; Rudel and Perovich 2012). Once toxicity testing indicates approximate doses associated with biological effects, comparison with biomonitoring data can help focus on exposures of greatest concern. Based on this extensive review, the chemicals listed in Table 2 and in Supplemental Material, Table S1, are important priorities for biomonitoring of both the general population and highly exposed subpopulations such as occupationally exposed groups. The identification of highly exposed women and men can focus exposure reduction and epidemiological studies on exposures likely to be associated with breast cancer.

By characterizing distributions of exposures in the population, biomonitoring can also provide information on the public health impact of exposure and potential benefits of exposure reduction. For example, Bellinger (2012) used blood lead levels to demonstrate that lead exposure in childhood is responsible for almost as great an impact on the intelligence quotient (IQ), on a population level, as is preterm birth. This dramatic effect arises because although the individual effect on IQ from low-level exposure is small, exposure is widespread. Similarly, widespread exposure to chemicals plausibly linked with breast cancer could be responsible for many preventable breast cancers.

Estimates of breast cancer risk attributable to well-established risk factors such as HRT, alcohol, physical inactivity, reproductive history, and family history of breast cancer can provide some context for considering the potential benefits of reducing chemical exposure. One model estimated that by optimizing BMI, alcohol consumption, and physical activity, the 20-year absolute breast cancer risk for an average 45-year-old in a population of Italian women would be reduced from 6.5% to 5.1%, with larger reductions among women with more risk factors (Petracci et al. 2011). Population attributable risk (PAR) estimates, which provide a sense of the relative difference in breast cancer risk between the whole population and the unexposed portion of the population, vary widely across studies of established risk factors, including alcohol (2–11%), HRT (4–27%), and physical inactivity (6–20%) (IOM 2011). A German study estimated a breast cancer PAR for a combination of less-modifiable risk factors (age at menarche/menopause, parity, benign breast disease, family breast cancer history) at 37% (Barnes et al. 2011).

Estimating PARs for rodent mammary carcinogens is difficult because of the lack of evidence regarding both the strength of the association between exposure and disease and the prevalence of the exposures. Although few rodent mammary carcinogens have been studied in humans—and the studies have methodological weaknesses—increased odds range from 30% to 400%, which is within the range observed for the established risk factors (Brody et al. 2007a; Cohn et al. 2007). The opportunity for breast cancer prevention by reducing exposure to chemicals such as the rodent mammary carcinogens is significant because animal evidence suggests that some are potent carcinogens, widespread exposures to many have been established, and populations highly exposed to others may be identified in future surveillance. Furthermore, because public and industrial policies drive exposure to many rodent mammary carcinogens, exposure information is needed to inform public health decision making.

## Conclusions

This review substantially broadens directions for studying chemicals and breast cancer by compiling biomarker measurement techniques for 102 plausible breast carcinogens and prioritizing 17 chemical groups for study, biomonitoring, and exposure reduction. These priorities include components of automobile exhaust, gasoline, and air pollution (1,3-butadiene, benzene, PAHs, nitro-PAHs), chemicals in food and drinking water (acrylamide, ochratoxin A, heterocyclic amines, styrene, MX), chemicals in consumer products and building materials (flame retardants, aromatic amines, PFCs), pharmaceuticals, EDCs, and some chemicals with important occupational exposures (halogenated solvents, EtO).

We found that exposure measurement methods are available for almost three-quarters of the 102 rodent mammary gland carcinogens evaluated, and some analytes could be assessed using existing methods for related chemicals. Methods have been published for 73 of the 102 chemicals. Exposure biomarkers for 62 have been measured in humans, and 45 were measured in a nonoccupationally exposed population, including 23 measured by the CDC in the general U.S. population. In some cases, analytical methods for biological samples permit the measurement of many analytes in a single sample. Epidemiological studies of breast cancer risk are generally consistent with rodent bioassays, although only a few agents have been studied in humans. We identified 42 cohort studies with a total of > 3.5 million enrolled women that have ascertained breast cancer incidence as an outcome measure and have collected biological samples, presenting numerous opportunities to apply novel exposure measures in breast cancer epidemiology. Biomonitoring programs following the priorities we have laid out could effectively assess exposure and identify highly exposed groups, enabling the development of strategies to prevent breast cancer by reducing exposure to plausible breast carcinogens.

## Supplemental Material

(815 KB) PDF

## References

[r1] Alwis KU, Blount BC, Britt AS, Patel D, Ashley DL (2012). Simultaneous analysis of 28 urinary VOC metabolites using ultra high performance liquid chromatography coupled with electrospray ionization tandem mass spectrometry (UPLC-ESI/MSMS).. Anal Chim Acta.

[r2] Ambrosone CB, Abrams SM, Gorlewska-Roberts K, Kadlubar FF (2007). Hair dye use, meat intake, and tobacco exposure and presence of carcinogen-DNA adducts in exfoliated breast ductal epithelial cells.. Arch Biochem Biophys.

[r3] American Cancer Society. (2010). The Global Economic Cost of Cancer.. http://www.cancer.org/acs/groups/content/@internationalaffairs/documents/document/acspc-026203.pdf.

[r4] Arlt VM, Schwerdtle T (2011). UKEMS/Dutch EMS-sponsored workshop on biomarkers of exposure and oxidative DNA damage & 7th GUM-32P-postlabelling workshop, University of Munster, Munster, Germany, 28–29 March 2011.. Mutagenesis.

[r5] Barnes BB, Steindorf K, Hein R, Flesch-Janys D, Chang-Claude J (2011). Population attributable risk of invasive postmenopausal breast cancer and breast cancer subtypes for modifiable and non-modifiable risk factors.. Cancer Epidemiol.

[r6] Barr DB, Barr JR, Bailey SL, Lapeza CR, Beeson MD, Caudill SP (2000). Levels of methyleugenol in a subset of adults in the general U.S. population as determined by high resolution mass spectrometry.. Environ Health Perspect.

[r7] Bellinger DC 2012 A strategy for comparing the contributions of environmental chemicals and other risk factors to neurodevelopment of children. Environ Health Perspect 120 501 507; 10.1289/ehp.1104170 22182676 PMC3339460

[r8] Bessette EE, Spivack SD, Goodenough AK, Wang T, Pinto S, Kadlubar FF (2010). Identification of carcinogen DNA adducts in human saliva by linear quadrupole ion trap/multistage tandem mass spectrometry.. Chem Res Toxicol.

[r9] Bessette EE, Yasa I, Dunbar D, Wilkens LR, Le Marchand L, Turesky RJ (2009). Biomonitoring of carcinogenic heterocyclic aromatic amines in hair: a validation study.. Chem Res Toxicol.

[r10] Bevan R, Angerer J, Cocker J, Jones K, Koch HM, Sepai O (2012). Framework for the development and application of environmental biological monitoring guidance values.. Regul Toxicol Pharmacol.

[r11] Birnbaum LS, Fenton SE (2003). Cancer and developmental exposure to endocrine disruptors.. Environ Health Perspect.

[r12] Blair IA (2010). Analysis of estrogens in serum and plasma from postmenopausal women: past present, and future.. Steroids.

[r13] Blount BC, Kobelski RJ, McElprang DO, Ashley DL, Morrow JC, Chambers DM (2006). Quantification of 31 volatile organic compounds in whole blood using solid-phase microextraction and gas chromatography-mass spectrometry.. J Chromatogr B Analyt Technol Biomed Life Sci.

[r14] Blount BC, McElprang DO, Chambers DM, Waterhouse MG, Squibb KS, Lakind JS (2010). Methodology for collecting, storing, and analyzing human milk for volatile organic compounds.. J Environ Monit.

[r15] Blum A, Gold MD, Ames BN, Jones FR, Hett EA, Dougherty RC (1978). Children absorb tris-BP flame retardant from sleepwear: urine contains the mutagenic metabolite, 2,3-dibromopropanol.. Science.

[r16] Boogaard PJ (2002). Use of haemoglobin adducts in exposure monitoring and risk assessment.. J Chromatogr B Analyt Technol Biomed Life Sci.

[r17] Boudreau A, van’t Veer LJ, Bissell MJ (2012). An “elite hacker”: breast tumors exploit the normal microenvironment program to instruct their progression and biological diversity.. Cell Adh Migr.

[r18] Boysen G, Hecht SS (2003). Analysis of DNA and protein adducts of benzo[*a*]pyrene in human tissues using structure-specific methods.. Mutat Res.

[r19] Brody J (2010). Everyday exposures and breast cancer.. Rev Environ Health.

[r20] Brody JG, Moysich KB, Humblet O, Attfield KR, Beehler GP, Rudel RA (2007a). Environmental pollutants and breast cancer: epidemiologic studies.. Cancer.

[r21] Brody JG, Rudel RA (2008). Environmental pollutants and breast cancer: the evidence from animal and human studies.. Breast Dis Year Book Q.

[r22] Brody JG, Rudel RA, Michels KB, Moysich KB, Bernstein L, Attfield KR (2007b). Environmental pollutants, diet, physical activity, body size, and breast cancer: where do we stand in research to identify opportunities for prevention?. Cancer.

[r23] Brown NM, Manzolillo PA, Zhang JX, Wang J, Lamartiniere CA (1998). Prenatal TCDD and predisposition to mammary cancer in the rat.. Carcinogenesis.

[r24] Budavari S, O’Neil M, Smith A, P. Heckelman P, Obenchain J. (1996). The Merck Index. 12th ed. An Encyclopedia of Chemicals, Drugs and Biologicals..

[r25] Calafat AM, Barr DB, Pirkle JL, Ashley DL (1999). Reference range concentrations of *N*-acetyl-*S*-(2-hydroxyethyl)-l-cysteine, a common metabolite of several volatile organic compounds, in the urine of adults in the United States.. J Expo Anal Environ Epidemiol.

[r26] Calafat AM Koch HM Swan SH Hauser R Goldman LR Lanphear BP 2013 Misuse of blood serum to assess exposure to bisphenol A and phthalates [Letter] Breast Cancer Res 15 403 24083327 10.1186/bcr3494 PMC3978629

[r27] California OEHHA (California Office of Environmental Health Hazard Assessment). (2014). Proposition 65—Current Proposition 65 List [06/06/14].. http://www.oehha.ca.gov/prop65/prop65_list/Newlist.html.

[r28] Camann DE, Schultz ST, Yau AY, Heilbrun LP, Zuniga MM, Palmer RF (2012). Acetaminophen, pesticide, and diethylhexyl phthalate metabolites, anandamide, and fatty acids in deciduous molars: potential biomarkers of perinatal exposure.. J Expo Sci Environ Epidemiol.

[r29] Cao Z, Swift TA, West CA, Rosano TG, Rej R (2004). Immunoassay of estradiol: unanticipated suppression by unconjugated estriol.. Clin Chem.

[r30] Cardiff RD, Bern HA, Faulkin LJ, Daniel CW, Smith GH, Young LJ (2002). Contributions of mouse biology to breast cancer research.. Comp Med.

[r31] Carmella SG, Chen M, Han S, Briggs A, Jensen J, Hatsukami DK (2009). Effects of smoking cessation on eight urinary tobacco carcinogen and toxicant biomarkers.. Chem Res Toxicol.

[r32] CDC (Centers For Disease Control and Prevention). (2008). 2003–2004 Data Documentation, Code Book, And Frequencies: Acrylamide And Glycidamide (L06Age_C).. http://wwwn.cdc.gov/nchs/nhanes/2003-2004/L06AGE_C.htm.

[r33] CDC (Centers For Disease Control and Prevention). (2009). Fourth National Report on Human Exposure to Environmental Chemicals.. http://www.cdc.gov/exposurereport/pdf/FourthReport.pdf.

[r34] CDC (Centers For Disease Control and Prevention). (2012). Fourth National Report on Human Exposure to Environmental Chemicals: Updated Tables, February 2012.. http://www.cdc.gov/exposurereport/pdf/FourthReport_UpdatedTables_Feb2012.pdf.

[r35] CDC (Centers For Disease Control and Prevention). (2014). National Health and Nutrition Examination Survey.. http://www.cdc.gov/nchs/nhanes.htm.

[r36] Cogliano VJ, Baan R, Straif K, Grosse Y, Lauby-Secretan B, El Ghissassi F (2011). Preventable exposures associated with human cancers.. J Natl Cancer Inst.

[r37] Cohn BA Wolff MS Cirillo PM Sholtz RI 2007 DDT and breast cancer in young women: new data on the significance of age at exposure. Environ Health Perspect 115 1406 1414; 10.1289/ehp.10260 17938728 PMC2022666

[r38] Collishaw NE, Boyd NF, Cantor KP, Hammond SK, Johnson KC, Millar J, et al. (2009). Canadian Expert Panel on Tobacco Smoke and Breast Cancer Risk..

[r39] Conway K, Edmiston SN, Cui L, Drouin SS, Pang J, He M (2002). Prevalence and spectrum of p53 mutations associated with smoking in breast cancer.. Cancer Res.

[r40] Cooper EM, Covaci A, van Nuijs AL, Webster TF, Stapleton HM (2011). Analysis of the flame retardant metabolites bis(1,3-dichloro-2-propyl) phosphate (BDCPP) and diphenyl phosphate (DPP) in urine using liquid chromatography-tandem mass spectrometry.. Anal Bioanal Chem.

[r41] Cope KA, Watson MT, Foster WM, Sehnert SS, Risby TH (2004). Effects of ventilation on the collection of exhaled breath in humans.. J Appl Physiol.

[r42] Coronado GD, Holte S, Vigoren E, Griffith WC, Barr DB, Faustman E (2011). Organophosphate pesticide exposure and residential proximity to nearby fields: evidence for the drift pathway.. J Occup Environ Med.

[r43] Covaci A, de Boer J, Ryan JJ, Voorspoels S, Schepens P (2002). Distribution of organobrominated and organochlorinated contaminants in Belgian human adipose tissue.. Environ Res.

[r44] Czene K, Osterman-Golkar S, Yun X, Li G, Zhao F, Pérez HL (2002). Analysis of DNA and hemoglobin adducts and sister chromatid exchanges in a human population occupationally exposed to propylene oxide: a pilot study.. Cancer Epidemiol Biomarkers Prev.

[r45] De Alwis GKH, Needham LL, Barr DB (2007). Automated solid phase extraction and quantitative measurement of 2,3-dibromo-1-propanol in urine using gas chromatography-mass spectrometry.. Arch Environ Contam Toxicol.

[r46] Delclos KB, Blaydes B, Heflich RH, Smith BA (1996). Assessment of DNA adducts and the frequency of 6-thioguanine resistant T-lymphocytes in F344 rats fed 2,4-toluenediamine or implanted with a toluenediisocyanate-containing polyester polyurethane foam.. Mutat Res.

[r47] Dewailly É, Mulvad G, Pedersen HS, Ayotte P, Demers A, Weber JP (1999). Concentration of organochlorines in human brain, liver, and adipose tissue autopsy samples from Greenland.. Environ Health Perspect.

[r48] DHHS (Department of Health and Human Services). (2004). Household Products Database: Health and Safety Information on Household Products Chemical Information.. http://householdproducts.nlm.nih.gov/.

[r49] D’Hollander W, de Voogt P, De Coen W, Bervoets L (2010). Perfluorinated substances in human food and other sources of human exposure.. Rev Environ Contam Toxicol.

[r50] Dodson RE, Houseman EA, Levy JI, Spengler JD, Shine JP, Bennett DH (2007). Measured and modeled personal exposures to and risks from volatile organic compounds.. Environ Sci Technol.

[r51] Dodson RE Nishioka M Standley LJ Perovich LJ Brody JG Rudel RA 2012 Endocrine disruptors and asthma-associated chemicals in consumer products. Environ Health Perspect 120 935 943; 10.1289/ehp.1104052 22398195 PMC3404651

[r52] ECHA (European Chemicals Agency). (2013). Candidate List of Substances of Very High Concern for Authorisation.. http://echa.europa.eu/candidate-list-table.

[r53] Environmental Defense Fund. (2004). Scorecard: The Pollution Information Site.. https://web.archive.org/web/20050730082500/http://www.scorecard.org/.

[r54] Environmental Health Risk in European Birth Cohorts (ENRIECO). (2010). Inventory of ENRIECO Cohorts.. http://birthcohortsenrieco.net.

[r55] Esteban M, Castaño A (2009). Non-invasive matrices in human biomonitoring: a review.. Environ Int.

[r56] Falk RT, Gentzschein E, Stanczyk FZ, Garcia-Closas M, Figueroa JD, Ioffe OB (2012). Sex steroid hormone levels in breast adipose tissue and serum in postmenopausal women.. Breast Cancer Res Treat.

[r57] Fernandez MF, Santa-Marina L, Ibarluzea JM, Exposito J, Aurrekoetxea JJ, Torne P (2007). Analysis of population characteristics related to the total effective xenoestrogen burden: a biomarker of xenoestrogen exposure in breast cancer.. Eur J Cancer.

[r58] Food and Drug Administration. (2005). “Everything” Added to Food in the United States (EAFUS): A Food Additive Database.. http://vm.cfsan.fda.gov/?dms/eafus.html.

[r59] Friedman GD, Jiang SF, Udaltsova N, Chan J, Quesenberry CP, Habel LA (2009). Pharmaceuticals that cause mammary gland tumors in animals: findings in women.. Breast Cancer Res Treat.

[r60] Gold LS, Slone TH, Manley NB, Bernstein L (1991). Target organs in chronic bioassays of 533 chemical carcinogens.. Environ Health Perspect.

[r61] Gold L, Slone TH, Manley NB, Garfinkel GB, Ames BN. (2005). Summary of Carcinogenic Potency Database by Target Organ.. http://toxnet.nlm.nih.gov/cpdb/pdfs/CPDBPathology.pdf.

[r62] Haseman J, Huff J (1987). Species correlation in long-term carcinogenicity studies.. Cancer Lett.

[r63] Health Canada. (2001). Canadian Environmental Protection Act, 1999: Priority Substances List Assessment Report: Ethylene Oxide. 0-662-28980-3.. http://publications.gc.ca/collections/Collection/En40-215-51E.pdf.

[r64] Henderson TO, Amsterdam A, Bhatia S, Hudson MM, Meadows AT, Neglia JP (2010). Systematic review: surveillance for breast cancer in women treated with chest radiation for childhood, adolescent, or young adult cancer.. Ann Intern Med.

[r65] Hester TR, Ford NF, Gale PJ, Hammett JL, Raymond R, Turnbull D (1997). Measurement of 2,4-toluenediamine in urine and serum samples from women with Même or Replicon breast implants.. Plast Reconstr Surg.

[r66] Hoehle SI, Knudsen GA, Sanders JM, Sipes IG (2009). Absorption, distribution, metabolism, and excretion of 2,2-bis(bromomethyl)-1,3-propanediol in male Fischer-344 rats.. Drug Metab Dispos.

[r67] Hooper DG, Bolton VE, Guilford FT, Straus DC (2009). Mycotoxin detection in human samples from patients exposed to environmental molds.. Int J Mol Sci.

[r68] Hoover RN, Hyer M, Pfeiffer RM, Adam E, Bond B, Cheville AL (2011). Adverse health outcomes in women exposed in utero to diethylstilbestrol.. N Engl J Med.

[r69] Huff J (1993). Chemicals and cancer in humans: first evidence in experimental animals.. Environ Health Perspect.

[r70] Huff J. (1996). Chemically induced cancers in hormonal organs of laboratory animals and of humans.

[r71] Huff J 1999 Animal and human carcinogens [Letter] Environ Health Perspect 107 A341 A342 10405250 10.1289/ehp.107-1566684 PMC1566684

[r72] Huff J Jacobson MF Davis DL 2008 The limits of two-year bioassay exposure regimens for identifying chemical carcinogens. Environ Health Perspect 116 1439 1442; 10.1289/ehp.10716 19057693 PMC2592260

[r73] Huff J, Melnick R (2006). What are the real causes of cancer?. Int J Occup Environ Health.

[r74] Huyck S, Ohman-Strickland P, Zhang L, Tong J, Xu XU, Zhang JJ (2010). Determining times to maximum urine excretion of 1-aminopyrene after diesel exhaust exposure.. J Expo Sci Environ Epidemiol.

[r75] IARC (International Agency for Research on Cancer). (1993). Ochratoxin A.. IARC Monogr Eval Carcinog Risks Hum.

[r76] IARC (International Agency for Research on Cancer). (2002). Some Traditional Herbal Medicines, Some Mycotoxins, Naphthalene and Styrene.. IARC Monogr Eval Carcinog Risks Hum.

[r77] IARC (International Agency for Research on Cancer). (2006). Preamble to the IARC Monographs on the Evaluation of Carcinogenic Risks to Humans.. http://monographs.iarc.fr/ENG/Preamble/CurrentPreamble.pdf.

[r78] IARC (International Agency for Research on Cancer). (2010). Identification of Research Needs to Resolve the Carcinogenicity of High-Priority IARC Carcinogens. Technical Publication No. 42.. http://monographs.iarc.fr/ENG/Publications/techrep42/TR42-Full.pdf.

[r79] IARC (International Agency for Research on Cancer). (2012). A Review of Human Carcinogens.. IARC Monogr Eval Carcinog Risks Hum.

[r80] IBCERCC (Interagency Breast Cancer & Environmental Research Coordinating Committee). (2013). Breast Cancer and the Environment: Prioritizing Prevention.. http://www.niehs.nih.gov/about/boards/ibcercc/.

[r81] Il’yasova D, McCarthy BJ, Erdal S, Shimek J, Goldstein J, Doerge DR (2009). Human exposure to selected animal neurocarcinogens: a biomarker-based assessment and implications for brain tumor epidemiology.. J Toxicol Environ Health B Crit Rev.

[r82] IOM (Institute of Medicine). (2011). Breast Cancer and the Environment: A Life Course Approach. Washington DC:National Academies Press.. http://www.iom.edu/Reports/2011/Breast-Cancer-and-the-Environment-A-Life-Course-Approach.aspx.

[r83] Izzotti A, Camoirano A, Cartiglia C, Grubbs CJ, Lubet RA, Kelloff GJ (1999). Patterns of DNA adduct formation in liver and mammary epithelial cells of rats treated with 7,12-dimethylbenz(*a*)anthracene, and selective effects of chemopreventive agents.. Cancer Res.

[r84] Jones CR, Liu YY, Sepai O, Yan H, Sabbioni G (2005). Hemoglobin adducts in workers exposed to nitrotoluenes.. Carcinogenesis.

[r85] Käfferlein HU, Marczynski B, Mensing T, Brüning T (2010). Albumin and hemoglobin adducts of benzo[*a*]pyrene in humans—analytical methods, exposure assessment, and recommendations for future directions.. Crit Rev Toxicol.

[r86] Koch HM, Preuss R, Angerer J (2006). Di(2-ethylhexyl)phthalate (DEHP): human metabolism and internal exposure—an update and latest results.. Int J Androl.

[r87] Kong W, Kuester RK, Gallegos A, Sipes IG (2011). Induction of DNA damage in human urothelial cells by the brominated flame retardant 2,2-bis(bromomethyl)-1,3-propanediol: role of oxidative stress.. Toxicology.

[r88] Land CE (1995). Studies of cancer and radiation dose among atomic bomb survivors. The example of breast cancer.. JAMA.

[r89] Lash TL, Aschengrau A (1999). Active and passive cigarette smoking and the occurrence of breast cancer.. Am J Epidemiol.

[r90] Lau C, Anitole K, Hodes C, Lai D, Pfahles-Hutchens A, Seed J (2007). Perfluoroalkyl acids: a review of monitoring and toxicological findings.. Toxicol Sci.

[r91] Laumbach R, Tong J, Zhang L, Ohman-Strickland P, Stern A, Fiedler N (2009). Quantification of 1-aminopyrene in human urine after a controlled exposure to diesel exhaust.. J Environ Monit.

[r92] Legay C, Rodriguez MJ, Miranda-Moreno L, Serodes JB, Levallois P (2011). Multi-level modelling of chlorination by-product presence in drinking water distribution systems for human exposure assessment purposes.. Environ Monit Assess.

[r93] Li Z, Sandau CD, Romanoff LC, Caudill SP, Sjodin A, Needham LL (2008). Concentration and profile of 22 urinary polycyclic aromatic hydrocarbon metabolites in the US population.. Environ Res.

[r94] Luo J, Horn K, Ockene JK, Simon MS, Stefanick ML, Tong E (2011). Interaction between smoking and obesity and the risk of developing breast cancer among postmenopausal women: the Women’s Health Initiative Observational Study.. Am J Epidemiol.

[r95] MacMahon B, Trichopoulos D, Cole P, Brown J (1982). Cigarette smoking and urinary estrogens.. N Engl J Med.

[r96] Manjanatha MG, Lyn-Cook LE, Culp SJ, Beland FA, Heflich RH, Aidoo A (1996). Lymphocyte mutant frequency in relation to DNA adduct formation in rats treated with tumorigenic doses of the mammary gland carcinogen 7,12-dimethylbenz[*a*]anthracene.. Mutat Res.

[r97] McDonald TA, Komulainen H (2005). Carcinogenicity of the chlorination disinfection by-product MX.. J Environ Sci Health C Environ Carcinog Ecotoxicol Rev.

[r98] Michels KB, Mohllajee AP, Roset-Bahmanyar E, Beehler GP, Moysich KB (2007). Diet and breast cancer: a review of the prospective observational studies.. Cancer.

[r99] Mills D, Gordon EJ, Casano A, Lahti SM, Nguyen T, Preston A (2011). The physiology of the normal human breast: an exploratory study.. J Physiol Biochem.

[r100] Muñoz K, Blaszkewicz M, Degen GH (2009). Simultaneous analysis of ochratoxin A and its major metabolite ochratoxin alpha in plasma and urine for an advanced biomonitoring of the mycotoxin.. J Chromatogr B Analyt Technol Biomed Life Sci.

[r101] National Cancer Institute. (2013). EGRP-Supported Cancer Epidemiology Consortia.. http://epi.grants.cancer.gov/Consortia.

[r102] National Cancer Institute. (2014). Cancer Control and Population Sciences: Biospecimen Resources for Population Scientists.. http://epi.grants.cancer.gov/biospecimens.html.

[r103] National Institute for Occupational Safety and Health. (1990). National Occupational Exposure Survey (NOES) (1981–1983).. http://www.cdc.gov/noes/default.html.

[r104] NIH (National Institutes of Health). (2009). NIH Computer Retrieval of Information on Scientific Projects.. http://crisp.cit.nih.gov/crisp.

[r105] NIH (National Institutes of Health). (2012). NIH RePORTER.. http://projectreporter.nih.gov/reporter.cfm.

[r106] NTP (National Toxicology Program). (1978). *Technical Report 131*: Bioassays of DDT, TDE, and p,p´-DDE for Possible Caracinogenicity. CAS No. 50-29-3, 72-54-8, 72-55-9. NCI-CG-TR-131. TR-131.. http://ntp.niehs.nih.gov/ntp/htdocs/LT_rpts/tr133.pdf.

[r107] NTP (National Toxicology Program). (2002). Tetrabromobisphenol A bis(2,3-Dibromopropyl Ether) [21850-44-2]: Review of Toxicological Literature. Research Triangle Park, NC:NTP.. http://ntp.niehs.nih.gov/ntp/htdocs/Chem_Background/ExSumPdf/TBBPA-BDPE_508.pdf.

[r108] NTP (National Toxicology Program). (2005a). 11th Report on Carcinogens.. http://ntp.niehs.nih.gov/ntp/roc/toc11.htm.

[r109] NTP (National Toxicology Program). (2005b). Study Reports and Abstracts Collection.. http://ntpsearch.niehs.nih.gov/texis/search/?pr=ntp_web_entire_site_all&mu=Study+Reports+and+Abstracts.

[r110] NTP (National Toxicology Program). (2011). Report on Carcinogens: Twelfth Edition, 2011.. http://ntp.niehs.nih.gov/ntp/roc/twelfth/roc12.pdf.

[r111] Nurses’ Health Study. (2014). Guidelines for External Collaborators: Use of the Nurses’ Health Studies Biospecimens.. http://www.channing.harvard.edu/nhs/?page_id=476.

[r112] Occupational Safety and Health Administration. (2012). 1,3-Butadiene.. http://www.osha.gov/SLTC/butadiene.

[r113] Ogawa M, Oyama T, Isse T, Yamaguchi T, Murakami T, Endo Y (2006). Hemoglobin adducts as a marker of exposure to chemical substances, especially PRTR class I designated chemical substances.. J Occup Health.

[r114] Oyesanmi O, Snyder D, Sullivan N, Reston J, Treadwell J, Schoelles KM. (2010). Alcohol Consumption and Cancer Risk: Understanding Possible Causal Mechanisms for Breast and Colorectal Cancers. Evidence Report/Technology Assessment No. 197. ECRI Institute Evidence-based Practice Center. AHRQ Publication No. 11-E003.

[r115] Palmer JR, Rosenberg L (1993). Cigarette smoking and the risk of breast cancer.. Epidemiol Rev.

[r116] Park YH, Lee K, Soltow QA, Strobel FH, Brigham KL, Parker RE (2012). High-performance metabolic profiling of plasma from seven mammalian species for simultaneous environmental chemical surveillance and bioeffect monitoring.. Toxicology.

[r117] Patel CJ Bhattacharya J Butte AJ 2010 An Environment-Wide Association Study (EWAS) on type 2 diabetes mellitus. PLoS One 5 e10746; 10.1371/journal.pone.0010746 20505766 PMC2873978

[r118] Patel C, Chen R, Kodama K, Ioannidis J, Butte A (2013). Systematic identification of interaction effects between genome-and environment-wide associations in type 2 diabetes mellitus.. Hum Genet.

[r119] Perera FP, Weinstein IB (2000). Molecular epidemiology: recent advances and future directions.. Carcinogenesis.

[r120] Pesticide Action Network. (2000). Pesticide Action Network Pesticides Database.. http://www.pesticideinfo.org.

[r121] Petracci E, Decarli A, Schairer C, Pfeiffer RM, Pee D, Masala G (2011). Risk factor modification and projections of absolute breast cancer risk.. J Natl Cancer Inst.

[r122] Plebani C, Tranfo G, Salerno A, Panebianco A, Marcelloni AM (1999). An optimized sampling and GC-MS analysis method for benzene in exhaled breath, as a biomarker for occupational exposure.. Talanta.

[r123] President’s Cancer Panel. (2010). Reducing Environmental Cancer Risk, What We Can Do Now. Bethesda, MD:National Institutes of Health National Cancer Institute.. http://deainfo.nci.nih.gov/advisory/pcp/annualReports/pcp08-09rpt/PCP_Report_08-09_508.pdf.

[r124] Quirós-Alcalá L Bradman A Nishioka M Harnly ME Hubbard A McKone TE 2011 Pesticides in house dust from urban and farmworker households in California: an observational measurement study. Environ Health 10 19; 10.1186/1476-069X-10-19 21410986 PMC3071308

[r125] Rad G, Hoehle SI, Kuester RK, Sipes IG (2010). In vitro glucuronidation of 2,2-bis(bromomethyl)-1,3-propanediol by microsomes and hepatocytes from rats and humans.. Drug Metab Dispos.

[r126] Rall DP (2000). Laboratory animal tests and human cancer.. Drug Metab Rev.

[r127] Rappaport SM (2011). Implications of the exposome for exposure science.. J Expo Sci Environ Epidemiol.

[r128] Rappaport SM (2012). Biomarkers intersect with the exposome.. Biomarkers.

[r129] Rappaport SM, Li H, Grigoryan H, Funk WE, Williams ER (2012). Adductomics: characterizing exposures to reactive electrophiles.. Toxicol Lett.

[r130] Rasmussen TH Nielsen F Andersen HR Nielsen JB Weihe P Grandjean P 2003 Assessment of xenoestrogenic exposure by a biomarker approach: application of the E-Screen bioassay to determine estrogenic response of serum extracts. Environ Health 2 12; 10.1186/1476-069X-2-12 14613489 PMC270076

[r131] Reynolds P (2013). Smoking and breast cancer.. J Mammary Gland Biol Neoplasia.

[r132] Richardson SD, Plewa MJ, Wagner ED, Schoeny R, Demarini DM (2007). Occurrence, genotoxicity, and carcinogenicity of regulated and emerging disinfection by-products in drinking water: a review and roadmap for research.. Mutat Res.

[r133] Riederer AM Bartell SM Barr DB Ryan PB 2008 Diet and nondiet predictors of urinary 3-phenoxybenzoic acid in NHANES 1999–2002. Environ Health Perspect 116 1015 1022; 10.1289/ehp.11082 18709153 PMC2516573

[r134] Rosner W, Hankinson SE, Sluss PM, Vesper HW, Wierman ME (2013). Challenges to the measurement of estradiol: an endocrine society position statement.. J Clin Endocrinol Metab.

[r135] Rothman N, Stewart WF, Schulte PA (1995). Incorporating biomarkers into cancer epidemiology: a matrix of biomarker and study design categories.. Cancer Epidemiol Biomarkers Prev.

[r136] Rudel RA, Attfield KR, Schifano JN, Brody JG (2007). Chemicals causing mammary gland tumors in animals signal new directions for epidemiology, chemicals testing, and risk assessment for breast cancer prevention.. Cancer.

[r137] Rudel RA, Camann DE, Spengler JD, Korn LR, Brody JG (2003). Phthalates, alkylphenols, pesticides, polybrominated diphenyl ethers, and other endocrine-disrupting compounds in indoor air and dust.. Environ Sci Technol.

[r138] Rudel RA Fenton SE Ackerman JM Euling SY Makris SL 2011 Environmental exposures and mammary gland development: state of the science, public health implications, and research recommendations. Environ Health Perspect 119 1053 1061; 10.1289/ehp.1002864 21697028 PMC3237346

[r139] Rudel R Perovich L 2012 Accurate risk-based chemical screening relies on robust exposure estimates [Letter] Toxicol Sci 128 295 296 22523230 10.1093/toxsci/kfs143

[r140] Ruden DM, Xiao L, Garfinkel MD, Lu X (2005). Hsp90 and environmental impacts on epigenetic states: a model for the trans-generational effects of diethylstilbesterol on uterine development and cancer.. Hum Mol Genet.

[r141] Russo IH, Russo J (1996). Mammary gland neoplasia in long-term rodent studies.. Environ Health Perspect.

[r142] Russo J, Russo IH (1993). Development pattern of human breast and susceptibility to carcinogenesis.. Eur J Cancer Prev.

[r143] Russo J, Russo IH. (2004). Molecular Basis of Breast Cancer..

[r144] Sanchez MN, Garcia EH, Pavon JL, Cordero BM (2012). Fast analytical methodology based on mass spectrometry for the determination of volatile biomarkers in saliva.. Anal Chem.

[r145] Savitz DA (2012). Invited commentary: biomarkers of exposure to drinking water disinfection by-products—are we ready yet?. Am J Epidemiol.

[r146] Schettgen T, Müller J, Fromme H, Angerer J (2010). Simultaneous quantification of haemoglobin adducts of ethylene oxide, propylene oxide, acrylonitrile, acrylamide and glycidamide in human blood by isotope-dilution GC/NCI-MS/MS.. J Chromatogr B Analyt Technol Biomed Life Sci.

[r147] Scott PM (2005). Biomarkers of human exposure to ochratoxin A.. Food Addit Contam.

[r148] Shantakumar S, Gammon MD, Eng SM, Sagiv SK, Gaudet MM, Teitelbaum SL (2005). Residential environmental exposures and other characteristics associated with detectable PAH-DNA adducts in peripheral mononuclear cells in a population-based sample of adult females.. J Expo Anal Environ Epidemiol.

[r149] Snedeker SM (2001). Pesticides and breast cancer risk: a review of DDT, DDE, and dieldrin.. Environ Health Perspect.

[r150] Sonnenschein C, Soto AM (2013). The aging of the 2000 and 2011 Hallmarks of Cancer reviews: a critique.. J Biosci.

[r151] Stanczyk FZ, Clarke NJ (2010). Advantages and challenges of mass spectrometry assays for steroid hormones.. J Steroid Biochem Mol Biol.

[r152] Stevens RG (2009). Light-at-night, circadian disruption and breast cancer: assessment of existing evidence.. Int J Epidemiol.

[r153] Swenberg JA, Bordeerat NK, Boysen G, Carro S, Georgieva NI, Nakamura J (2011). 1,3-Butadiene: biomarkers and application to risk assessment.. Chem Biol Interact.

[r154] Teeguarden JG, Calafat AM, Ye X, Doerge DR, Churchwell MI, Gunawan R (2011). Twenty-four hour human urine and serum profiles of bisphenol a during high-dietary exposure.. Toxicol Sci.

[r155] Toriba A, Kitaoka H, Dills RL, Mizukami S, Tanabe K, Takeuchi N (2007). Identification and quantification of 1-nitropyrene metabolites in human urine as a proposed biomarker for exposure to diesel exhaust.. Chem Res Toxicol.

[r156] U.S. EPA (U.S. Environmental Protection Agency). (2005a). Exposure Assessment Tools and Models: Source Ranking Database.. http://www.epa.gov/opptintr/exposure/pubs/srd.htm.

[r157] U.S. EPA (U.S. Environmental Protection Agency). (2005b). Inventory Update Rule: Non-confidential Production Volume Information Submitted by Companies for Chemicals Under the 1986–2002 Inventory Update Rule (IUR).. http://www.epa.gov/hpv/pubs/general/hpvcolst.htm.

[r158] U.S. EPA (U.S. Environmental Protection Agency). (2010). Dyes Derived from Benzidine and its Congeners.. http://www.epa.gov/oppt/existingchemicals/pubs/actionplans/DCBActionPlan_06232010.noheader.pdf.

[r159] U.S. EPA (U.S. Environmental Protection Agency). (2011a). Exposure Factors Handbook: 2011 Edition. Washington, DC:US. EPA.. http://www.epa.gov/ncea/efh/pdfs/efh-complete.pdf.

[r160] U.S. EPA (U.S. Environmental Protection Agency). (2011b). Toluene Diisocyanate (TDI) and Related Compounds Action Plan [RIN 2070-ZA14].. http://www.epa.gov/oppt/existingchemicals/pubs/actionplans/tdi.pdf.

[r161] U.S. EPA (U.S. Environmental Protection Agency). (2012). EPA Existing Chemicals Action Plans.. http://www.epa.gov/opptintr/existingchemicals/pubs/ecactionpln.html.

[r162] Warner M Mocarelli P Samuels S Needham L Brambilla P Eskenazi B 2011 Dioxin exposure and cancer risk in the Seveso Women’s Health Study. Environ Health Perspect 119 1700 1705; 10.1289/ehp.1103720 21810551 PMC3261987

[r163] Weisel CP, Kim H, Haltmeier P, Klotz JB (1999). Exposure estimates to disinfection by-products of chlorinated drinking water.. Environ Health Perspect.

[r164] White SS Stanko JP Kato K Calafat AM Hines EP Fenton SE 2011 Gestational and chronic low-dose PFOA exposures and mammary gland growth and differentiation in three generations of CD-1 mice. Environ Health Perspect 119 1070 1076; 10.1289/ehp.1002741 21501981 PMC3237341

[r165] Woloshin S, Schwartz LM, Welch HG (2008). The risk of death by age, sex, and smoking status in the United States: putting health risks in context.. J Natl Cancer Inst.

[r166] Xue F, Willett WC, Rosner BA, Hankinson SE, Michels KB (2011). Cigarette smoking and the incidence of breast cancer.. Arch Intern Med.

[r167] Yang XR, Chang-Claude J, Goode EL, Couch FJ, Nevanlinna H, Milne RL (2011). Associations of breast cancer risk factors with tumor subtypes: a pooled analysis from the Breast Cancer Association Consortium studies.. J Natl Cancer Inst.

[r168] Zwirner-Baier I, Neumann HG (1999). Polycyclic nitroarenes (nitro-PAHs) as biomarkers of exposure to diesel exhaust.. Mutat Res.

