# Supplementary material for: New Exposure Biomarkers as Tools for Breast Cancer Epidemiology, Biomonitoring, and Prevention: A Systematic Approach Based on Animal Evidence
Source: Environ Health Perspect. 2014 May 12;122(9):881–95. doi: 10.1289/ehp.1307455 (PMC4154213; doi:10.1289/ehp.1307455)
Supplement: (815 KB) PDF [file ehp.1307455.s001.pdf]

Environmental Health Perspectives doi:10.1289/ehp.1307455

Supplemental Material

New Exposure Biomarkers as Tools For Breast Cancer Epidemiology, Biomonitoring, and Prevention: A Systematic Approach Based on Animal Evidence

Ruthann A. Rudel, Janet M. Ackerman, Kathleen R. Attfield, and Julia Green Brody

Table of Contents **Page**

Table S1. Summary of exposure sources and biomonitoring methods for 102 rodent mammary gland carcinogens with likely human exposure **2**

Table S2. Guide to breast cancer cohort studies (studies assessing breast cancer incidence, recurrence, or survival) **25**

Table S3. Guide to breast cancer cohort studies (studies assessing pubertal development) **33**

References **36**

Table S1. Summary of exposure sources and biomonitoring methods for 102 rodent mammary gland carcinogens with likely human exposure.

These 102 were selected from the 216 rodent mammary gland carcinogens compiled by Rudel et al. (2007) based on likelihood of general population or occupational exposure, because they are produced in high volumes, over 5000 women are exposed occupationally each year, because they are present in food, air position, or consumer products, or were identified as exposure pharmaceuticals by Friedman et al. (2009).

CAS	Name	Chemical group	Exposure summary	Biomarker summary
106-99-0	1,3-Butadiene	1,3-Butadiene	The primary route of exposure is inhalation from gasoline fumes, automobile exhaust, and cigarette smoke. Over 12 billion pounds/year are produced globally, and occupational exposure occurs in many industries, especially synthetic rubber manufacturing and petroleum refining (OSHA 2012). Although some food packaging contains residual 1,3-butadiene, the available data indicate that it does not usually migrate to the food. Certain cooking oils, such as rapeseed oil (canola) release 1,3-butadiene when heated (NTP 2011). It is on the Canadian Priority Substances List, with exposures from urban ambient air and cigarette smoke (Health Canada 2000). It is listed as a Proposition 65 carcinogen and developmental toxicant (California OEHHA 2014).	Many occupational studies, epidemiological studies, and studies comparing smokers and non-smokers have measured biomarkers of exposure to 1,3-butadiene (BD) in blood and urine, and a few have measured biomarkers in exhaled breath. Widely used approaches include the measurement of DNA and hemoglobin adducts in blood (Il'yasova et al. 2009; Ogawa et al. 2006; Vacek et al. 2010), and the measurement of mercapturic acid metabolites in urine. CDC is planning to add UPLC-ES-MS/MS testing for the BD-derived mercapturic acids N-acetyl-S-(3,4-dihydroxybutyl)-L-cysteine (DHBMA; LOD 5 ng/mL), N-acetyl-S-(1-hydroxymethyl-2-propanyl)-L-cysteine (MHBMA1; LOD 0.7 ng/mL), N-acetyl-S-(2-hydroxy-3-butenyl)-L-cysteine (MHBMA2; LOD 0.7 ng/mL), and N-acetyl-S-(4-hydroxy-2-buten-1-yl)-L-cysteine (MHBMA3; LOD 0.6 ng/mL) to future NHANES reports (Alwis et al. 2012). A CDC pilot study found that DHBMA and MHBMA3 levels were significantly different in smokers and non-smokers, while MHBMA1 was not detectable in the majority of both smokers and non-smokers, and MHBMA2 was detectable in the majority of smokers but not non-smokers (Alwis et al. 2012). DNA and hemoglobin adducts of epoxide metabolites of 1,3-BD have been measured in blood (Il'yasova et al. 2009; Swenberg et al. 2011; Zhao et al. 2001). N-(2,3,4-trihydroxybutyl)valine (THB-val) is the most prevalent Hb adduct in humans. Many studies have also measured N-(2-hydroxy-3-butenyl)valine (HB-val). The hemoglobin adduct N,N-(2,3-dihydroxy-1,4-butadiyl)-valine (pyr-val) reflects exposure to 1,2:3,4-diepoxybutane (DEB), the most toxic epoxide metabolite of 1,3-butadiene, and methods to detect it in general population samples have only recently been developed (Boysen et al. 2012; Il'yasova et al. 2009). Older studies have measured adducts via Edman method GC-MS or MS/MS, but newer studies tend to use trypsin hydrolysis, immunoaffinity purification, and LC-MS/MS (Ogawa et al. 2006; Swenberg et al. 2011). A few studies have shown lower levels of Hb adducts, but generally similar levels of genetic damage, in women compared to in men after exposures to similar amounts of BD (Albertini et al. 2007; Vacek et al. 2010). Mercapturic acids, measured by various forms of LC-MS/MS (Eckert et al. 2010; Kotapati et al. 2011; Sapkota et al. 2006; Schettgen et al. 2009), are the most commonly measured urinary biomarkers of BD, though Shen et al. (2009) describe a method for measurement of the diol metabolite 3-butene-1,2-diol. Kotapati et al. (2011) measured trihydroxybutyl mercapturic acid in urine from smokers and non-smokers by HPLC-ESI-MS/MS, finding higher levels in smokers but detecting it in non-smokers as well. Eckert et al. (2011) detected dihydroxybutyl mercapturic acid (DHBMA) in all urine samples from non-occupationally exposed Germans and monohydroxy butenyl mercapturic acids (MHBMA; comprising MHBMA1, MHBMA2, and MHBMA3) in 10%, and did not find a difference in median levels between smokers and non-smokers. Carmella et al. (2009) measured MHBMA and DHBMA, along with urinary metabolites of other PAHs, in subjects who had recently stopped smoking, finding that all metabolites but DHBMA had declined significantly by three days after cessation of smoking. Arayasiri et al. (2010) found no difference in MHBMA levels between traffic policemen and office policemen in central Bangkok, and Hecht et al. (2010) found no significant differences in levels of mercapturic acid biomarkers of 1,3-butadiene between non-smoking Chinese women who cooked at home and those who did not. Sapkota et al. (2006) found MHBMA and DHBMA elevated, but nonsignificantly, in urine from toll collectors compared to other subjects, and in urine collected from people in the city on a weekday compared to urine collected from people in suburbs on weekends. Perbellini et al. (2003b) found significantly higher levels of BD in exhaled breath (LOD 0.8 ng/L), blood (LOD 0.8 ng/L), and urine (LOD 1 ng/L) from smokers than from non-smokers. Gordon et al. (2002) also measured BD in exhaled breath of smokers and non-smokers, and Smith et al. (2008) measured BD in exhaled breath during and after 20 minutes of intentional inhalation exposure. Many studies are reviewed by Wang et al. (2011), Swenberg et al. (2011), Il'yasova et al. (2009), Ogawa et al. (2006), Albertini et al. (2003), and Boogaard et al. (2002).
79-06-1	Acrylamide	Acrylamide	The general public may be exposed through consumption of certain foods (e.g. french fries). Acrylamide is formed during heating of starch-rich foods to high temperatures. Other sources of exposure could include drinking water contaminated with polyacrylamide flocculants used in water treatment and contact with polyacrylamide-containing consumer products. Tobacco smoke is a substantial non-food source of exposure to acrylamide (NLM 2011). It is used in the manufacture of consumer products, including textiles, contact lenses, building materials, cosmetic and soap preparations, water-based paints, home appliances, automotive parts, food packaging adhesives, paper, gelatin capsules, and in home-use adhesives and caulks (NLM 2013). It is an environmental contaminant (NTP 2011). It is listed as a Proposition 65 carcinogen (California OEHHA 2014). It is on the REACH SVHC Candidate List, with exposures from drinking water, cosmetics, and soil conditioners for the garden (ECHA 2013).	NHANES and others have used biomarkers in blood and urine to assess exposure to acrylamide in occupational settings and in the general population, while at least one study has measured acrylamide in breast milk and human placenta, and a method has been attempted in rats for measuring acrylamide exposure in cerebral spinal fluid. NHANES has used Edman reaction followed by HPLC-MS/MS to measure N-terminal hemoglobin adducts of acrylamide and its metabolite glycidamide in blood from over 7000 subjects in 2003-2004 (CDC 2008a, 2009; Vesper et al. 2010). Both adducts were found in most subjects, at higher levels in children than in adults (Vesper et al. 2010), and there was a small but significant correlation between dietary acrylamide intake (assessed by food frequency questionnaire) and adduct levels (Tran et al. 2010). CDC is planning to add UPLC-ES-MS/MS testing for the AA and GA-derived urinary mercapturic acids N-acetyl-S-(2-carbamoylethyl)-L-cysteine (AAMA; LOD 2.2 ng/mL) and N-acetyl-S-(2-carbamoyl-2-hydroxyethyl)-L-cysteine (GAMA; LOD 9.4 ng/mL) to future NHANES reports (Alwis et al. 2012). Others have used similar methods to NHANES, GC-NCI-MS/MS (T. Schettgen et al. 2010), SPE-LC-ESI-MS/MS (Bjellaas et al. 2007a), or immunoassays (Preston et al. 2009) to measure HbAA and HbGA. Levels are generally higher in smokers than non-smokers (Bjellaas et al. 2007a; Bjellaas et al. 2007b; Hagmar et al. 2005; von Stedingk et al. 2010), but apparently not higher in people exposed to secondhand smoke (T. Schettgen et al. 2010). Adduct levels are higher in maternal blood than corresponding cord blood samples (Schettgen et al. 2004; von Stedingk et al. 2011). Many more studies have used LC-MS/MS to measure AAMA, GAMA, N-acetyl-S-(1-carbamoyl-2-hydroxyethyl)-cysteine (GAMA2), and N-acetyl-S-(propionamide)-cysteine (NAPC) in urine in samples from exposed workers and controls (Huang et al. 2007; Huang et al. 2011a; Huang et al. 2011b; Kopp et al. 2008), and in samples from general population groups including pregnant women and children as young as five (Brantsaeter et al. 2008; Hartmann et al. 2008; Heudorf et al. 2009; C-M Li et al. 2005). Levels of acrylamide-derived mercapturic acids have been found to be higher in exposed workers and in smokers (Alwis et al. 2012; Huang et al. 2011a; Huang et al. 2011b; CM Li et al. 2005). At least one study has measured acrylamide in breast milk and placenta (Sörgel et al. 2002). Additional studies using biomarkers of acrylamide exposure are reviewed in Il'yasova et al. (2009), Dybing et al. (2005), Knudsen et al. (2007), and Ogawa et al. (2006).

CAS	Name	Chemical group	Exposure summary	Biomarker summary
39156-41-7	2,4-Diaminoanisole sulfate	Aromatic amine	2,4-diaminoanisole sulfate is an aromatic amine which was used extensively in hair dyes and in the dyeing of furs until the late 1970s (IARC 2001a; NTP 2011). The maximum concentration of the compound in hair-dye preparations was approximately 1.5% (NTP 2011). It is listed as a Proposition 65 carcinogen (California OEHHA 2014).	No studies were found using biomarkers to measure exposure to 2,4-diaminoanisole sulfate.
95-80-7	2,4-Diaminotoluene	Aromatic amine	TDA is a metabolite of toluene diisocyanate (TDI), and exposure can occur from use of products containing uncured TDI and its related polyisocyanates, such as spray-applied sealants and coatings, or when such products are used in or around buildings including homes or schools. Potential consumer exposure may occur as a result of the presence of trace contaminants in products that contain TDA-based dyes, such as furs, leather, silk, textiles, and wool, and a number of hair dyes contained this chemical prior to the 1970s. TDA is used in the production of polyurethane foam and sealants, and it has been identified as a degradation product of polyester urethane foam used to cover silicone breast implants. Relatively high levels were detected in plasma and urine of patients one month after surgery, and measurable levels were detected in patients up to two years after surgery. Small amounts of TDA are released from boil-in bags upon prolonged boiling (NTP 2011). It is listed as a Proposition 65 carcinogen (California OEHHA 2014). It is listed on the REACH SVHC Candidate List, with exposures from TDI production and explosives manufacturing (ECHA 2013). TDI is a US EPA Action Plan Chemical, with occupational exposures and exposures from consumer products, such as sealants used in building materials (US EPA 2013a).	Many studies have investigated 2,4-diaminotoluene levels in blood and urine of occupationally exposed workers, as well as nonexposed control subjects and women with breast implants containing polyurethane. 2,4-diaminotoluene (also known as 2,4-toluene diamine or 2,4-TDA) is a metabolite and degradation product of 2,4-toluene diisocyanate (2,4-TDI), and is often used as a biomarker of exposure to 2,4-TDI. Hester et al. (1997) used GC-MS, with LLOQ 10 pg/ml on urine and serum samples from 61 patients with breast implants and 21 women without. This study found no detectable 2,4-TDA in serum of either group. Of the women with breast implants, 30 had quantifiable levels of 2, 4-TDA in their urine, and 18 had detectable but not quantifiable levels. Of the controls, 7 had detectable levels in urine but none had quantifiable levels. Dalene et al. (1990) used GC-MS to measure 2,4-TDA in blood and urine from 15 factory workers exposed to TDI-based foam, also taking samples from 4 of the workers during an exposure-free period. Plasma levels ranged from below 0.1 to 5.5 ng/ml, and variation was greater between workers than between samples from any given worker. Sennbro et al. (2004) measured plasma, urine, and air for 81 exposed workers and 121 unexposed workers, finding strong associations between the levels in both media and results of personal air sampling. Jones et al. (2005) measured hemoglobin adducts in Chinese workers exposed to nitrotoluenes, finding 4-amino-2-nitrotoluene 24 times more abundant than 2,4-TDA in workers exposed to 2,4-TDI. Rosenberg et al. (2002) used GC-MS with LLOQ 0.25 nmol/l on urine from workers in 5 polyurethane-processing environments. The highest measured TDA concentration (including 2,4- and 2,6-TDA) was 0.79 nmol/mmol creatinine, while the mean in unexposed workers was 0.08 nmol/mmol creatinine. Maitre et al. (1993) found urinary TDA concentrations ranging from 6.5 to 31.7 µg/g creatinine in postshift samples from 9 workers in TDI-based polyurethane production. These concentrations were linearly related to atmospheric concentrations of TDI. See also methods for TDI.
613-13-8	2-Aminoanthracene	Aromatic amine	2-aminoanthracene is formed from the incomplete combustion of organic materials, is a constituent of synthetic fuel, and is a research chemical (NLM 2011).	No studies were found using biomarkers of exposure to 2-aminoanthracene.
91-94-1	3,3'-Dichlorobenzidine	Aromatic amine	For the general population the chance of exposure to 3,3'-dichlorobenzidine (DCB) and its dichloride salt is probably insignificant; the greatest chance of exposure is from the improper land disposal of DCB. In the past, exposure may have occurred during the use of pressurized spray containers of paints, lacquers, and enamels containing traces of benzidine yellow, an azo dye derived from DCB. The use of DCB to synthesize dyes ended in 1986, although it is still used to produce pigments (NTP 2011). It is on the Canadian Priority Substances List, with occupational exposures from production of pigments for printing inks, textiles, paints, plastics and crayons (Health Canada 2007b). It is listed as a Proposition 65 carcinogen (California OEHHA 2014). It is an EPA Action Plan Chemical, under the class of benzidine dyes (CDC 2012a).	Multiple studies have measured 3,3'-dichlorobenzidine (DCB) in the urine of occupationally exposed humans, and one study has developed a method to measure it in the blood of rats. Guerbet et al. (2007) used GC-MS on urine, detecting DCB levels of 1.62-8.9 ppb in 4 of 47 samples taken from workers in a chemical plant either after a one-month vacation or after 4 months of regular work. Hatfield et al. (1982), using GC-MS with LOD 0.2 ppb, did not detect DCB or monoacetyl-DCB in urine samples from 36 exposed workers or from 12 controls, although a nonspecific colorimetric test for aromatic amines, with LOD 1 ppb, was positive for 6 of the exposed workers and 1 of the controls. Lee and Shin (2002) developed GC-MS-SIM, with LOD 0.5 µg/L for DNA adducts and 1 ng/g hemoglobin for hemoglobin adducts in dosed rats.

CAS	Name	Chemical group	Exposure summary	Biomarker summary
119-90-4	3,3'-Dimethoxybenzidine (o-dianisidine)	Aromatic amine	Exposure could occur from trace contaminants in products that are made with 3,3'-dimethoxybenzidine. It is used as a dye for paper, plastics, rubber, and textiles (NTP 2011). It is listed as a Proposition 65 carcinogen (California OEHHA 2014). It is an EPA Action Plan Chemical, under the class of benzidine dyes (CDC 2012a).	A few relatively old studies have measured 3,3'-dimethoxybenzidine in human urine, and a few have investigated other methods to measure biomarkers of exposure. Lowry et al. (1980) used a colorimetric screening method to detect trace 3,3'-dimethoxybenzidine in urine from occupationally exposed workers. Bowman et al. (1976) used a spectrophotoflourometric method to measure 3,3'-dimethoxy benzidine in human urine and rat blood. Birner et al. (1990) measured hemoglobin adducts in rats, and Rodgers et al. (1983) measured 3,3'-dimethoxybenzidine and a few metabolites in rat urine. Methods for measuring exposure to benzidine might be adaptable to measure exposure to 3,3'-dimethoxybenzidine.
119-93-7	3,3'-Dimethylbenzidine	Aromatic amine	Swimming pool water test kits contain 0.5% to 1.0% 3,3′-dimethylbenzidine. Exposure may occur if the test solutions are emptied into the pool. Residual levels of 3,3′-dimethylbenzidine may be present in dimethylbenzidine-based dyes and pigments and in the final consumer products (IARC 1993c; NTP 2005).	A few relatively old studies have measured 3,3'-dimethylbenzidine in human urine, and a few have investigated other methods to measure biomarkers of exposure. Lowry et al. (1980) used the colorimetric screening method to detect trace 3,3'-dimethylbenzidine in urine from occupationally exposed workers. Bowman et al. (1976) used a spectrophotoflourometric method to measure 3,3'-dimethylbenzidine in human urine and rat blood. Birner et al. (1990) measured hemoglobin adducts in rats, and Rodgers et al. (1983) measured 3,3'-dimethylbenzidine and a few metabolites in rat urine. Methods for measuring exposure to benzidine might be adaptable to measure exposure to 3,3'-dimethylbenzidine.
101-14-4	4,4'-Methylene-bis(2-chloroaniline)	Aromatic amine	The general population can be exposed to MOCA in contaminated areas or upon consumption of certain types of plants grown in MOCA-contaminated soil (IARC 1993c). It is used as a curing agent for roofing and wood sealing in Japan and Asia (IARC 1993c). CPSC reported that residual levels may be present in final products, such as polyurethane foam and other plastic components. However, data describing actual levels of impurities and the potential for consumer exposure are lacking (IARC 1993c; NTP 2005). It is a TSCA Work Plan Chemical, identified as low likelihood of exposure. Relatively small releases to the environment have been reported (US EPA 2012). It is listed as a Proposition 65 carcinogen (California OEHHA 2014). It is on the REACH SVHC Candidate List, with occupational exposure from dermal contact, and little information about consumer exposure (ECHA 2013).	A few studies have measured MOCA or its metabolites in the urine of occupationally exposed subjects, and methods have been developed to measure exposure to MOCA in blood. NIOSH uses GC-ECD (NIOSH 1994b), to measure MOCA in urine with LOD 1 μg/L and LLOQ 10 μg/L given initial samples of 50 to 100 mL. Among others, Cocker et al. (2009), Keen et al. 2011 (2011), Shih et al. (2007), and Murray and Edwards (1999) measured MOCA and a few metabolites in the urine of exposed workers, with detection frequencies varying between studies from 51% to 100%. Shih et al. (2007), using SPE-LC-MS/MS, reports LODs for MOCA and a metabolite at ~200-400x below mean levels in urine from exposed workers, and ~40-60x below medians. Murray and Edwards (1999) detected MOCA in the urine of each of 12 exposed workers, and not in the urine of 18 control subjects. Keen et al. (2011) found no correlation between isocyanates and MOCA in urine. Vaughn and Kenyon (1996) developed a GC-MS method to test MOCA and its protein adducts and conjugates in blood, with LOD "well below the levels found for occupationally exposed individuals", and detected MOCA in the blood of all 5 exposed workers tested.
92-67-1	4-Aminobiphenyl	Aromatic amine	The potential for exposure to 4-aminobiphenyl is low because it has no current commercial uses. It formerly was used as a rubber antioxidant, as a dye intermediate, and in the drug and cosmetic color additive D&C yellow no. 1, which was discontinued in the 1970s. Mainstream cigarette smoke was reported to contain 4-aminobiphenyl at levels of 2.4 to 4.6 ng per cigarette (unfiltered) and 0.2 to 23 ng per cigarette (filtered), and sidestream smoke to contain up to 140 ng per cigarette (NTP 2011). It is listed as a Proposition 65 carcinogen (California OEHHA 2014). It is on the REACH SVHC Candidate List (ECHA 2013).	Many general population and occupational studies have used biomarkers of exposure to 4-aminobiphenyl (4-ABP) in blood and urine. Breast milk, bladder tissue and saliva have also been used. The most common biomarkers are adducts of 4-ABP and hemoglobin (4-ADP-Hb) measured in blood, and 4-ABP measured in urine. Many studies, using either GC-MS (often with NCI) or HPLC-MS/MS, have found higher levels of 4-ABP-Hb in smokers than in non-smokers, and higher levels among heavier smokers compared to lighter smokers (Dallinga et al. 1998; Hammond et al. 1993; Mendes et al. 2009; Myers et al. 1996; Roethig et al. 2009; Sarkar et al. 2006; Seyler and Bernert 2011). 4-ABP-Hb levels tend to be higher in maternal blood than in corresponding cord blood samples (Myers et al. 1996), and among smokers they were higher in older people compared to younger, males compared to females, and white people compared to black people (Mendes et al. 2009). Peluso et al. (2008) found small but significant inverse relationships between fiber intake, fruit intake, and BMI and 4-ABP-Hb levels. Others have investigated associations between 4-ABP-Hb and bladder cancer or other diseases, often finding higher adduct levels in cases than in controls (Airoldi et al. 2005; Del Santo et al. 1991; Skipper et al. 2003). Richter et al. (2001) found higher levels of 4-ABP-Hb in children living in bigger cities than those in smaller cities. A study in dye factories in India found much higher levels of 4-ABP-Hb in workers exposed to benzene as opposed to those exposed to dyes (Beyerbach et al. 2006), while a study in rubber factory found no difference in 4-ABP-Hb levels between exposed and unexposed workers (Ward et al. 1996). Multiple studies, mostly using GC-MS, have found higher levels of 4-ABP in the urine of smokers compared to non-smokers (Riedel et al. 2006; Seyler and Bernert 2011), although at least one study (Grimmer et al. 2000) found no such difference. A recent meta-analysis (Van Hemelrijck et al. 2009) found an association between urinary or blood 4-ABP and secondhand smoke exposure but not bladder cancer. Schettgen et al. (2010) couldn't detect 4-ABP in urine of two subjects after they had applied hair dye, but Ambrosone et al. (2007) found ABP-DNA adducts in epithelial cell DNA isolated from human breast milk in women using hair dyes. Bessette et al. (2010) used LC-ESI/MS/MS to measure 4-ABP-derived DNA adducts in saliva, but adducts were only detectable in the saliva of two smokers out of 37 volunteers. Zayas et al. (2007) used LC-MS/MS to measure DNA adducts in bladder tissue from 27 bladder cancer patients, detecting DNA adducts in samples from 12. Zayas et al. (2007) found no correlation between levels of 4-ABP-Hb and DNA adducts in bladder tissue. Gu et al. (2012) attempted to measure 4-ABP derived DNA adducts in 70 tumor-adjacent mammary tissue samples, but did not detect any. Birner et al. (1990) found that 4-ABP-Hb adducts are formed in benzidene-treated rats.
99-59-2	5-Nitro-ortho-anisidine	Aromatic amine	5-nitro-ortho-anisidine is used as a chemical intermediate in the production of C.I. Pigment Red 23 which is used as a colorant in a wide variety of commodities including printing inks, interior latex paints, lacquers, rubber, plastics, floor coverings, paper coatings, and textiles (NLM 2011).	No studies were found using biomarkers of exposure to 5-nitro-ortho-anisidine.

CAS	Name	Chemical group	Exposure summary	Biomarker summary
92-87-5	Benzidine	Aromatic amine	Uses of benzidene and some related chemicals have decreased, particularly in the US and Europe, because they are known to cause bladder cancer in humans (NTP 2011). However many benzidine-based dyes are still produced and used in significant quantities in the US and elsewhere (US EPA 2010a). Benzidine-based and related dyes are used in the production of textiles, paints, printing inks, paper, and pharmaceuticals; as reagents and biological stains in laboratories; in the food industries; and in laser, liquid crystal displays, ink-jet printers, and electro-optical devices (US EPA 2010a). Some dyes used to color paper, cloth, leather, food and drinks may contain benzidine as a contaminant or other impurities that can be broken down into benzidine once inside the body (IARC 2010; NLM 2011). It is on the Canadian Priority Substances List, with limited environmental exposures (Health Canada 2007c). Proposition 65 carcinogen (California OEHHA 2014). It is an EPA Action Plan Chemical, under the class of benzidine dyes (US EPA 2010a).	Benzidine biomarkers can indicate exposure to benzidine and to benzidine-based azo dyes. Occupational studies have detected parent, metabolite, and adducted forms of benzidine in blood and urine, but more sensitive methods are in development in animal studies. Hemoglobin adducts have been detected in 33 exposed workers with GC-MS (Beyerbach et al. 2006). Older human occupational studies used 32P postlabeling on white blood cells, urine, and sputum in exposed workers and controls. Parent benzidine and two metabolites (N-acetylbenzidine and N,N'-diacetylbenzidine) were detected in worker's urine with LODs in ppt via GC-MS (Hsu et al. 1996). Two NIOSH methods detect benzidine in urine via visible absorption/TLC extraction with estimated LOD 0.1 µg/dL urine (NIOSH 1993) and via GC-ECD, estimated LOD 5 µg/L, LLOQ 10 µg/L (NIOSH 1994a). Animal studies have measured DNA adducts with HPLC-MS/MS with LOD 22 pg on column (Means et al. 2003) and Birner et al. (1990) identified several hemoglobin adducts including benzidine metabolite 4-aminobiphenyl. A more sensitive method has been developed on treated animal blood and tissue samples using supercritical fluid chromatography with LLOQ 0.10 ng/mL (Patel and Agrawal 2003).
6459-94-5	C.I. Acid Red 114	Aromatic amine	The general population may be exposed via dermal contact by direct dyeing of wool and silk using consumer products containing the compound (NLM 2004). CI Acid Red 114 is used to dye wool, silk, jute, and leather. It is metabolized to 3,3'-dimethylbenzidine (NTP 2011). It is listed as a Proposition 65 carcinogen (California OEHHA 2014).	No studies were found using biomarkers to measure exposure to CI Acid Red 114
569-61-9	C.I. Basic Red 9 monohydrochloride	Aromatic amine	Consumer exposure could possibly occur through contact with products containing residual dye (NTP 2011). CI Basic Red 9 monohydrochloride is used to dye textile fibers, in the preparation of pigments for printing inks, and in other specialty applications (IARC 1993d). It is one of three components of commercial magenta which is used as a dye for coloring textiles (cotton, wool, silks, and acrylics), china clay products, leather, printing inks, and as a filter dye in photography. Its specialty applications include tinting automobile antifreeze solutions and toilet sanitary preparations (NTP 2011). It is listed as a Proposition 65 carcinogen (California OEHHA 2014).	No studies were found using biomarkers to measure exposure to CI Basic Red 9 monohydrochloride
1937-37-7	C.I. Direct Black 38	Aromatic amine	C.I. Direct Black 38 and other azo dyes are used on textiles such as cotton, silk, wool, nylon, acetate and leather, and used in aqueous printing inks and as biological stains, plastics, wood stains, wood flour, and hair dyes (NTP 1978b). It is listed as a Proposition 65 carcinogen (California OEHHA 2014).	No studies were found using biomarkers specifically to measure exposure to C.I. Direct Black 38. Metabolites include benzidine, 4-aminobiphenyl, monoacetylbenzidine, and acetylaminobiphenyl; biomarker methods for benzidine could be used to assess exposure to C.I. Direct Black 38.

CAS	Name	Chemical group	Exposure summary	Biomarker summary
636-21-5	Ortho-toluidine hydrochloride	Aromatic amine	The general population may be exposed to low concentrations of o-toluidine in ambient air, tobacco smoke, food, or dermal contact with commercial products (NTP 2011). Exposure has also been reported during its use in production of dyestuffs and rubber chemicals (IARC 2000a). It is listed as a Proposition 65 carcinogen (California OEHHA 2014).	Many studies have measured o-toluidine or its adducts in the blood and urine of the general population, smokers, patients receiving certain drugs, and exposed workers, and one study has measured o-toluidine in exhaled breath. Gaber et al. (2007) measured hemoglobin adducts of o-toluidine by GC-MS in blood collected from 10 surgical patients and 6 healthy volunteers before and 24 hours after receiving the anesthetic prilocaine, and found 6-360-fold increases, from a baseline mean of 0.54 ng/g hemoglobin to a mean of 22 ng/g 24 hours after treatment, excluding one patient with very high initial levels (40.9 ng/g before, 64.4 ng/g after). Smoking status did not affect background or posttreatment levels. Kutting et al. (2009) found o-toluidine levels were significantly higher in the urine of smokers, with o-toluidine above the LLOQ in urine samples from 178 of 1004 Bavarian subjects. Riedel (2006), using GC-MS with negative ion chemical ionization, detected o-toluidine above the LOD of 4 ng/mL in 10 urine samples from non-smokers and 10 urine samples from smokers; smokers had higher levels. Labat et al. (2006) used GC-MS with negative chemical ionization, with LOD 0.02 µg/L, to measure o-toluidine in 5 ml urine samples from workers involved in the demolition of an old chemical plant. Levels in samples from unexposed controls ranged from 0.17 to 2.46 µg/g creatinine, while levels in samples from exposed workers ranged from 26.17-462 µg/g, but went down to 2.35-20.11 ng/g after the introduction of new protective measures. Rieder et al. (2001) used proton transfer mass spectroscopy for the measurement of VOCs including o-toluidine (which they describe as endogenously produced) in exhaled breath.
26471-62-5	Toluene diisocyanate mixtures	Aromatic amine	Because of the high volatility of toluene diisocyanates, exposure can occur in all phases of its manufacture and use. Exposure can occur from use of products containing uncured TDI and related polyisocyanates, such as spray foam insulation and spray-applied sealants and coating, and incidental exposures to the general population while such products are used in or around buildings including homes or schools (US EPA 2011) Household products employing polyurethane varnishes or foam such as furniture, carpet underlay, and bedding may volatize unreacted toluene diisocyanates. FDA has determined that levels of toluene diisocyanates in food, food additives, or food packaging are very low (NTP 2011). It is an EPA Action Plan Chemical, with exposures from building materials and some hobby products (US EPA 2013a). It is listed as a Proposition 65 carcinogen (California OEHHA 2014).	Biomarkers in blood and urine have been widely used to measure exposure to toluene diisocyanate in occupational and exposed-volunteer studies. The diamine derivative of TDI, toluene diamine (2,4-diaminotoluene) and the diamine derivatives of other diisocyanates can be detected after lysis from protein adducts in urine or blood through gas chromatography-mass spectrometry, with LODs around 1 nmol per liter in urine (about 0.1 micromole diamine per mole creatinine) (Cocker 2011). Measurements of isocyanate derived diamines in blood and urine have been well correlated with TDI measurements in air in volunteer and occupational studies, but these measurements may reflect exposure to toluene diamine as well as toluene diisocyanate (Cocker 2011). Studies have also examined levels of TDI-albumin adducts and TDI-specific antibodies in serum (Brown and Burkert 2002). See reviews by Cocker et al. (2011) and Brown and Burkert (2002). See also methods for 2,4-diaminotoluene.
71-43-2	Benzene	Benzene	The primary sources of exposure to benzene for the general population are ambient air containing tobacco smoke, air contaminated with benzene, drinking contaminated water, or eating contaminated food (IARC 1982). Exposure to benzene is highest in areas of heavy motor vehicle traffic and around gasoline filling stations. Consumer products containing benzene include carpet, pesticide products, adhesive removers, and home-use paints, sealants, finishers, and auto oils (NLM 2013; NTP 2011). Major contributors to benzene emissions into air include: (1) gasoline production, storage, transport, vending and combustion; (2) production of other chemicals from benzene; and (3) indirect production of benzene (coke ovens), which is a major source of benzene emissions into water (IARC 1982). Benzene is a TSCA Work Plan Chemical, identified as having a high likelihood of exposure. It is present in biomonitoring, drinking water, indoor environments, and soil, and high releases to the environment have been reported (US EPA 2012). It is on the Canadian Priority Substances List, with exposures from ambient air, cigarette smoke, and to a lesser extent, food and consumer products (Health Canada 2007a) It is listed as a Proposition 65 carcinogen (California OEHHA 2014).	NHANES and others have measured benzene in blood samples taken from the general population, generally detecting benzene in all or most samples. Other researchers have measured unmetabolized benzene in urine and breath, benzene metabolites in urine, and adducts to proteins and DNA in blood and dried blood spots. NHANES detected benzene in over half the population via HS-SPME-GC-MS on 3 mL (minimum) to 10 mL (optimal) whole blood, with LOD 0.024 ng/mL (Blount et al. 2006; CDC 2008b, 2009). CDC is planning to add UPLC-ES-MS/MS testing for the urinary benzene metabolites trans trans muconic acid (ttMA; LOD 12 ng/mL) and s-phenyl mercapturic acid (sPMA; LOD 0.3 ng/mL) to future NHANES reports (Alwis et al. 2012). A CDC pilot study found that ttMA and sPMA levels were significantly higher in smokers than in non-smokers (Alwis et al. 2012). Unmetabolized benzene can be measured in blood, urine, and breath samples from the general population by purge and trap, headspace, SPE, or SPME extraction followed by GC-MS, with LODs in pg/mL (Weisel 2010), but it has a half-life of only minutes to hours, and samples can easily become contaminated with benzene from the environment (Johnson et al. 2007). Nonetheless, benzene levels in blood can differentiate between exposed and unexposed workers, smokers and non-smokers, and pre-and post-shift samples, and urinary benzene levels were associated with workspace air benzene concentrations in at least one study (Weisel 2010). Hemoglobin, albumin, and DNA adducts have also been measured, with much longer half-lives (2 to 3 weeks for albumin adducts, about 4 months for hemoglobin adducts, and longer for DNA) (Johnson et al. 2007; Weisel 2010). Funk et al. (2008) demonstrated that benzene oxide-hemoglobin adducts could be measured in dried blood spots by GC-MS. Urinary metabolites ttMA and sPMA have been measured by SPE or SPME followed by HPLC-UV, HPLC-MS/MS, or GC-MS, typically with LODs between 5-10 µg/L (Weisel 2010). Both are generally well correlated with benzene exposures from 0.1-20 ppm, but ttMA is also a metabolite of the food additive sorbic acid, whereas sPMA has no known sources beside benzene (Weisel 2010). Researchers have also measured the urinary benzene metabolites phenol, catechol, and hydroquinone via liquid extraction, SPE, or SPME followed by GC FID, GC-MS, HPLC UV, or HPLC-MS/MS (Weisel 2010).

CAS	Name	Chemical group	Exposure summary	Biomarker summary
75-21-8	Ethylene oxide	Ethylene oxide	The general population may be exposed to ethylene oxide (EtO) through use of products that have been sterilized with the compound, such as medical products, foods, clothing, cosmetics, beekeeping equipment, and other products. EtO has been detected in tobacco smoke, automobile exhausts, and in some foods and spices (NTP 2011). It is found in household rust neutralizer, driveway cleaner, and transmission fluid (NLM 2013). It is on the Canadian Priority Substances List, with exposures from indoor air, food, spices, and medical equipment sterilized with EtO (Environment Canada 2011). It is listed as a Proposition 65 carcinogen (California OEHHA 2014).	Methods for detecting urinary metabolites, DNA adducts, and hemoglobin adducts of ethylene oxide (EtO) have been employed in occupational and general population studies. NHANES has measured 2-hydroxyethyl mercapturic acid (HEMA), a common metabolite of 1,2-dibromoethane, vinyl chloride, acrylonitrile, and EtO, in urine by isotope dilution and HPLC-MS/MS, detecting it in 71% of samples, with higher levels in smokers (Calafat et al. 1999). CDC is planning to add UPLC-ES-MS/MS testing for urinary HEMA (LOD 0.6 ng/mL) to future NHANES reports (Alwis et al. 2012). CDC currently measures acrylamide- and glycidimide-derived hemoglobin adducts in blood and plans to add EtO adducts to the method (CDC 2008a). Tomkins et al. (2008) measured five ethylene oxide-derived hydroxyethyl DNA adducts via HPLC-LC-MS/MS (SRM), with LODs 0.5-25 fmol. Yong et al. (2007) detected DNA adducts in granulocytes in blood from hospital workers using HPLC-GC-EC-MS. The lowest concentration of adducts was 1.6 per 10^7 nucleotides. They note that adducts in lymphocytes would be more informative for longer term (up to one year) exposures. Huang et al. (2008) looked for the same adduct (N7 (2'-hydroxyethyl)guanine) in urine of nonsmokers, and detected adducts in 40/46 samples with LOD 0.25 ng/mL using LC-MS/MS. Yong et al. (2007) warns that since DNA adducts are thought to have a short half-life, hemoglobin adducts may be more useful for understanding long term exposures. von Stedingk et al. (2010) developed a method for detecting hemoglobin adducts by LC-MS/MS with LLOQ 1pmol adduct/g Hb. A GC-EI-MS method has LOD 1.8 pmol/g in 0.1 g hemoglobin and LLOQ 12 pmol/g in human blood samples (Ahn and Shin 2006). The 95th percentile for N-2-hydroxyethylvaline (HEV) was 1280 pmol/g globin (=29.4 microg/l blood) in blood from exposed workers compared with 100 pmol/g globin (or 2.3 microg/l) in controls (Schettgen et al. 2002). HEMA was measured in urine from non-smokers (median 2 µg/L) and smokers (median 5.3 µg/L) via HPLC-MS/MS with LOD 0.5 µg/L (Schettgen et al. 2008).
75-56-9	Propylene oxide	Propylene oxide	General population exposure may occur through ingestion of propylene oxide residues in foods from its use as an indirect registered food additive (gas sterilant) (US FDA 2013) and tobacco smoke. Exposure may also occur by contact with consumer products containing the chemical, especially automotive and paint products which have been found to contain high concentrations of PO. It is also used to manufacture polyurethane foam (NTP 2011). It is listed as a Proposition 65 carcinogen (California OEHHA 2014). It is on the REACH SVHC Candidate List, with low exposures from the environment and consumer products, such as brake fluid (ECHA 2013).	CDC has developed a method to measure PO exposure in urine from the general population. A number of studies have used biomarkers in blood and urine to investigate exposure to PO in occupationally- or tobacco-exposed humans and controls with no known exposure, and one study measured PO in the exhaled breath of humans intentionally exposed to propylene. CDC is planning to add UPLC-ES-MS/MS testing for the PO-derived mercapturic acid urinary metabolite N-acetyl-S-(3-hydroxy propyl-1-methyl)-L-cysteine (2HPMA; LOD 1.3 ng/mL) to future NHANES reports (Alwis et al. 2012). A CDC pilot study found that urine 2HPMA levels were significantly different in smokers and non-smokers (Alwis et al. 2012). The NHANES method for measuring acrylamide-derived hemoglobin adducts in blood was originally developed to measure EtO, PO and styrene oxide adducts (CDC 2008a). Schettgen et al. (2010) measured the n-terminal hemoglobin adduct N-(R,S)-2-hydroxypropylvaline (HPVal) in blood from 104 non-smokers via gas chromatography after Edman degradation and acetonization, with LOD OF 0.5 pmol/g globin, and found similar levels (median ~4 pmol/g) in those exposed to passive cigarette smoke and the unexposed. Shin et al. (2006) used Edman degradation and ethyl ether extraction, followed by GC-MS, with LOD 10 pmol/g Hb to measure HPVal ranging from below the LOD to 1100 pmol/g Hb. Czene et al. (2002) measured 1-2-hydroxypropyladenine DNA adducts in blood by (32)P-postlabeling and HPVal by GC-MS/MS in 8 exposed workers and 8 controls. DNA adducts were present in blood from 7 of the 8 exposed workers and none of the controls. HPVal was detected in all subjects, with much higher levels in workers (mean 2.7 pmol/mg globin) than in controls (mean 0.006 pmol/mg). DNA adducts, hemoglobin adducts, and sister chromatid exchanges were all correlated. Jones et al. (2005) and Ball et al. (2005) used ELISA in whole blood to measure HPVal, both with LLOQ 2 pmol/g globin. Jones (2005) reports that most of 800 samples collected over a two-year period from workers at 3 European manufacturing sites contained less than 50 pmol HPVal/g globin. Schettgen et al. (2002) measured HPVAL in blood from exposed textile workers and controls, but all were below the LOD of 80 pmol/g HB. Schettgen et al. (2008) used HPLC-MS/MS, with LOD 5 µg/L, to measure N-acetyl-S-2-hydroxypropyl-cysteine (2-HPMA), a mercapturic acid metabolite of propylene oxide, in the urine of 14 smokers (median 41.7 µg/L) and 14 non-smokers (median 7.1 µg/L). Filser et al. (2008) measured PO in exhaled breath by GC-MSD after subjects were exposed to 9-24 ppm propylene for 180 minutes.
3296-90-0	2,2-Bis(bromomethyl)-1,3-propanediol	Flame retardant	The primary routes of exposure to bis(bromomethyl)-1,3-propanediol are inhalation and dermal contact. It is a flame retardant used in polyester resins and polyurethane foams and may enter the environment as dust and through wastewater. It is expected to be persistent in water (IARC 2000c; NTP 2011). It is listed as a Proposition 65 carcinogen (California OEHHA 2014).	No studies were found using biomarkers to measure exposure to bis(bromomethyl)-1,3-propanediol in humans. A pharmacokinetic study in rats found that the main route of excretion was as a gluconoride metabolite in urine (Hoehle et al. 2009), but humans are much slower than rats at producing the glucuronide metabolite. Possibly this compound could be measured using a GC-MS method that also can measure 2,3-dibromopropanol (De Alwis et al. 2007) or an LC-MS/MS method for bis(1,3-dichloro-2-propyl) phosphate (BDCPP), a metabolite of a chlorinated tris organophosphate flame retardant (Cooper et al. 2011).
96-13-9	2,3-Dibromo-1-propanol	Flame retardant	The primary routes of exposure are inhalation and dermal contact. DBP is a metabolite and degradation product of tris(2,3-dibromopropyl) phosphate, a flame retardant that was used in children's sleepwear in the 1970s. DBP was detected in urine of children wearing sleepwear treated with Tris (NTP 2011). DBP is also a potential metabolite, impurity, and degradation product of a newer flame retardant, tetrabromobisphenol A bis(2,3-dibromopropyl ether), which is an HPV chemical that has been proposed for carcinogenicity testing at NTP (Haneke 2002). DBP is a listed Proposition 65 carcinogen (California OEHHA 2014).	2,3-dibromo-1-propanol (DBP) has been measured in urine. De Alwis et al. (2007) used SPE-GC-MS on spiked human urine, reporting 96% recovery and LOD 0.1 ng/ml. Blum et al. (1978) detected DBP in urine of children wearing pajamas treated with the flame retardant tris(2,3-dibromopropyl) phosphate. Based on studies with the chlorinated analog of this flame retardant, tris(dichloropropyl)phosphate, the bis-metabolite may be more stable and easier to detect in urine via LC-MS/MS (Cooper et al. 2011).

CAS	Name	Chemical group	Exposure summary	Biomarker summary
75-34-3	1,1-Dichloroethane	Halogenated organic solvent	The general population may be exposed via inhalation (for those people living near source areas), ingestion of contaminated drinking water, and use of consumer products, such as paint removers, that may contain this compound (NLM 2011). This chemical is a TSCA 2013/2014 Work Plan Chemical, identified as having a high likelihood of exposure. It is used in consumer products and present in drinking water, surface water, ambient air, groundwater, soil, and biomonitoring. Moderate releases to the environment have been reported (US EPA 2012). It is listed as a Proposition 65 carcinogen (California OEHHA 2014).	NHANES has used HS-SPME-GC-MS on 3 mL (minimum) to 10 mL (optimal) whole blood, but less than 5% of the population had levels above the LOD of 0.01 ng/mL (Blount et al. 2006; CDC 2008b, 2009). Possibly a method to measure non-specific urine metabolites such as haloacetic acids and haloalcohols could be developed.
96-18-4	1,2,3-Trichloropropane	Halogenated organic solvent	The general population may be exposed by ingestion of contaminated well water or by inhalation of contaminated air (NTP 2011). Detected in water, including drinking-water, and in soil as a result of its presence as an impurity in a commercial nematocide. Formerly produced as paint and varnish remover and as a cleaning and degreasing agent. Also fomerly used as soil fumigant, until 1991 (IARC 1994). It is listed as Proposition 65 carcinogen (California OEHHA 2014). It is listed on the REACH SVHC Candidate list as carcinogenic and toxic for reproduction, with exposures from air and water from the production of chlorinated compounds (ECHA 2011a).	No studies were found using biomarkers of exposure to 1,2,3-trichloropropane
106-93-4	1,2-Dibromoethane (ethylene dibromide)	Halogenated organic solvent	For the general population, the most important current exposure is through contaminated drinking water due to 1,2-dibromoethane's former use as a gasoline additive (NTP 2011). It was also used historically and is still used outside of the US as a pesticide, and exposure may also occur in pest control, petroleum refining and waterproofing. It has been detected in ambient air, soil, groundwater, and food. Historically, concentrations in ambient air were an important source of exposure, especially near automobiles or filling stations (IARC 1999f; NTP 2011). This is a TSCA Work Plan Chemical, identified as a low likelihood of exposure. It is used in commercial and industrial products and present in indoor environments and soil. Relatively small releases to the environment have been reported (US EPA 2012). It is listed as Proposition 65 male developmental toxicant (California OEHHA 2014).	No studies were found using specific biomarkers of exposure to 1,2-dibromoethane, although NHANES measured 2-hydroxyethyl mercapturic acid (HEMA), a common metabolite of 1,2-dibromoethane, vinyl chloride, acrylonitrile, and ethylene oxide, in urine by isotope dilution and HPLC-MS/MS, detecting it in 71% of samples, with higher levels in smokers (Calafat et al. 1999).

CAS	Name	Chemical group	Exposure summary	Biomarker summary
107-06-2	1,2-Dichloroethane	Halogenated organic solvent	The greatest source of exposure to 1,2-dichloroethane for the general population is inhalation of the compound in contaminated air (NTP 2011). It has been detected at low levels in ambient and urban air, groundwater and drinking water due to its former use as a gasoline additive, and it has also been detected in food items, possibly due to its use as an extractant in certain food processes (IARC 1999i; NTP 2011). It is mainly used in the production of vinyl chloride and is a biodegradation product of tetrachloroethane. It is used in some consumer products (adhesives, rug cleaners), and it was historically used as a fumigant (IARC 1999i; NTP 2011). It is a TSCA 2013/2014 Work Plan Chemical, identified as a low likelihood of exposure. It is used in commercial and industrial products, present in biomonitoring, and high releases have been reported to the environment (US EPA 2012). It is listed on the Canadian Priority Substances List, with exposures from ambient and indoor air, surface waters, groundwaters, and drinking water (Health Canada 1994). It is listed as a Proposition 65 carcinogen (California OEHHA 2014). It is listed on the REACH SVHC Candidate List as carcinogenic, with exposures from ambient and urban air, groundwater and drinking-water samples (ECHA 2011b).	NHANES has used HS-SPME-GC-MS on 3 mL (minimum) to 10 mL (optimal) whole blood, but less than 5% of the population had levels above the LOD of 0.01 ng/mL(Blount et al. 2006; CDC 2008b, 2009). Possibly a method to measure non-specific urine metabolites such as haloacetic acids and haloalcohols could be developed.
78-87-5	1,2-Dichloropropane	Halogenated organic solvent	The general population may be exposed via inhalation of ambient air, ingestion of drinking water, and dermal contact with consumer products containing 1,2-dichloropropane (IARC 1986a; NLM 2011). This is a TSCA 2013/2014 Work Plan Chemical, identified as having a high likelihood of exposure. It is used in consumer products and present in biomonitoring, drinking water, indoor environments, and soil. High releases to the environment have been reported (US EPA 2012). It is listed as a Proposition 65 carcinogen (California OEHHA 2014).	NHANES has measured 1,2-dichloropropane in blood samples from the general population, and others have attempted to measure it in the blood of occupationally exposed populations. NHANES has used HS-SPME-GC-MS on 3 mL (minimum) to 10 mL (optimal) whole blood, but less than 5% of the population had levels above the LOD of 0.008 ng/mL (Blount et al. 2006; CDC 2008b, 2009). Occupationally, unmetabolized 1,2 dichloropropane measured by SPME-GC-MS was below the LOD (0.2 µg/L) in the urine of 9 "handicraft" automobile mechanics (Vitali et al. 2006). Possibly a method to measure non-specific urine metabolites such as haloacetic acids and haloalcohols could be developed.
56-23-5	Carbon tetrachloride	Halogenated organic solvent	The general population is most likely exposed to carbon tetrachloride through air and drinking water (NTP 2011). It may be used in paint and varnish remover, cleaning and sanitation products, auto products, and hobby/craft products, and it is found in household plastic and epoxy binders (NLM 2013). It was formerly used as dry cleaning agent, aerosol propellant, pesticide/fumigant and fire extinguisher (NLM 2011). It is detected at low levels in ambient air and water (IARC 1999a). It is a TSCA Work Plan Chemical, identified as low likelihood of exposure. It is present in drinking water and soil. High releases to the environment have been reported (US EPA 2012). It is a Proposition 65 carcinogen (California OEHHA 2014).	NHANES has used HS-SPME-GC-MS on 3 mL (minimum) to 10 mL (optimal) whole blood, but less than 5% of the population had levels above the LOD of 0.005 ng/mL (Blount et al. 2006; CDC 2008b, 2009). Occupationally, carbon tetrachloride has been measured in urine (Gobba et al. 1997), and a method to measure non-specific urine metabolites such as haloacetic acids and haloalcohols could be developed.

CAS	Name	Chemical group	Exposure summary	Biomarker summary
75-09-2	Methylene chloride (dichloromethane)	Halogenated organic solvent	Widespread exposure occurs during the production and industrial use of methylene chloride and during the use of a variety of consumer products containing it. Consumer products that may contain the chemical include: fabric cleaners, furniture polish, paint strippers, wood sealant and stains, spray paints, adhesives, shoe polish, art supplies, (US EPA 2010b) and biomarkers favor and many home maintanance and craft products (NLM 2013). It was used until 1989 as a propellent for hair spray. Substantial losses to the environment lead to ubiquitous low-level exposures from ambient air and groundwater (IARC 1999j; NTP 2011). It is a TSCA 2012 Work Plan Chemical, identified as having a high likelihood of exposure. It is present in drinking water, indoor environments, and soil. High releases to the environment have been reported (US EPA 2012). It is listed as a Proposition 65 carcinogen (California OEHHA 2014).	Methylene chloride has been measured in blood, urine, and exhaled breath in population and occupational studies. NHANES has used HS-SPME-GC-isotope dilution MS on 3-10 mL whole blood, but less than 5% of the population had levels above the LOD 0.07 ng/mL (Blount et al. 2006; CDC 2008b, 2009). Smith et al. (2008) measured methylene chloride and other VOCs via HS SPE GC-MS in urine from 24 healthy elderly men, detecting methylene chloride in all samples; methylene chloride was still detectable after 8 hours frozen or unfrozen storage. Poli et al. (2005) used HS SPME GC-MS with LOD 0.005 µg/L to measure methylene chloride in urine from 120 unexposed individuals, with a median concentration of 0.68 µg/L. In two cases of acute methylene chloride poisoning, Poli et al. (2005) calculated urinary half-lives of 7.5 and 3.8 hours, and blood half-lives of 4.3 and 8.1 hours. Hoffer et al. (2005), using HS SPME-GC, found 0.02-0.06 mg/L methylene chloride in urine from 7 exposed workers. Based on experiments with spiked urine, they stress the importance of promptly sealing urine collection and headspace chamber containers, and of analyzing samples within 2 weeks of collection. Sakai et al. (2002) used HS GC-FID with LLOQ 0.01 mg/L, and found that exposure levels and urinary concentrations were highly correlated in an occupationally exposed group. Delfino et al. (2003) detected methylene chloride in over 75% of 106 exhaled breath samples from 21 Hispanic children with mild asthma living near major sources of vehicle exhaust in LA, but found that ambient VOC measurements were better predictors of symptoms than VOCs in exhaled breath. Thrall et al. (2001) found that concentrations in exhaled breath samples from exposed workers increased by up to 573 ppb after performing tasks involving methylene chloride. Possibly a method to measure non-specific urine metabolites such as haloacetic acids and haloalcohols could be developed.
75-01-4	Vinyl chloride	Halogenated organic solvent	The general population may have some limited exposure to vinyl chloride, particularly through direct or indirect contact with polymer products (IARC 1979). It is used almost exclusively by the plastics industry to produce polyvinyl chloride (PVC), a plastic used in many consumer and industrial products. It was previously used as a refrigerant and in aerosol propellants, including hairsprays, but these uses were banned in 1974 (NTP 2011). It is a TSCA Work Plan Chemical, identified as having a high likelihood of exposure. It is present in drinking water, indoor environments, surface water, ambient air, groundwater, and soil. High releases to the environment have been reported (US EPA 2012). It is listed as a Proposition 65 carcinogen (California OEHHA 2014).	Two studies were found using urine biomarkers of exposure to vinyl chloride (VC), one in the general population, one in an occupational setting. In addition, NHANES has measured 2-hydroxyethyl mercapturic acid (HEMA), a common metabolite of 1,2-dibromoethane, vinyl chloride, acrylonitrile, and ethylene oxide, in urine by isotope dilution and HPLC-MS/MS, detecting it in 71% of samples, with higher levels in smokers (Calafat et al. 1999). CDC is planning to add UPLC-ES-MS/MS testing for HEMA (LOD 0.6 ng/mL) to future NHANES reports (Alwis et al. 2012). Gonzalez-Reche et al. (2002) used HPLC-ESI-MS/MS (and confirmatory GC-MS) to detect etheno-DNA adducts (1,N2-ethenoguanine, N2,3-ethenoguanine), in urine from 13 healthy subjects without known occupational exposure to industrial chemicals such as VC and ethyl carbamate, exposure to both of which forms such adducts. They found adducts in the range <0.3-8 nmol/l, and proposed endogenous mechanisms for the formation of adducts at these “background” levels. Chang et al. (2001) measured levels of the VC metabolite thiodiglycolic acid (TdGA) in the urine of 16 PVC manufacturing workers at the end of one shift and at the beginning of the next. This study found a significant difference in TdGA levels for workers exposed to more than v less than 5 ppm VC, and a significant correlation between air VC concentration and urinary TdGA concentration. TdGA levels were higher at the beginning of a shift than at the end of the previous one.
75-02-5	Vinyl fluoride	Halogenated organic solvent	Vinyl fluoride is used in the production of polyvinylfluoride which has been used to cover walls, pipes, and electrical equipment and inside aircraft cabins (NTP 2011). It is listed as a Proposition 65 carcinogen (California OEHHA 2014).	No studies were found using biomarkers for vinyl flouride.
75-35-4	Vinylidene chloride	Halogenated organic solvent	The general population may be exposed via inhalation of ambient air, ingestion of food and drinking water, and dermal contact with consumer products, such as plastic wrap which contains residual monomer (NLM 2011). Migration of vinylidene chloride into food wrapped in plastic is likely. It is detected in wastewater (IARC 1999l).	NHANES has used HS-SPME-GC-MS on 3-10 mL whole blood, but less than 5% of the population had vinylidene chloride levels above the LOD of 0.009 ng/mL (Blount et al. 2006; CDC 2008b, 2009). Waksman and Phillips (2004) briefly review research on the metabolism and biomonitoring of vinylidene chloride, noting that metabolites such as dithioglycolic acid are sometimes used, but that many DCE metabolites are also metabolites of other chlorinated hydrocarbons.
62450-07-1	3-Amino-1-methyl-5h-pyrido[4,3-b]indole (Trp-P-2)	Heterocyclic amine	Consumption of charred fraction of cooked fish is a source of exposure for the general population (IARC 1983b). It is listed as a Proposition 65 carcinogen (California OEHHA 2014).	Trp-P-2 has been measured in the blood, urine, and bile of healthy volunteers and hospital patients. Manabe et al. (1992) used HPLC on plasma and red blood cells from healthy volunteers and patients with uremia, detecting Trp-P-2 at higher levels in uremic patients but also detecting it in samples from healthy subjects. Ushiyama et al. (1991) used HPLC on urine from 10 healthy volunteers on "normal" diets and 3 patients on IV feeding. Trp-P-2 levels ranged from 0.03-0.68 ng in 24h urine samples from healthy volunteers, with none detected in the patients' urine. Using HPLC on human bile from seven subjects with catheterized bile ducts and external biliary drainage, Manabe et al. (1990) found an average of 864 fmol Trp-P-2 excreted per day. Baranczewski et al. (2004) was able to detect Trp-P-2-DNA adducts in the livers of mice dosed with Trp-P-2.
76180-96-6	2-Amino-3-methylimidazo[4,5-f]-quinoline (IQ)	Heterocyclic amine	Exposure occurs primarily through the consumption of cooked meats; it is also detected in processed food flavorings, beer, wine, and cigarette smoke (IARC 1993a, c; NTP 2005). It is listed as a Proposition 65 carcinogen (California OEHHA 2014).	No studies were found using biomarkers to measure exposure to IQ in humans, though methods have been developed for measuring IQ and its metabolites in urine. Yoxall et al. (2004) developed a method for the extraction of IQ and other heterocyclic amines from human urine using blue rayon. Gerbl et al. (2004) describes HPLC with coulometric electrode array detection for measurement of IQ in rat urine. Hsu et al. (2009) and Lakshmi et al. (2009) identify multiple metabolites in the urine of mice dosed with IQ.

CAS	Name	Chemical group	Exposure summary	Biomarker summary
77094-11-2	2-Amino-3,4-dimethylimidazo(4,5-f)quinoline (MeIQ)	Heterocyclic amine	Exposure occurs primarily through the consumption of cooked meats; MeIQ is also detected in processed food flavorings, beer, wine, and cigarette smoke (NLM 2011; NTP 2011). The associated chemical MeIQx has also been found in air and surface water (NTP 2011). It is listed as a Proposition 65 carcinogen (California OEHHA 2014).	No studies were found using biomarkers to measure exposure to MeIQ in humans, though one method exists for measuring MeIQ in urine: Gerbl et al. (2004) describes HPLC with coulometric electrode array detection for measurement of MeIQ in rat urine.
105650-23-5	PhIP	Heterocyclic amine	Exposure occurs primarily through the consumption of cooked meats, and PhIP has been also detected in processed food flavorings, beer, wine, and cigarette smoke. It is present in air and surface water (NTP 2011). It is listed as a Proposition 65 carcinogen (California OEHHA 2014).	Many studies have measured exposure to PhIP in the general population or in volunteers fed cooked meat. Studies used blood, urine, hair and pancreatic tissue. Magagnotti et al. (2000) measured serum albumin (SA) and globin (Gb) adducts in blood by GC-MS and LC-MS/MS. Magagnotti found PhIP-SA levels (mean +/- SD) of 6.7 +/- 1.6 and 0.7 +/- 0.3 fmol/mg, and PhIP-Gb levels of 3.0 +/- 0.8 and 0.3 +/-0.1 fmol/mg in meat eaters and vegetarians respectively. Ushiyama et al. (1991) used HPLC to measure PhIP in urine of 10 healthy volunteers eating their normal diets and 3 patients on IV nutrition, and found 0.12-1.97 ng in 24-h samples from the healthy volunteers and no PhIP in the IV-fed patients' urine. Viberg et al. (2006) used SPE-capillary electrophoresis-MS to detect PhIP in urine, with a DL of 65 pM, finding 1.8 nmol/L (=0.4 pg/ul) PhIP in urine collected 12 h after subjects had eaten fried chicken. Reistad et al. (1997) used GC-MS to measure PhIP in urine, measuring 2-23 ng or 24-100 ng (depending on sample prep) in 24-h urine samples from subjects who had eaten cooked meat. Walters et al. (2004) found the metabolites N(2)-OH-PhIP-N(2)-glucuronide and N(2)-OH-PhIP-N(3)-glucuronide in urine by LC-MS/MS, with levels varying depending on whether subjects were avoiding or eating cruciferous vegetables. Kulp et al. (2004) analyzed the same two metabolites as Walters et al. (2004), as well as 4'-PhIP-sulfate, and found all three in the urine of 8 volunteers after they had eaten cooked chicken, but not before the meal. Bessette et al. (2009) used LC-MS/MS for PhIP detection in hair, with LLOQ around 50 pg/g. This study found PhIP at 290-890 pg/g in the hair of meat eaters, and from below the LOD to 65 pg/g in vegetarians. Alexander et al. (2002) describes findings of <50-5000 pg PhIP/g hair. Kobayashi et al. (2007) determined that hair PhIP levels correlate with those estimated from a food frequency questionnaire, if hair results are adjusted for melanin. Zhu et al. (2006) described immunochemistry and image analysis of DNA adducts in pancreatic tissues from pancreatic adenocarcinoma patients and from healthy volunteers. Zhu et al. (2006) found adducts in 53/54 tissue samples from controls, 39/39 samples of tumor tissue, and 34/38 samples of non-cancerous tissues from cancer patients. Mean values (+/- SD) of absorbancy for PhIP staining were 0.22 +/- 0.04, 0.24 +/- 0.03, 0.24 +/- 0.04, respectively. Using 0.3 mL plasma, Lezamiz et al. (2008) described LC-MS/MS, with a DL of 6 pg/mL and a DOQ of 11 pg/mL. Busquets et al. (2009) used the same method as Lezamiz et al. (2008) for PhIP detection in urine, with a DL of 2pg/g. Gu et al. (2010) also used LC-MS/MS on urine, obtaining LLOQs of 5 pg/mL for PhIP, 5 mg/mL for HONH-PhIP, and 20 pg/mL for the glucuronide conjugates of PhIP and HONH-PhIP. Frandsen et al. (2002) measured 5-OH-PhIP with LC in rat urine and feces and in the urine of one human subject who had recently eaten cooked beef.
81-11-8	Amsonic acid	Hormone or EDC	Potential sources of exposure include clothing, especially when moistened by perspiration, packaging materials, some foods, such as fish, and insufficiently rinsed dishes. There is little if any direct use of the parent compound by consumers. It is used in the manufacture of dyes and fluorescent whitening agents or optical brighteners with a range of uses, including in laundry detergents (NTP 1992).	No studies were found using biomarkers for exposure to amsonic acid.
1912-24-9	Atrazine	Hormone or EDC	The general population may be exposed to atrazine via inhalation of ambient air, ingestion of drinking water, and ingestion of foods that may contain atrazine (NLM 2011). It is a commonly used herbicide and found widely, together with its dealkylated degradation products, in rivers, lakes, estuaries, groundwater and reservoirs (IARC 1999g).	NHANES, NIOSH, and others have measured atrazine and its metabolites in urine of the general population and exposed farmers. NHANES 2003-2004 measured atrazine and 5 metabolites in urine samples from the general population, but has withdrawn those data "due to unacceptable measurement variance at or near the LOD" (CDC 2011) NHANES 2001-2002 measured urinary atrazine mercapturate, which was not detectable in most samples over the LOD of ~1 µg/L (CDC 2009). NIOSH method 8315, exposure to triazine herbicides (NIOSH 2003) measures atrazine, desethyl atrazine, and desisopropyl atrazine via GC-MS in at least 15 mL urine, LOD 20-47 nmol/L. Panuwet et al. (2010) developed a method using SPE-HPLC-MS/MS with isotope dilution quantification to measure atrazine and 6 metabolites, with LODs between 0.05-0.19 ng/mL. Curwin et al. (2010) describes immunoassay to quantify atrazine mercapturate and other pesticide metabolites as cheaper and faster than HPLC-MS/MS but more likely to overestimate exposure. Barr et al. (2007) used SPE-HPLC-MS/MS to measure atrazine and 9 metabolites in urine from a small sample of people with varied exposure levels, and found highly varied metabolite profiles, concluding that it is important to measure multiple metabolites, that measuring only atrazine and atrazine mercapturate underestimates atrazine exposure, and that the most informative metabolites are diaminochloropropyl triazine and desethylatrazine. Chevrier et al. (2011) measured multiple atrazine metabolites, detecting atrazine or atrazine mercapturate in the urine of 5.5% of pregnant women (n= 579) in Brittany in 2002-2006, while dealkylated and hydroxylated triazine metabolites were detectable in 20% and 40% of samples, respectively. Multiple studies (Bakke et al. 2009; Curwin et al. 2010; Mendas et al. 2012) have measured atrazine mercapturate in the urine of farm workers, their families, and controls, and have found that elevated levels persist after pesticide application.

CAS	Name	Chemical group	Exposure summary	Biomarker summary
12789-03-6	Chlordane	Hormone or EDC	Although use of this organochlorine insecticide has been banned, human exposure continues because of its persistence in the environment, especially in indoor air in previously treated buildings and in meat, fish and other fat-containing foodstuffs (IARC 2001b). It was used starting in the 1950s for termite control, on agricultural crops, on lawns, on livestock, and for other purposes, and is commonly detected in indoor air and house dust in the US (Rudel et al. 2003).	NHANES and others have measured chlordane and its metabolites in the blood of the general population, and some studies have measured chlordane or its metabolites in human breast milk and adipose tissue. NHANES measured the chlordane metabolites oxychlordane and trans-nonachlor in serum via SPE followed by gas chromatography/isotope dilution high-resolution mass spectrometry (CDC 2006, 2013; Everett and Matheson 2010; Lee et al. 2006) in 1999-2001, 2001-02, and 2003-04. Others have also measured cis-chlordane, trans-chlordane, and cis-nonachlor in blood (Cao et al. 2012; Rudge et al. 2012; Varona et al. 2010). Many studies (Haraguchi et al. 2009; Hedley et al. 2010; Tanabe and Kunisue 2007; Zhou et al. 2011) have used GC-MS or other methods to measure chlordane or chlordane related chemicals in human breast milk, mostly in Asia. A few researchers have measured chlordane in human adipose tissues (Kunisue et al. 2006; Kutz et al. 1991; Nakata et al. 2005).
NA	Conjugated estrogens	Hormone or EDC	Conjugated estrogens can be measured in domestic wastewater and surface water polluted by wastewater, following urinary excretion. They are used for estrogen replacement therapy and oral contraceptives (Kolpin et al. 2002). The use of postmenopausal estrogen therapy became common in the United States in the 1960s. By 1967, approximately 13% of the women in the United States 45 to 64 years old used this type of therapy. The number of prescriptions for estrogens, not counting those used for oral contraceptives, increased from approximately 15 million in 1966 to more than 25 million in 1976, when prescriptions declined because of concerns about endometrial cancer, but then increased rapidly to approximately 40 million by 1992. In 2002, more than 100 million prescriptions were filled for brand-name and generic products containing estrogens (either conjugated or esterified) as an active ingredient (NTP 2011). Observed increased breast cancer risk associated with exposure to pharmaceutical estrogens has raised concern about possible risk associated with chemicals that mimic estrogen or are endocrine disruptors. Most commercial chemicals have not been screened for endocrine disruption. Also, as discussed by Rudel et al. (2007; 2011), the typical cancer bioassay design may not be sensitive to hormonally-induced mammary tumors. Conjugated estrogens are listed as Proposition 65 carcinogens (California OEHHA 2014).	See summary for estradiol. Many methods used to measure non-conjugated estrogens can also measure conjugated estrogens in blood and urine either by quantifying the conjugated forms specifically via LC-MS/MS and presumably GC-MS/MS (Ziegler et al. 2010), or by subtracting concentrations of unconjugated hormone from total hormone concentration measured after lysis of the conjugate groups (Blair 2010).

CAS	Name	Chemical group	Exposure summary	Biomarker summary
56-53-1	Diethylstilbestrol	Hormone or EDC	DES is a synthetic estrogen that was prescribed to pregnant women from the 1950s until the early 1970s to prevent miscarriage. It was later shown to be a transplacental carcinogen, causing a rare vaginal cancer in daughters of exposed women. It has been demonstrated to increase breast cancer risk in exposed mothers and their daughters, and to cause reproductive system abnormalities (Hoover et al. 2011). An estimated 5-10 million people were exposed in utero in the US (IARC 2012; NTP 2011). DES is now occasionally used to treat prostate cancer, but this use is rare because of its side-effects. It is occasionally used in postmenopausal women with breast cancer (IARC 2012). It has also been found in animal feed (NLM 2004) and was historically used as a growth promoter in sheep and cattle (NTP 2011). It is listed as a Proposition 65 carcinogen and developmental toxicant (California OEHHA 2014)	A few studies have described methods for measurement of DES in human urine, and more have measured it in bovine urine. Zou et al. (2012) used HPLC with novel extraction methods to detect DES with LOD 0.1 ng/mL in human urine, while Wu et al. (2009) used GC-MS in human urine, with a limit of detection of 0.28 ng/mL, and concluded that their method was sufficiently sensitive to use for "routine assessment and monitoring... in the human body". Measurements of DES in bovine urine have used LC-MS/MS (Kaklamanos et al. 2009; Schmidt et al. 2008), HPLC-MS (Rubies et al. 2007), LC-ES-MS (Msagati and Nindi 2006) and GC-MS (Aman et al. 2006; Dickson et al. 2003).
50-28-2	Estradiol-17b	Hormone or EDC	Estradiol can be measured in domestic wastewater and surface water polluted by wastewater, following urinary excretion (Kolpin et al. 2002). It is used pharmaceutically as an estrogenic hormone, estrogen replacement therapy, and oral contraceptive (NLM 2011). Monitoring data indicate that the general population may be exposed to estradiol at well below the therapeutic dose via ingestion of drinking water and dermal contact with contaminated sediments (NLM 2004). It is listed as a Proposition 65 carcinogen (California OEHHA 2014).	Clinical and research laboratories have used many methods to measure estradiol and other steroid hormones in human blood and urine. "Direct" radio immunoassay (RIA), with no extraction or chromatography steps, is commonly used to measure estradiol in serum or plasma in clinical practice, but is imprecise and prone to interference from other hormones and hormone-binding proteins in serum or plasma (Blair 2010; Cao et al. 2004; Rosner et al. 2013). Additionally, standard direct RIA can't detect the low levels found in small blood samples from people other than healthy premenopausal adult women (Blair 2010; Rosner et al. 2013). HPLC-RIA is somewhat more sensitive and precise, but suffers from many of the same problems as direct RIA (Blair 2010). GC-MS and LC-MS methods in blood and urine are more precise, sensitive, and specific, but many require specialized derivatization and ionization steps to achieve low detection limits (Blair 2010; Rosner et al. 2013; Stanczyk and Clarke 2010). Additional reviews are available (Honour 2006, 2010; Kushnir et al. 2010; McDonald et al. 2011; Taylor 2006). The MCF-7 cell proliferation assay has been used to measure estrogenic activity in extracts of adipose tissue in breast cancer cases and controls (Fernandez et al. 2007). The development of methods to conduct a similar assay in blood, and to distinguish endogenous and exogenous estrogen signals, would allow integrated assessments of exposure to xenoestrogens.
53-16-7	Estrone	Hormone or EDC	Unspecified estrogen and estrogenic hormones, which are believed to consist primarily of estrone, have been used in hormonal skin preparations (less than 0.1% to 5%), moisturizing lotions (1% to 5%), wrinkle-smoothing creams, hair conditioners, hair straighteners, shampoos, and grooming aid tonics (less than 0.1%) (NTP 2011). There are case reports of children with premature breast development associated with these exposures. It is listed as a Proposition 65 carcinogen (California OEHHA 2014).	See summary for estradiol. Methods used to measure estradiol can also measure estrone (Blair 2010; Stanczyk and Clarke 2010; Ziegler et al. 2010).

CAS	Name	Chemical group	Exposure summary	Biomarker summary
57-83-0	Progesterone	Hormone or EDC	Human placental extracts, of which progesterone is believed to be the main constituent, have been used in preparations for cosmetic use (at levels of 0.1% to 1.0%), hair conditioners, shampoos, and grooming aid tonics (<0.1%). There are case reports of children with premature breast development associated with these exposures. Progesterone has been detected in cow's milk, milk products, certain plant species, and meat from animals treated with a progesterone implant. Progesterone is also used in pharmaceuticals including birth control (NTP 2011). Monitoring data indicate that the general population may be exposed to progesterone at well below the therapeutic dose via ingestion of drinking water (NLM 2011). It is listed as a Proposition 65 carcinogen (California OEHHA 2014).	See summary for estradiol. Methods similar to those used to measure estradiol can also measure progesterone in blood and urine (Honour 2010; McDonald et al. 2011; Stanczyk and Clarke 2010). Progesterone has also been measured in human saliva (Honour 2010).
122-34-9	Simazine	Hormone or EDC	The general population may be exposed to simazine via ingestion of contaminated drinking water, ingestion of food, and inhalation of ambient air (NLM 2011). Exposure could also occur through consumption of foods containing residues, though residues were not detected in large-scale surveys of food products in Canada and the USA (IARC 1991a). It is a widely used herbicide to control grasses and weeds in food crops, and it is also used for selective control of algae and submerged weeds in ponds, large aquaria, ornamental fish ponds, and fountains. Prior to 1994 it was approved for use to control algae in hot tubs and swimming pools. Simazine and its degradation products have been detected at low levels in ambient rural and urban air, rainwater, surface and groundwater and, less frequently, in drinking water samples (IARC 1999h). It is listed as a Proposition 65 carcinogen (California OEHHA 2014).	At least one study has measured simazine and its metabolites in the urine of the general population, and NIOSH includes it in a published method. NIOSH method 8315, exposure to triazine herbicides, measures simazine, atrazine, desethyl atrazine, and desisopropyl atrazine via GC-MS in at least 15 mL urine, LOD 20-47 nmol/L. Chevrier et al. (2011) measured multiple urinary simazine metabolites, detecting simazine or simazine mercapturate in the urine of 8% of pregnant women (n= 579) in Brittany in 2002-2006, while dealkylated and hydroxylated triazine metabolites were detectable in 20% and 40% of samples, respectively. Simazine and atrazine are structurally similar and share many metabolites, so methods for measuring atrazine exposure should be applicable to simazine as well.
77439-76-0	MX (3-chloro-4-(dichloromethyl)-5-hydroxy-2(5h)-furanone)	MX	The general population may be exposed via treated (chlorinated) drinking water. MX is a by-product of drinking water disinfection that has been found at nanogram-per-liter levels in drinking water as a result of chlorination or chloramination (IARC 2004). One study identified MX as the primary contributor to genotoxicity of finished drinking water (Brunborg et al. 1991) but other compounds contribute as well. It is listed as a Proposition 65 carcinogen (California OEHHA 2014).	No studies were found using biomarkers to measure exposure to MX. Weisel et al. (1999) and others have used urine trihaloacetic acid levels as markers of exposure to drinking water disinfection by-products.
75321-20-9	1,3-Dinitropyrene	NitroPAH	1,3-dinitropyrene is found at low concentrations in ambient air and associated with diesel exhaust and air pollution (IARC 1989c). It is listed as a Proposition 65 carcinogen (California OEHHA 2014).	No studies were found using biomarkers of exposure to 1,3-dinitropyrene. Methods for 1-nitropyrene may be adapted.
42397-65-9	1,8-Dinitropyrene	NitroPAH	The primary route of potential human exposure is inhalation. Detectable levels have been found in respirable particulates from ambient atmospheric samples. Associated with particulate emissions from diesel engines, kerosene heaters, and gas burners. It has also been found at low concentrations in ambient air (IARC 1989e; NTP 2011). It is listed as a Proposition 65 carcinogen (California OEHHA 2014).	No studies were found using biomarkers of exposure to 1,8-dinitropyrene. Methods for 1-nitropyrene may be adapted.

CAS	Name	Chemical group	Exposure summary	Biomarker summary
5522-43-0	1-Nitropyrene	NitroPAH	The general population may be exposed via inhalation of ambient air, ingestion of food and drinking water, and dermal contact. Diesel exhaust is considered the major source of exposure, and 1-nitropyrene is commonly detected in ambient air. 1-nitropyrene has also been detected in the air near coal plants, in fumes from soybean cooking oil, and in dried herbs (NTP 2011). It is listed as a Proposition 65 carcinogen (California OEHHA 2014).	Metabolites and adducts of 1-nitropyrene have been measured in the urine and blood of the general population and in workers and experimental volunteers exposed to diesel exhaust. Huyck et al. (2010) and Laumbach et al. (2009) measured the 1-nitropyrene metabolite 1-aminopyrene in the urine of volunteers experimentally exposed to diesel exhaust. Zwirner-Baier and Neumann (1999) measured hemoglobin adducts derived from 1-nitropyrene in blood samples from bus garage workers, urban hospital workers, and 14 controls via hydrolysis followed by GC-MS, with LOD 0.01-0.08 pmol/g Hb. (Differences between populations were only evident when multiple NitroPAH adducts were summed). Neumann et al. (1995) used the same method to detect 1-nitropyrene-derived adducts in coke oven workers and controls living in the same area. Toriba (2007), using LC-MS/MS with blue rayon extraction, detected multiple isomers of the 1-nitropyrene metabolites hydroxy-N-acetyl-1-aminopyrene and hydroxy-1-nitropyrene in all tested urine samples from the general population; 4 metabolites had mean levels in the hundreds of pmol/mol creatinine. Seidel et al. (2002) measured metabolites of 1-nitropyrene and other PAHs in pre-and post-shift urine samples from salt miners exposed to diesel exhaust, and found large differences in the levels of many metabolites between smoking and non-smoking workers.
607-57-8	2-Nitrofluorene	NitroPAH	General exposures occur via urban atmospheres, contaminated drinking water supplies and recreational activities at contaminated waterways (NLM 2011). 2-nitroflourene is found at low concentrations in ambient air and detected in particulate emissions from diesel engines, kerosene heaters, and gas burners (IARC 1989d). It is listed as a Proposition 65 carcinogen (California OEHHA 2014).	Two studies were found measuring biomarkers of exposure to 2-nitroflourene in the blood of occupationally exposed workers and controls. Zwirner-Baier and Neumann (1999) measured a hemoglobin adduct derived from 2-nitroflourene in bus garage workers, urban hospital workers, and 14 controls via hydrolysis followed by GC-MS, with LOD 0.01-0.08 pmol/g Hb. (Differences between populations were only evident when multiple nitroPAH adducts were summed). Neumann et al. (1995) used the same method to detect adducts of a 2-nitroflourene metabolite in coke oven workers and controls living in the same area. Newer methods for 1-nitropyrene may be adapted.
57835-92-4	4-Nitropyrene	NitroPAH	The primary route of potential exposure is inhalation. 4-nitropyrene has been measured in diesel exhaust particulate extracts and in particulates derived from coal-burning (NTP 2011). It was found at low concentrations in ambient air in one study (IARC 1989a). It is listed as a Proposition 65 carcinogen (California OEHHA 2014).	No studies were found using biomarkers of exposure to 4-nitropyrene. Newer methods for 1-nitropyrene may be adapted.
7496-02-8	6-Nitrochrysene	NitroPAH	6-nitrochrysene has been measured in diesel exhaust particulate extracts (NTP 2011), and was found in ambient air at a low concentration in one study (IARC 1989b). It is listed as a Proposition 65 carcinogen (California OEHHA 2014).	Two studies were found measuring biomarkers of exposure to 6-nitrochrysene in the blood of occupationally exposed workers and controls. Zwirner-Baier and Neumann (1999) measured hemoglobin adduct derived from 6-nitrochrysene in bus garage workers, urban hospital workers, and 14 controls via hydrolysis followed by GC-MS, with LOD 0.01-0.08 pmol/g Hb. (Differences between populations were only evident when multiple NitroPAH adducts were summed). Neumann et al. (1995) used the same method to detect adducts of a 6-nitrochrysene metabolite in coke oven workers and controls living in the same area.
303-47-9	Ochratoxin A	OTA	Exposure can occur through consumption of contaminated grain, nuts, and pork products. It is a naturally occuring mycotoxin with widespread occurrence in food and animal feed (IARC 1993b; NTP 2011). Ochratoxin-contamination of grains is prevalent in some areas of Balkan countries. Ochratoxin exposure has also been demonstrated in a subset of people in the US exposed to mold-contaminated environments or buildings (Hooper et al. 2009). It is a Proposition 65 carcinogen (California OEHHA 2014).	Many studies have used biomarkers in blood and urine to investigate exposure to ochratoxin a (OTA) in general populations, especially in Turkey and other Mediterranean and Balkan countries. Studies have also measured OTA in human breast milk, amniotic fluid, sputum, and biopsies of the lung, liver, and brain. Scott et al. (2005) reviews methods and results of many studies of OTA in blood, urine, and breast milk. Researchers have used ELISA (Erkekoğlu et al. 2010), LC-MS/MS (Ritieni et al. 2010), IAC HPLC FD (Coronel et al. 2009) and SPE HPLC (Karima et al. 2009) to measure OTA in serum, plasma, and whole blood, and IAC-HPLC-FD (Akdemir et al. 2010), IAC-LC-FD (Duarte et al. 2010) and LC-MS/MS (Ritieni et al. 2010) to measure OTA in urine. Scott et al. (2005) describes various methods of reversed phase LC-FD as the most common for measuring OTA in biological liquids, with typical LODs between 0.01-0.1 ng/mL. Many but not all studies find OTA in the majority of blood, urine, or breast milk samples from the general population (Coronel et al. 2009; Duarte et al. 2010; Erkekoğlu et al. 2010; Gürbay et al. 2009; Lino et al. 2008; Scott 2005). Many studies have found regional and seasonal differences in blood and urine levels (Akdemir et al. 2010; Duarte et al. 2010; Erkekoğlu et al. 2010; Lino et al. 2008; Scott 2005). At least one study found levels higher in healthy workers in food factories than in controls (Iavicoli et al. 2002). Ritieni et al. (2010) used LC-MS/MS on 21 amniotic fluid samples, detecting OTA in one sample at 4.26 µg/l, despite not detecting it in blood or urine from the same woman. Hooper et al. (2009) used immunoaffinity columns and fluorometry (LOD: 2 ppb) on urine, sputum, and biopsies of lung, liver, and brain of patients known to be exposed to toxic molds and control patients. Levels in samples from exposed patients from below the LOD to >10 ppb; there was no detectable OTA in samples from control patients. Kovacs et al. (1995) detected OTA ranging from 0.2-7.3 ng/ml in 38 of 92 colostrum samples via HPLC. Muñoz et al. (2009) used LLE-LC-FD to measure the metabolite ocratoxin alpha (OTalpha) (as well as OTA) in plasma (LOQ 0.1 ng/ml) and urine (LOQ 0.05 ng/ml) from 13 volunteers, and detected OTalpha in all samples at much higher levels than the parent compound. Schaut et al. (2008) described detection via HPLC-FLD and LC-MS/MS of many other metabolites.
56-49-5	3-Methylcholanthrene	PAH	Exposure is associated with the use of 3-MC in biochemical research, and it may also be present in industrial air pollutants, smoke from coal or coke-burners, and in tobacco tar (NLM 2011). It is listed as a Proposition 65 carcinogen (California OEHHA 2014).	No studies were found measuring 3-MC in humans or other mammals. Choudhury et al. (1990) used HPLC to measure 3-MC in catfish plasma. Jones et al. (2009) measured 3-MC in molted snake skin.
57-97-6	7,12-Dimethylbenz[a]anthracene	PAH	Exposure is likely from laboratory situations through dermal and inhalation routes, given the use of DMBA as a research chemical (NLM 2011). It is listed as a Proposition 65 carcinogen (California OEHHA 2014).	Although no studies have used biomarkers of exposure to measure human exposure to DMBA, two studies have developed methods for its measurement in blood, urine, and other media. Yardim et al. (2010) developed an electrochemical DNA based biosensor which could detect DMBA in spiked human urine and other media with LOD ~50 ng/L and LLOQ ~500 ng/L. Yardim et al. (2012) used HPLC with diode array detection for the determination of DMBA in serum, liver, and kidney of rats fed DMBA.

CAS	Name	Chemical group	Exposure summary	Biomarker summary
50-32-8	Benzo[a]pyrene	PAH	Exposure occurs primarily through the smoking of tobacco, inhalation of polluted air, and ingestion of charred foods. Benzo[a]pyrene is found in gasoline and diesel exhaust, cigarette smoke, grilled foods, coal tar and coal tar pitch, soot and smoke, petroleum asphalt, creosote oil, shale oil, and commercial solvents (NTP 2011). It is a TSCA Work Plan Chemical, identified as low likelihood of exposure. It is present in biomonitoring, drinking water, indoor environments, surface water, ambient air, groundwater, and soil (US EPA 2012). It is listed as a Proposition 65 carcinogen (California OEHHA 2014). It is restricted under REACH, with exposures from ambient air, contaminated soils and food, and cigarette smoke (European Commission 2009).	Many studies have investigated markers of benzo[a]pyrene (BaP) in blood in general populations, and in urine from occupationally exposed populations. Methods have also been developed for measuring BaP or markers in breast milk, placental tissue, aborted fetal tissue, follicular fluid, semen, and lung tissue. The most commonly measured biomarkers of BaP exposure are DNA and protein adducts measured in blood. BaP-specific adducts, including BPDE-DNA and BPDE-protein (albumin and hemoglobin) adducts, are typically measured by HPLC-FD or GC-MS (Boysen and Hecht 2003; Käfferlein et al. 2010). Nonspecific "bulky" or "BaP-like" DNA adducts have been detected by 32-P-post labeling and immunoassays, which are more sensitive but typically cannot differentiate between PAHs (Boysen and Hecht 2003; Käfferlein et al. 2010). Epidemiologists have used immunoassays to measure nonspecific PAH-DNA adducts in cancer studies (Gammon et al. 2002; Rundle et al. 2002). Researchers have had limited success differentiating between exposed and unexposed populations through use of BaP-specific adducts (Boysen and Hecht 2003; Käfferlein et al. 2010). Singh et al. (2008) used HPLC-FP/UV to detect BaP itself in blood from children in India, finding a median level of 4 ppb. Although at least one study using HS-SPME (Waidyanatha et al. 2003) failed to detect BaP itself in urine from exposed coke plant workers, multiple studies have detected 3-OHBaP in urine from occupationally exposed workers using HPLC (Szaniszló and Ungváry 2001) or LLE-GC-MS (Rossella et al. 2009). Szaniszlo and Ungvary (2001) found higher levels of 3-OHBaP in urine from smokers than non-smokers, but found no difference between healthcare workers, policemen, and asphalt production workers. BaP exposure has been measured in many other biological media as well. Madhavan and Naiku (1995) found "relatively high concentrations" of BaP in umbilical cord blood and breast milk collected from women in India. Toriba et al. (2003) developed an HPLC-FD method to measure BaP and other PAHs in human hair. Singh et al. (2008) found BaP levels measured by HPLC-FD elevated, but not significantly, in 29 placental tissues from preterm pregnancies, compared to 31 from full-term pregnancies. BaP has also been measured in lung samples collected during surgery (Bartsch et al. 1993) or autopsy (Lodovici et al. 1998); in both cases, levels of BAP in lung tissue were correlated with levels of BAP derived DNA adducts. Additional studies have measured BaP or its metabolites or adducts in aborted tissue (Wu et al. 2010), follicular fluid, in which levels were higher in smokers than non-smokers (Neal et al. 2008), and semen (Zenzes et al. 1999). For reviews, see Kafferlein et al. (2010), Benford et al. (2010), Dybing et al. (2008), and Boysen and Hecht (2003).
53-70-3	Dibenz[a,h]anthracene	PAH	Exposure occurs primarily through the smoking of tobacco, inhalation of polluted air and by ingestion of food and water contaminated with combustion products (IARC 1983d). DBA is a TSCA Work Plan Chemical, identified as low likelihood of exposure. It is present in indoor environments, surface water, ambient air, groundwater, and soil (US EPA 2012). It is listed as a Proposition 65 carcinogen (California OEHHA 2014).	General population, occupational, and method development studies have measured exposure to dibenz[a,h]anthracene (DBA) in blood cells, placenta, hair, lung tissue, and nasal cells. Peluso (2004), studying smokers, measured specific DNA adducts for DBA and DB(a,l)P in lymphocytes and nasal and bronchial mucosa, finding the highest levels in nasal mucosa. 10^8 relative adduct levels (mean ±SD) were 1.10 ± 0.66 in nasal samples, 0.82 ± 0.36 in lung samples and 0.54 ± 0.39 in lymphocytes. Gladen et al. (2000) measured DBA and 5 other PAHs in placenta from 200 women from 2 cities in Ukraine, and found a median of 0.73 ng/g dry weight. DBA levels were higher in the larger city, and higher in placentas from mothers with higher BMIs. Liodovici et al. (1998) measured dibenz[a,h]anthracene in lung tissue from autopsied smokers (0.18 ± 0.17 ng/g dry weight, mean ±SD), ex-smokers (0.7 ± 0.03), and non-smokers (0.046 ± 0.025). Toriba et al. (2003) developed a method for analyzing DBA and other PAHs in human hair with HPLC and fluorescence detection, finding DBA in some but not all hair samples.
191-30-0	Dibenzo[def,p]chrysene	PAH	Exposure occurs mainly through the smoking of tobacco, inhalation of polluted air, and ingestion of food and water contaminated with combustion products (IARC 1983c). It is listed as a Proposition 65 carcinogen (California OEHHA 2014).	One study of smokers measured exposure to dibenzo(def,p)chrysene (also known as dibenzo(a,l)pyrene or DB(a,l)P) in human blood, nasal cells, and lung cells. Another study used mouse skin. Peluso (2004), studying smokers, measured specific DNA adducts for DB(a,l)P and DBA in nasal and bronchial mucosa, as well as in lymphocytes, finding the highest levels in nasal mucosa. Mean 10^8 relative adduct levels were 1.10 in nasal samples, 0.82 in lung samples and 0.54 lymphocytes. Roberts et al. (2001) describe using HPLC-fluorescence line-narrowing spectroscopy to measure and analyze DNA adducts in mouse skin that had been exposed to DB(a,l)P.
335-67-1	Perfluorooctanoic acid	PFOA	PFOA is widely detected in blood samples in the US. It is used in non-stick and stain-resistant coatings on rugs, furniture, clothes, cookware, fire-fighting applications, cosmetics, lubricants, paints, and adhesives. Former use in insecticide and herbicide formulations resulted in its direct release to the environment (NLM 2011). It is an EPA Action Plan Chemical, with exposures from drinking water, aquatic organisms, soil, and consumer products (US EPA 2013b).	NHANES and others have measured PFOA in blood from the general population, as well as from groups exposed occupationally or through industrial contamination. PFOA has also been measured in human milk samples and human thyroid tissue. NHANES uses online SPE-HPLC-MS/MS for the measurement of PFOA and 17 other perfluorinated chemicals in 100 μm of serum, with LOD 0.1 ng/mL and LLOQ 0.3 ng/mL (Bartell et al. 2010; Calafat et al. 2007a; Kuklenyik et al. 2005). NHANES has used off-line SPE in the past, with similar results (Calafat et al. 2007b). Sundstrom et al. (2011) measured PFOA at 74 pg/ml in pooled Swedish human milk samples from 2008, with LOD in the low pg/ml. Others have also measured PFOA and other PFCs in human milk samples, usually with higher LODs (Fromme et al. 2010; Llorca et al. 2010; Roosens et al. 2010). Pirali et al. (2009) measured PFOA in thyroid tissues from 28 patients undergoing surgery for thyroid diseases, detecting PFOA at between 0.4-4.6 ng/g in all, but at lower levels than in patients' serum. Lau (2007), Houde (2006), and Harada and Koizumi et al. (2009) review other biomonitoring studies of PFOA and other PFCs.
121-66-4	2-Amino-5-nitrothiazole	Pharmaceutical	The general population may be exposed through residues of 2-amino-5-nitrothiazole in food products. It is a synthetic veterinary antiprotozoal agent (NTP 1978a).	No studies were found using biomarkers of exposure to 2-amino-5-nitrothiazole.
23214-92-8	Adriamycin	Pharmaceutical	Adriamycin is a chemotherapeutic drug used to treat a variety of cancers. National Occupational Exposure Survey estimated that 17,132 health-services workers were potentially exposed from 1981-1983. It can be found unchanged in human waste (NTP 2011).	A few studies have described methods for measuring adriamycin in urine. Sottani et al. (2008) measured adriamycin in urine by rp-HPLC-ESI-MS/MS with LLOQ 0.3 μg/L. Sottani et al. (2004) measured adriamycin in urine by HPLC-MS/MS with LLOQ 0.1 μg/L.

CAS	Name	Chemical group	Exposure summary	Biomarker summary
50-18-0	Cyclophosphamide	Pharmaceutical	Cyclophosphamide has been widely used since the early 1950s in the treatment of malignant lymphoma, multiple myeloma, and cancers of the breast, ovary and lung. It has also been used in the treatment of certain chronic diseases, such as rheumatoid arthritis and chronic glomerulonephritis and other nonmalignant diseases (IARC 1981). It is listed as a Proposition 65 carcinogen and developmental toxicant (California OEHHA 2014).	There have been many studies of cyclophosphamide (CP) levels in urine of healthcare workers and a few of CP levels in urine and blood of chemotherapy patients. Fransman et al. (2007), reviewing three studies in the Netherlands, reports that between 1997 and 2000 the proportion of samples with measurable CP levels went down fourfold, and the median concentration in samples with detectable CP went down threefold. Frequency of detection in occupational studies varies widely, with some studies finding CP in no (Turci et al. 2011; Ziegler et al. 2002) or very few (Favier et al. 2003) subjects' urine, and some finding CP in urine from over half of subjects (Minoia et al. 1998; Sessink et al. 1997; Sugiura et al. 2011). In plasma, LC-MS/MS has been used with LOD 0.02 ng/ml (Hedmer et al. 2008). B'Hymer and Cheever (2010) describe LLE-HPLC-MS/MS to detect cyclophosphamide and its metabolite 4-ketocyclophosphamide in urine, with LODs 1 ng/mL for the metabolite and 0.1 ng/mL for CP. Villarini et al. (2011) and Moretti et al. (2011) describe a study design using urinary cyclophosphamide as a biomarker of exposure to antineoplastic drugs, to be compared with biomarkers of DNA damage.
4342-03-4	Dacarbazine	Pharmaceutical	Dacarbazine is used in cancer therapy (NTP 2011) It is listed as a Proposition 65 carcinogen (California OEHHA 2014).	No studies were found measuring biomarkers of exposure to dacarbazine.
54-31-9	Furosemide	Pharmaceutical	Furosemide is a potent, short-acting sulfonamide diuretic chemically similar to the thiazides, commonly used as a medical treatment in a variety of situations ranging from the control of hypertension to the reduction of edema of cardiac, hepatic, or renal origin. It is particularly useful in the management of acute pulmonary edema and may be used in premature infants to promote the diuresis that usually follows birth. The number of prescriptions for furosemide in the United States increased from 16 million in 1973 to 23 million in 1981 (NTP 1989).	Methods have been developed to measure furosemide (FD) concentrations in blood and urine from patients or volunteers receiving FD. Reeuwijk et al. (1992) used LLE-HPLC with florescence detection, with LOD 0.3 ng/ml in plasma from volunteers receiving FD and amiloride. Margalho et al. (2005) obtained a DL of 10 ng/ml in spiked whole blood with SPE-GC-EI-MS. Saugy et al. (1991) measured FD in serum and urine from experimentally dosed volunteers via HPLC-florescence. Zhang et al. (2008) obtained a DL of 0.5 ng/ml in urine using HF(hollow-fiber)-LLLME, HPLC-UV.
126-07-8	Griseofulvin	Pharmaceutical	Griseofulvin is an anti-fungal used to treat infections of skin, hair, and nails (IARC 2001c; NLM 2011). It is listed as a Proposition 65 carcinogen (California OEHHA 2014).	A few studies have measured griseofulvin in plasma or other fluids from patients or volunteers who had been administered griseofulvin, and more describe pharmacokinetic studies. Mistri et al. (2007) described ESI LCM SMS to measure griseofulvin, with LLOQ 20 ng/mL, in plasma from six healthy subjects after they ingested 500 mg griseofulvin as part of a bioequivalence study. Pacifici (2006), reviewing transplacental transfer of multiple antibiotics, reports that griseofulvin has been measured in cord plasma as well as maternal plasma. Additional pharmacokinetic studies have measured griseofulvin in human intestinal mucus (Gramatte 1994), and griseofulvin and its metabolites varied fluids from experimental animals (Ahmed and Aboul-Einien 2007; Fujioka et al. 2008; Poullain-Termeau et al. 2008). Gramatte et al. (1994) measured griseofulvin in intestinal mucus as part of an availability study.
53-86-1	Indomethacin	Pharmaceutical	Indomethacin is a non-steroidal anti-inflammatory used to treat rheumatoid disorders (NLM 2011, 2014).	Many studies have measured indomethacin in human blood and urine for pharmacokinetics studies or patient monitoring. Methods to measure indomethacin in plasma and serum include LC (Al Za'abi et al. 2006; Miksa et al. 2005; Wang et al. 2013), "micellar electro-kinetic chromatography-UV detection" (Lin et al. 2006), and GC-MS (Thomas et al. 2010). Al Za'abi et al. (2006) describe HPLC with LLOQ 25 ng/mL in plasma from neonates, and Mannila et al. (2007) report levels as low as 0.3 mg/ml in protein free plasma, but do not provide methods information in the abstract. In urine, LC-based methods are apparently the most common (Cruz-Vera et al. 2009; Michail and Moneeb 2011; Riano et al. 2012; Wang et al. 2013), although Molina-Garcia et al. (2010) described "sequential injection analysis and opto sensing" with LOD 0.15 ng/mL and LLOQ of 0.5 ng/mL in 1 mL urine, making it more sensitive than most LC methods. Mannila et al. (2007) measured indomethacin in cerebrospinal fluid (CSF) and plasma from in children who had been administered IV indomethacin, finding plasma levels always at least 100 times CSF levels.
54-85-3	Isoniazid	Pharmaceutical	Isoniazid is an anti-infective agent, used to treat tuberculosis. The National Occupational Exposure Survey estimated that 2,924 workers (1,480 female) were potentially exposed to isoniazid in the US from 1981-1983 (NLM 2011)	Isoniazid has been measured in blood, urine, breastmilk, and cerebrospinal fluid from patients prescribed isoniazid for clinical monitoring and pharmacokinetics studies. However, Peloquin (2002) reported that limits of detection were generally only sufficient to measure isoniazid within a few hours of dosing. HPLC appears to be the most common method for measurement in plasma and serum (Ge et al. 2008; Huang et al. 2009; Xu et al. 2013; Zhao et al. 2011; Zhou et al. 2010), with detection and quantification limits around 0.2-0.5 µg/mL (Huang et al. 2009; Zhou et al. 2011). Other methods used in blood include "cation selective exhaustive injection sweeping micellar electrokinetic chromatography" (Tsai et al. 2011). HPLC and other methods have been used to measure isoniazid and metabolites in urine (Bergamini et al. 2010; Espinosa-Mansilla et al. 2002; F Li et al. 2011; Nicolau et al. 2012; Zhou et al. 2009). Isoniazid has also been measured in cerebrospinal fluid (Donald 2010; Sullins and Abdel-Rahman 2013) and breastmilk (N Singh et al. 2008).
443-48-1	Metronidazole	Pharmaceutical	Metronidazole is an anti-infective agent administered orally, topically, or through injection (NLM 2011). Used in pet fish care products (NLM 2013). It is listed as a Proposition 65 carcinogen (California OEHHA 2014).	Many studies have measured metronidazole in the blood, urine, and other bodily fluids of patients and volunteers dosed with metronidazole. De Freitas Silva (2012), Cohen-Wolkowiez (2011), Suyagh (2011), and do Nascimento (2005) used various LC methods to measure metronidazole in plasma. Salem (2012) used NMR to measure metronidazole in plasma and urine, with LLOQ 0.28 ng/mL. Jafari (2009) used "IM mobility spectrometry" to measure metronidazole in serum, with LLOQ 0.5 µg/mL. Liu et al. (2013) used a grapheme-based electrode material to measure metronidazole in urine (and lake water), with LOD 2.3 nM and LLOQ of 10 nM. Sun et al. (2012) used HPLC with photo diode array detector to measure metronidazole in urine with LOD 0.1 µg/mL. Salem et al. (2006) used NMR to measure metronidazole in urine as well as saliva and gingival crevicular fluid.
139-94-6	Nithiazide	Pharmaceutical	Exposure may result from the use of nithiazide in veterinary medicine (IARC 1983a). It may persist in the tissues and eggs of treated poultry (NTP 1979).	No studies were found using biomarkers to measure exposure to nithiazide.

CAS	Name	Chemical group	Exposure summary	Biomarker summary
67-20-9	Nitrofurantoin	Pharmaceutical	Nitrofurantoin has been used since 1972 in treatment of urinary tract infection (IARC 1990). It is a Proposition 65 male toxicant (California OEHHA 2014).	Some studies described testing of human urine, plasma, and breast milk after administration of nitrofurantoin. Muth et al. (1996) described LLE-HPLC to detect nitrofurantoin in plasma and urine, with LLOQ 0.01 µg/ml in plasma and 0.38 µg/mL in urine. Aufrere et al. (1977) described HPLC in 0.2 mL samples of plasma and urine, with LLOQ 0.02 µg/ml in both media. Arancibia et al. (2003) used SPE-HPLC to detect nitrofurantoin and a radical anion metabolite in urine with detection limits of 12.1 uM and 0.9 uM, respectively. Xu et al. (2009) used an immunochromatographic assay to detect the nitrofuran metabolite 1-aminohydantoin in urine with a detection limit of 10 ng/ml. Pons et al. (1990) used HPLC to measure nitrofurantoin levels in breast milk and blood of women taking nitrofurantoin, quantifying levels in the 10s of ug in 6-h milk samples (Pons et al. 1990).
59-87-0	Nitrofurazone	Pharmaceutical	Nitrofurazone is a synthetic furan derivative active against a broad spectrum of bacteria, and has been used widely in veterinary and human medicine as well as (NTP 1988) pet care products, specifically fish care (NLM 2013). It is listed as a Proposition 65 carcinogen (California OEHHA 2014).	No occupational or general population studies were found for nitrofurazone, but method development studies described testing of human urine and plasma. Du et al. (2007) used flow induction chemiluminescence, with LOD 20 ng/ml and LLOQ 100ng/ml in both plasma and urine. Aufrere et al. (1977) used HPLC, with LLOQ 200 ng/mL in plasma and urine, to measure nitrofurantoin and stated that the method was also applicable to nitrofurazone.
62-44-2	Phenacetin	Pharmaceutical	Until 1983, phenacetin was used in over-the-counter remedies for pain and fever; however, it no longer is used in drug products in the United States. Also, it was once used as a stabilizer for hydrogen peroxide in hair-bleaching preparations (NTP 2011). It is listed as a Proposition 65 carcinogen (California OEHHA 2014).	Some older methods-development and dosing experiment studies have investigated possible biomarkers of phenacetin. Gotelli et al. (1977) used HPLC on 0.1 ml plasma samples, obtaining LLOQ 0.5 µg/ml for phenacetin. Garland et al. (1977) used GLC-chemical ionization MS on 1 ml plasma samples, obtaining a "sensitivity limit" of 1 ng/ml. Murray and Boobis (1991) used GC-MS to quantify phenacetin and its metabolite paracetamol (Tylenol) in plasma, detecting amounts equivalent to 1 pg of parent compound. Davies et al. (1984) used TLC-MS with multiple-peak monitoring for paracetamol in urine. Dittman and Renner (1977) used silica-gel TLC to measure the metabolite 4-acetominophenoxyacetic acid in rat, dog and human urine, detecting 0.04% of a 200 mg/kg dose.
50-55-5	Reserpine	Pharmaceutical	Reserpine is an anti-hypertensive, recently not widely used. The National Occupational Exposure Survey estimated that 5611 workers (2414 female) were potentially exposed to reserpine in the US from 1981-1983 (NLM 2011).	A few studies have described methods for measurement of reserpine in human blood and urine. Tas et al. (1986) used LC-MS with a detection limit around 10 pg to measure reserpine in human serum, for use in testing poisoning victims for overdoses. Owen et al. (1985) described in HPLC method for the analysis of reserpine and related compounds in blood, with detection limits around 50 pg/mL. Li et al. (2011) used HPLC to measure reserpine in human urine with LOD 7.1 ng/mL and LLOQ of 23.6 ng/mL.
52-24-4	Thiotepa	Pharmaceutical	ThioTEPA is used in cancer therapy (NTP 2011).	No studies were found measuring thioTEPA in the general public or in occupational settings, but methods have been developed for blood and urine. Van Maanen et al. (1997) obtained LLOQ 1 ng/ml in 100 ml samples of plasma and urine with capillary GC with a thermionic N-P detector. LC-MS/MS was validated over 5-2500 ng/ml for the metabolite TEPA (and thioTEPA) in 100 ml blood samples (de Jonge et al. 2004). In urine, GC with selective N-P detection, was linear from 25-5000 ng/ml for TEPA, and from 25-2500 ng/ml for the metabolite monochloroTEPA (van Maanen et al. 2000). Also in urine, LC-MS with direct sample injection with sulphadiazine as internal standard was linear from 1-25 µg/ml for the metabolite thioTEPA-mercapture (van Maanen and Beijnen 1999).
100-42-5	Styrene	Styrene	Exposure to the general population occurs at levels of micrograms per day due mainly to inhalation of ambient air and cigarette smoke and intake of food that has been in contact with polystyrene. Styrene is also present in a number of consumer products including carpets, adhesives, hobby and craft supplies and glues, and home maintenance products (IARC 2002; NLM 2013; NTP 2011). It is a TSCA Work Plan Chemical, identified as having a high likelihood of exposure. It is present in drinking water, surface water, ambient air, groundwater, and soil; moderate releases to the environment have been reported (US EPA 2012). It is on the Canadian Priority Substances List, with indoor air exposures from cigarette smoke, and low environmental exposures (Health Canada 1993).	NHANES and others have measured styrene and its metabolites and adducts in blood samples from the general population and urine from general population and occupationally exposed groups, and a few studies have measured styrene in human saliva and breast milk. In 2001-2002 and 2003-2004, NHANES used HS-SPME-GC-MS, LOD 0.03 ng/mL on 3 mL (minimum) to 10 mL (optimal) whole blood and detected styrene in less than half of the general population (CDC 2012b), determining that cigarette smoke is "a primary source of" styrene in the blood of the US population. CDC is planning to add UPLC-ES-MS/MS testing for urinary phenylglyoxylic acid (PGA, a metabolite of styrene and ethyl benzene, LOD 12 ng/mL), mandelic acid (MA, LODs 12 ng/mL), and N-acetyl-S-(1-phenyl-2-hydroxypropyl)-L-cysteine (PHEMA, LOD 0.7 ng/mL) to future NHANES reports (Alwis et al. 2012). A CDC pilot study found that MA and PGA levels were significantly different in smokers and non-smokers, but PHEMA was not detected in the urine of either (Alwis et al. 2012). Fustinoni et al. (2008) found that styrene derived albumin adducts but not hemoglobin adducts were higher in exposed workers than in controls. Reska et al. (2010) used online extraction-HPLC-MS/MS, with a combined LLOQ of 0.3 µg/L, to measure 2 mercapturic acids of styrene in urine from 18 smokers (<0.3-2.8 µg/L, median 0.46 µg/L) and from 22 non-smokers (0.3-1.1 µg/L, median <0.3 µg/L). Gagne et al. (2012) used UPLC-MS/MS for measurement of mandelic acid and phenylglyoxylic acid in urine; neither was detectable in non-occupationally exposed workers, but both were present between 0.2-9 mMol in urine from occupationally exposed workers. Fustinoni et al. (2010) measured urinary styrene, mercapturic acids, mandelic acid, phenylglyoxylic acid, phenylglycine, and 4-vinylphenol conjugates in 10 varnish workers and 8 plastic workers, finding higher levels in plastics workers and good "within worker" reproducibility for most metabolites, and good agreement between metabolite levels and styrene concentrations in air. Many studies have found that styrene metabolism and urinary excretion are influenced by polymorphisms in cyp and GSTM genes (Hirvonen 2005; Prieto-Castello et al. 2010; Rihs et al. 2008; Wang et al. 2009). Mikes et al. (2010) measured two nonspecific N3-adenine DNA adducts in urine by LC ESI MS/MS, with LLOQ 1 pg/mL each adduct, with higher levels in higher exposed workers, but unexplained adduct peaks in some control samples. Blount et al. (2010) used SPE headspace GC selected ion monitoring MS on 3 ml breast milk from 12 women, with a median styrene concentration of 0.129 ng/mL. Sanchez et al. (2012) and Gherardi et al. (2010) describe headspace MS for quantification of styrene in saliva, used by Sanchez et al. (2012) to measure styrene in saliva from 24 healthy volunteers in four patients with various diseases. Many studies of styrene biomarkers are reviewed by Hirvonen (2005), Rueff et al. (2009), and Vodicka (2002a; 2002b).
96-12-8	1,2-Dibromo-3-chloropropane	Miscellaneous	Widespread exposure of the general population is not likely, since use of 1,2-dibromo-3-chloropropane as a soil fumigant was banned in 1985. Exposure of the general population may occur with ingestion of previously contaminated drinking water and food (NTP 2011). It has been detected at low levels in ambient air, water and soil (IARC 1999d). It is listed as a Proposition 65 carcinogen (California OEHHA 2014).	NHANES has used HS-SPME-GC-MS on 3 mL (minimum) to 10 mL (optimal) whole blood, but less than 5% of the population had levels above the LOD of 0.01 ng/mL (Blount et al. 2006; CDC 2008b, 2009).

CAS	Name	Chemical group	Exposure summary	Biomarker summary
123-91-1	1,4-Dioxane	Miscellaneous	Exposure of the general population to 1,4-dioxane could possibly occur from contact with products containing residues of the compound. According to the Consumer Product Safety Commission (CPSC), consumers may possibly be exposed to residual levels of 1,4-dioxane formed during the manufacture of detergents, shampoos, surfactants, and certain pharmaceuticals. It is also found in home and auto-use adhesives (NLM 2013). CPSC reported that the presence of 1,4-dioxane, even as a trace contaminant, is cause for concern and the Commission monitors its use in consumer products. Residues may be present in food packaged in 1,4-dioxane-containing materials or on food crops treated with 1,4-dioxane-containing pesticides (NTP 2011). It is detected in ambient air (IARC 1999c) and monitoring data also indicate that the general population may also be exposed to 1,4-dioxane via ingestion of drinking water (NLM 2004). It is a TSCA Work Plan Chemical, identified as having a high likelihood of exposure. High releases to the environment have been reported (US EPA 2012). It is listed as a Proposition 65 carcinogen (California OEHHA 2014).	No studies were found using biomarkers of exposure to 1, 4-dioxane.
532-27-4	2-Chloroacetophenone	Miscellaneous	The use of "Chemical Mace" to disable attackers causes direct exposure to 2-chloroacetophenone through eye and skin contact and inhalation (NLM 2011).	No studies were found using biomarkers of exposure to 2-chloroacetophenone.
75-55-8	2-Methylaziridine	Miscellaneous	Potential consumer exposure could occur as a result of handling products coated with 2-methylaziridine or its derivatives; however, there are few ongoing consumer uses of this compound (NTP 2011). It is listed as a Proposition 65 carcinogen (California OEHHA 2014).	No studies were found using biomarkers for 2-methylaziridine
107-13-1	Acrylonitrile	Miscellaneous	The general population may be exposed through consumer product usage such as acrylic carpeting, rubber, food containers, and toys or by ingestion of contaminated foods. Foods most likely to contain measureable acrylonitrile are high-fat or highly acidic items, such as luncheon meat, peanut butter, margarine, vegetable oil, or fruit juice. Exposure is thought to be low because there is little migration of the monomer into such products (NTP 2011). Acrylonitrile has been measured in the vapor phase of mainstream tobacco smoke and has been detected rarely and at low levels in ambient air and water (IARC 1999b; NTP 2011). It is found in household spackling and caulk (NLM 2013). It is a TSCA Work Plan Chemical, identified as having a high likelihood of exposure. It is present in indoor environments, surface water, ambient air, and groundwater; high releases to the environment have been reported (US EPA 2012). It is on the Canadian Priority Substances List, with exposure from food packaged in acrylonitrile-based plastics (Environment Canada 2011). It is listed as a Proposition 65 carcinogen (California OEHHA 2014).	Many studies have measured acrylonitrile, its metabolites, or its adducts in the blood and urine of smokers and non-smokers. NHANES has measured 2-hydroxyethyl mercapturic acid (HEMA), a common metabolite of 1,2-dibromoethane, vinyl chloride, acrylonitrile, and ethylene oxide, in urine by isotope dilution and HPLC-MS/MS, detecting it in 71% of samples, with higher levels in smokers (Calafat et al. 1999). CDC is planning to add UPLC-ES-MS/MS testing for HEMA (LOD 0.6 ng/mL) and for the AN-specific mercapturic acid N-acetyl-S-(2-cyanoethyl)-L-cysteine (CYMA; LOD 0.5 ng/mL) to future NHANES reports (Alwis et al. 2012). A CDC pilot study found that CYMA levels were significantly different in smokers and non-smokers (Alwis et al. 2012), consistent with many other reports of higher levels of CYMA (Minet et al. 2011; Scherer et al. 2010; Schettgen et al. 2009) and acrylonitrile (Perbellini et al. 2003a) itself in urine from smokers compared to non-smokers. In blood, acrylonitrile-derived hemoglobin adduct levels are well correlated with self-reported smoking levels (Kütting et al. 2008; Schettgen et al. 2002). Though many studies detected the adduct in both non-smokers and smokers, Schettgen et al. (2010) using isotope dilution GC NCI MS/MS, with LOD 0.5 pmol/g globin did not detect it in the majority of blood samples from non-smokers who were not exposed to secondhand smoke at home, and did detect it in the majority of samples from those exposed. Similarly, Schettgen (2004) detected the adduct in maternal and umbilical cord blood from one smoker, but not from any of the 10 non-smokers tested, or in the umbilical cord blood of their recently delivered infants. Schettgen (2009) and Fennell (2000) found that genotype of glutathione transferase gene had little effect on adduct levels.

CAS	Name	Chemical group	Exposure summary	Biomarker summary
3688-53-7	AF-2 (2-(2-furyl)-3-(5-nitro-2-furyl) acrylamide)	Miscellaneous	AF-2 2-2-furyl-3-5-nitro-2-furyl acrylamide a synthetic nitrofuran derivative used as a food preservative in Japan since at least 1965, but it is not used presently (IARC 1983e). It was withdrawn from the market in 1974 (NLM 2011). It is listed as a Proposition 65 Carcinogen (California OEHHA 2014).	No studies were found using biomarkers to measure exposure to AF-2 2-2-furyl-3-5-nitro-2-furyl acrylamide.
NA	Bracken fern (and its extracted chemicals)	Miscellaneous	Exposure to bracken fern and its constituents occurs by direct ingestion of the fronds in some regions of the world, or by ingestion of dairy products from cattle grazing on the fern. In the past, other uses for bracken fern have been in bread flour and medicinals (IARC 1986b). The component ecdysone is being researched as an insecticide or insect repellent (Hami et al. 2005) and may be sold as muscle growth supplement. It is listed as a Proposition 65 carcinogen (California OEHHA 2014).	No studies were found using biomarkers to measure exposure to bracken fern, nor to ptaquiloside, the major carcinogen it produces.
2425-06-1	Captafol	Miscellaneous	The fungicide captafol is not currently registered for use as a pesticide in the US (NTP 2011). It was widely used after 1961 for the control of fungal diseases in fruits, vegetables, some other plants, and lumber (IARC 1991b; NLM 2011). It is listed as a Proposition 65 carcinogen (California OEHHA 2014).	Multiple studies have measured tetrahydrothalidomide (THPI), a common metabolite of captafol and the related pesticide captan, in blood and urine of farm workers, intentionally dosed volunteers, and populations with high use of pesticides in the home. Most of these studies focus on assessing captan exposure and toxicokinetics. Whyatt et al. (2003) used SPE-isotope dilution GC-high resolution MS with LOD 1 pg/g to measure THPI in plasma from 230 African-American and Dominican mothers from northern Manhattan and their newborn infants, detecting THPI in 50% of maternal samples. Berthet et al. (2011) describes LC-atmospheric pressure chemical ionization-MS/MS to measure THPI and thalidomide in blood (LOD 0.58 µg/L) and urine (LOD 1.47 µg/L), and Berthet (2012a; 2012b) measured toxicokinetics of captan in urine and plasma after voluntary dermal and oral exposure, finding half-lives ranging from 16 to 27 hours depending on route of exposure and matrix. McCauley et al. (2008) found significant differences in urinary THPI between 134 workers in Oregon berry fields (mean 0.14 µg/mL) and control non-agricultural workers (mean 0.078 µg/mL). Hines et al. (2008) found a difference in urinary THPI levels between captan applicators using different methods. De Cock et al. (1995) found THPI levels in urine of fruit growers was strongly tied to captan exposure estimates from skin pads on ankles and neck, and lower in growers using more protective measures.
126-99-8	Chloroprene	Miscellaneous	Although few data are available on environmental occurrence, general population exposures to 2-chloroprene are expected to be very low or negligible (IARC 1999e). It is used almost exclusively for the production of neoprene elastomers and latexes, a synthetic rubber used in the production of automotive and mechanical rubber goods, adhesives, caulks, flame-resistant cushioning, construction goods, fabric coatings, sealants for dams or locks in waterways, roof coatings, fiber binding, and footwear (NTP 2011). It is listed as a Proposition 65 carcinogen (California OEHHA 2014).	One recent study detected biomarkers of exposure to 2-chloroprene in urine from an occupationally exposed population. Biomarkers have also been measured in mouse blood. Eckert et al. (2013) measured elevated levels of 3-chloro-2-hydroxy-3-butenyl mercapturic acid (Cl-MA-III), 3,4-dihydroxybutyl mercapturic acid (DHBMA), and 4-hydroxy-3-oxobutyl mercapturic acid (HOBMA) in the urine of 14 workers occupationally exposed to 2-chloroprene compared to 30 controls. Eckert et al. (2012) detailed their LC-MS/MS method to measure urinary Cl-MA-III, DHBMA, and HOBMA, along with other mercapturic acids, with detection limits between 1.4 and 4.20 µg/L. Hurst and Ali (2007) describe a method, used with mouse erythrocytes, to measure hemoglobin adducts with 1-chloroethenyloxirane, a chloroprene metabolite, using headspace analysis with capillary GC-MS.
1420-04-8	Clonitralid	Miscellaneous	The major route of population exposure to clonitralid is presumably dermal contact with or ingestion of treated water or ingestion of contaminated fish. It is directly applied to control sea lamprey larvae in tributaries to the Great Lakes and widely applied to control water snails (NTP 1978c).	No studies were found using biomarkers to measure human exposure to clonitralid, although a method has been developed to measure it in nonhuman biological samples. Caldow et al. (2009) used reverse phase LC-MS/MS with solvent extraction with 1% acetic acid in acetone and clean-up via mixed-mode anion-exchange SPE to measure clonitralid and other anthelmintics in bovine kidney tissue.
62-73-7	Dichlorvos	Miscellaneous	Household uses of dichlorvos represent the main sources of human exposure (IARC 1991c). The general population may be exposed via inhalation of air and dermal contact when no-pest strips, sprays or flea collars contain this insecticide. Exposure could also result from ingestion of food which has been prepared in rooms where dichlorvos is used for insect control (NLM 2011). It is listed as a Proposition 65 carcinogen (California OEHHA 2014).	NHANES and others have measured urinary dimethyl phosphate (DMP), a metabolite of dichlorvos and many other organophosphate pesticides, in the general population and in exposed subjects. DMP has also been measured in hair samples from unexposed and exposed subjects. More specific biomarkers have been measured in the urine and blood of poisoning victims. Since 1999, NHANES has used isotope dilution GC-MS/MS to measure DMP in urine, with LOD 0.5 µg/L, estimating detectable levels in between 25% and 50% of the population (CDC 2009). Multiple studies have found higher levels of urinary DMP and other OP metabolites in farmworkers and their children (Coronado et al. 2011; Lee et al. 2007) than in nonfarm workers and their children. Urinary DMP levels are also higher in people living closer to farmland (Bradman et al. 2011; Coronado et al. 2011). Tsatsakis et al. (2010) measured DMP and three other dialkyl phosphates in hair samples from the general population and from occupationally exposed subjects via methanolic extraction, derivatization with pentafluorobenzylbromide, and GC-MS, detecting large and significant differences between the groups (DMP was detectable in 63% of general population hair samples and 100% of occupationally exposed samples). Biomonitoring of dichlorvos-specific biomarkers has focused on cases of acute poisoning (often suicide attempts), in which dichlorvos or its metabolites have been detected in hair, blood, and urine (Abe et al. 2008; Heinig et al. 2000; Inoue et al. 2007; Bin Li et al. 2010; B. Li et al. 2010; Musshoff et al. 2002; Takayasu et al. 2001).

CAS	Name	Chemical group	Exposure summary	Biomarker summary
1694-09-3	FD&C Violet no. 1	Miscellaneous	FD&C Violet no.1 was used as a dye for wool, leather, nylon, anodized aluminum, inks, paper, biological stain, wood stain, color additive for foods, drugs, and cosmetics until 1973 (NLM 2011). It is listed as a Proposition 65 carcinogen (California OEHHA 2014).	No studies were found using biomarkers to measure exposure to FD&C Violet no.1.
51630-58-1	Fenvalerate	Miscellaneous	Use as a contact insecticide releases fenvalerate directly to the environment in sprays, dusts, concentrates and other routes of application (NLM 2011). It is detected in consumer products, including pesticide products, landscaping/yard products, and pet care products (NLM 2013). It is listed as a Proposition 65 carcinogen (California OEHHA 2014).	NHANES and others have measured urinary 3-phenoxybenzoicacid (3 PBA), a metabolite of fenvalerate and other pyrethroid pesticides, in the urine of the general population and people who are occupationally exposed, and methods have been developed to measure fenvalerate and esfenvalerate in urine. In 99-00 and 01-02, NHANES measured urinary 3 PBA via organic liquid extraction and LC-MS/MS in 75% of samples, with LOD 0.1 µg/L (CDC 2009; Riederer et al. 2008). NHANES, 2003-2004 also measured 3 PBA, but withdrew the data "due to unacceptable measurement variance at or near the LOD" (CDC 2011). Other studies of 3-PBA levels are reviewed in the 3rd NHANES exposure report (CDC 2005) and by Egeghy (2011). Ramesh and Ravi (2004) tested 73 blood samples from occupationally exposed people via negative ion channel ionization GC-MS for fenvalerate and other pyrethroid pesticides, and did not detect any above the LOD of 0.2 pg/mL. Loper and Anderson (2003) described liquid chromatography with diode array detection for the detection of fenvalerate and other pyrethroid and pyrethrin pesticides in 5 mL of urine, with LODs between 0.002 and 0.04 µg/mL. Shan et al. (1999) described ELISA with SPE to measure PBA and esfenvalerate in urine, with LLOQ 1 µg/L.
4680-78-8	Guinea Green B	Miscellaneous	Guinea Green B is used to dye wool, silk, leather, paper, and wood. In the past, it was used as a food, drug and cosmetic dye to color gelatin desserts, frozen desserts, sweets and confections which did not contain fats and oils, bakery products and cereals, and drug capsules. However, its use as color additive for foods, drugs and cosmetics was forbidden in the USA in late 1966 and its use as a food additive was forbidden in Japan in 1967. It is considered to be unsafe for use in food throughout the world. In Western Europe, Guinea Green B can provisionally be used in cosmetics that do not come into contact with mucous membranes; and in Japan, it is used in externally applied cosmetics (NLM 2011).	No studies were found measuring biomarkers of exposure to Guinea Green B.
302-01-2	Hydrazine	Miscellaneous	The potential for exposure of the general population to hydrazine is low, but it may occur through inhalation of cigarette smoke or ingestion of trace amounts in processed foods (NTP 2011). Another possible exposure includes dermal contact with vapors and other products manufactured with hydrazine such as textile dyes, pharmaceuticals, and photography chemicals (NLM 2011). A major source of hydrazine to the environment is from discharge of cooling water from nuclear power facilities and, to a lesser degree, from fossil fuel-based power facilities (Environment Canada 2011), and it has been detected at low levels in wastewater (IARC 1999k). It is listed as a Proposition 65 carcinogen (California OEHHA 2014). REACH SVHC Candidate List, with use in fuel, propellant, gas, corrosion inhibitors, and and in polymerisation reactions (ECHA 2013).	No occupational or general-population studies could be found for hydrazine, though there was one study of blood hydrazine levels in humans after administration of specific drugs, as well as multiple methods-development studies on blood and urine. Blair et al. (1985), using GC-MS, detected hydrazine in plasma from 8/8 volunteers taking isoniazid and 8/14 patients taking hydralazine chronically. Kirchherr et al. (1993) used HPLC to test for hydrazine in plasma and serum and obtained LOD 1 ng/ml, and LLOQ 5ng/ml. Von Sassen et al. (1985) used HPLC to detect the metabolites acetylhydrazine and diacetylhydrazine in plasma, with DLs of 0.5 nmol/ml and 1 nmol/ml respectively. Von Sasssen et al. (1985) also used HPLC to analyze urine, with DLs of 10 nmol/ml for acetylhydrazine and 20 nmol/ml for diacetylhydrazine. Seifart et al. (1995) used GCMS to detect hydrazine in unspecified (in abstract) “biological fluids,” with LLOQ 10ng/ml.
78-79-5	Isoprene	Miscellaneous	Isoprene is formed endogenously in humans, emitted from plants and trees, and is widely present in the environment at low concentrations. Sources of anthropogenic releases of isoprene to the atmosphere include ethylene production by petroleum processing, wood pulping, oil fires, wood-burning stoves and fireplaces, other biomass combustion, tobacco smoke, gasoline, and exhaust of turbines and automobiles (NTP 2011). It is listed as a Proposition 65 carcinogen (California OEHHA 2014).	Isoprene is believed to be involved in the cholesterol synthesis pathway (Stone et al. 1993) and is associated with factors including heart rate, sleep or wakefulness, recent exercise, and age (Cailleux et al. 1993; Kushch et al. 2008; Turner et al. 2006). It is therefore unlikely to be a good marker of exposure to products of combustion, though methods exist to study levels in blood and exhaled breath. Concentrations in blood samples from a general population range from 15 to 70 nmol/l with mean of 37 nmol/l and SD of 25 nmol/l (Cailleux et al. 1992). Csanady et al. (2001) predict that the blood concentration in nonexposed humans should be about 9.5 nmol/l. In the general population, concentration in exhaled breath was found to range from 0-474 ppb with a mean of 118 ppb and a SD of 68 ppb (Turner et al. 2006). For blood, the most sensitive method found was SPME/GC-MS, with LOD between 0.02 and 0.1 nmol/l (Miekisch et al. 2001). The most sensitive method for breath was proton-ion-transfer-MS (PIT-MS) with an LOD between 0.05 and 0.3 ppb (Mieth et al. 2009).

CAS	Name	Chemical group	Exposure summary	Biomarker summary
129-73-7	Leucomalachite green	Miscellaneous	The general public may become exposed to leucomalachite green through the consumption of fish treated with this compound, which is also used as an anti-bacterial agent in aquaculture (NTP 2004)). This compound is also used in processing malachite green, used for dyeing silk, wool, jute, cotton, and leather (NLM 2011). Also see malachite green.	No studies were found measuring malachite green or leucomalachite green biomarkers in humans. Several LC-based methods have been used on tissues of fish, shrimp, and other aquatic organisms (Andersen et al. 2005; Long et al. 2008; Mitrowska et al. 2005; Turnipseed et al. 2005; Wu et al. 2007; Zhu et al. 2007)., the most sensitive having a detection limit of 0.25 ng/g (Andersen et al. 2005).
2437-29-8	Malachite green	Miscellaneous	The general public may become exposed to malachite green through the consumption of fish treated with this compound, which is also used as an anti-bacterial agent in aquaculture (NTP 2004). Also used for dyeing silk, wool, jute, cotton, and leather (NLM 2011) See entry for leucomalachite green.	No studies were found measuring malachite green or leucomalachite green biomarkers in humans. Several LC-based methods have been used on tissues of fish, shrimp, and other aquatic organisms (Andersen et al. 2005; Long et al. 2008; Mitrowska et al. 2005; Turnipseed et al. 2005; Wu et al. 2007; Zhu et al. 2007), the most sensitive having a detection limit of 0.25 ng/g (Andersen et al. 2005).
93-15-2	Methyleugenol	Miscellaneous	Methyleugenol is a naturally occurring substance, present in many essential oils, including rose, pimento, basil, hyacinth, citronella, anise, nutmeg, mace, cinnamon leaves, pixuri seeds, and laurel fruits and leaves. It is a registered food additive used in commercial products as a flavorant and a fragrance at small concentrations in jellies, baked goods, nonalcoholic beverages, chewing gum, candy, pudding, relish, and ice cream (NTP 2011; US FDA 2013). It is listed as a Proposition 65 carcinogen (California OEHHA 2014).	Two general-population studies were found measuring methyleugenol (ME) levels in blood. One study described detection in rat liver. Barr et al. (2000) described SPE-GC-MS, with a DL of 3.1 pg/g wet weight. Testing a subset of the US NHANES population, Barr et al. (2000) found ME in 98% of serum samples, with a mean concentration of 24 pg/g, and a maximum of 390 pg/g. Schecter et al. (2004) found that ME levels in blood increased from a mean of 16.2 pg/g fasting to a mean of 63.9 pg/g 15 minutes after a high-ME snack. Gardner (1996) described ELISA and immunoblotting to detect ME in rat liver.
98-95-3	Nitrobenzene	Miscellaneous	Nitrobenzene has been detected in surface and groundwater (IARC 1996a). The general public may be exposed to nitrobenzene in the environment through inhalation of ambient air, ingestion of water, or dermal contact with products or water containing nitrobenzene. Nitrobenzene is found in soaps and shoe and metal polishes and is used as a preservative in spray paints, constituent of floor polishes, substitute for almond essence, and in the perfume industry (NLM 2011; NTP 2011). It is listed as a Proposition 65 carcinogen (California OEHHA 2014).	NHANES has measured nitrobenzene in blood samples from the general population, and other studies have measured nitrobenzene exposure in blood of occupationally or otherwise exposed humans. Methods also exist for determination of nitrobenzene in urine and bone marrow. NHANES has used HS-SPME-GC-MS on 3 mL (minimum) to 10 mL (optimal) whole blood, but less than 5% of the population had levels above the LOD (0.3 ng/mL) (Blount et al. 2006; CDC 2008b, 2009). Thier et al. (2001) measured aniline, benzidine, and 4-aminodiphenyl adducts of hemoglobin and human serum albumin in 80 male employees of a nitrobenzene reduction plant, finding a large difference between smokers and non-smokers in hemoglobin-ADP adducts but not in aniline adducts, which they concluded were dominated by occupational exposure. Martinez et al. (2003) used GC-FID and GC-MS on the blood of a patient suffering from symptoms of poisoning, and detected 3.2 µg nitrobenzene/mL whole blood 48 hours after the eventually fatal dose. Dangwal and Kadam (1980) used microdiffusion to measure nitrobenzene in urine, with LOD 0.2 mg/L. Chen et al. (2004) detected nitrobeneze and other nitro metabolites of benzene in the bone marrow of mice that had been treated with benzene 1 hour before.
75-52-5	Nitromethane	Miscellaneous	The general population may be exposed by inhalation of nitromethane in motor vehicle exhaust and cigarette smoke (NTP 2011). Exposures may also occur from the use of solvents, aeresol propellants, and fuels containing nitromethane (IARC 2000b). It is found in craft model fuels (NLM 2013) and is listed as an ingredient in manicuring preparations and rubber adhesives. Nitromethane has been detected in air, surface water, and drinking water. It is listed as a Proposition 65 carcinogen (California OEHHA 2014).	One study has measured nitromethane in blood from the general population. Alwis et al. (2008) used SPME-GC-HRMS with LOD 0.01 µg/L to measure nitromethane in the blood of 632 people with no known occupational exposure. Concentrations ranged from 0.28-3.97 µg/L, with a median of 0.66 µg/L. The authors of the study point out that nitromethane in the blood can indicate exposure to nitromethane itself or to halonitromethanes, and that it can be formed from peroxynitrite. Other studies (e.g. Mullins and Hammett-Stabler 1998) have noted that high blood levels of nitromethane can interfere with creatinine measurements.

CAS	Name	Chemical group	Exposure summary	Biomarker summary
924-16-3	n-Nitroso-di-n-butylamine	Miscellaneous	Estimates indicate that air, diet, and smoking contribute to potential human exposure to n-nitroso-di-n-butylamine at levels of a few µg per day. This compound and other n-nitrosamines are frequently produced during rubber processing and may be present as contaminants in the final rubber product. Nitrosamines present in pacifiers and baby bottle nipples can migrate from the pacifier or nipple into saliva, which could result in ingestion of nitrosamines (IARC 1993c; NTP 2005). Nitrosamines are found in cosmetics, lotions, shampoos, cutting fluids, certain pesticides, antifreeze, cooked fish, pork luncheon meat, the interior of new cars, cigarette smoke, and an aqueous rubber extract; nitrosamines are formed within these products by reactions of precursors or introduced through the use of contaminated raw materials (NLM 2011; NTP 2011). It is listed as a Proposition 65 carcinogen (California OEHHA 2014).	No studies were found using biomarkers for n-nitroso-di-n-butylamine
88-72-2	o-Nitrotoluene	Miscellaneous	The general population may be exposed to o-nitrotoluene as a result of its occurrence in the environment from inadvertent spills of o-nitrotoluene or chemical mixtures containing o-nitrotoluene, emissions directly into the environment, or breakdown products of dinitrotoluenes (DNT) and trinitrotoluenes (TNT) (NTP 2011). Exposure to nitrotoluenes can also occur during their production and use, although few data are available. Consumer products that may contain this chemical include: art materials, putty, glazing, wood preservatives and brush cleaners (US EPA 2010b). It has been detected in effluents from the manufacture or use of nitrotoluenes and in surface and groundwater (IARC 1996b) and has been detected in U.S. air and water (NTP 2011). It is listed as a Proposition 65 carcinogen (California OEHHA 2014).	Two studies were found measuring exposure to o-nitrotoluene (2NT) in the blood or urine of exposed workers. Jones et al. (2005) measured hemoglobin adducts in the blood of Chinese workers exposed to nitrotoluenes, finding that the Hb-2NT adduct was the most abundant mononitrotoluene adduct. Jones et al. (2005) found nitrobenzoic acid metabolites of 2NT in the urine of 96% of exposed workers.
1120-71-4	Propane sultone	Miscellaneous	Consumers are potentially exposed to residues of propane sultone when using detergents, corrosion inhibitors, and other products manufactured from 1,3-propane sultone (NTP 2011). It is listed as a Proposition 65 carcinogen (California OEHHA 2014).	No studies were found measuring biomarkers of exposure to propane sultone
95-06-7	Sulfallate	Miscellaneous	Sulfallate was used as an herbicide until the early 1990s and is no longer used in the United States. In the past, the general population may have been exposed to sulfallate through ingestion of residues in food crops (NTP 2011). It is listed as a Proposition 65 carcinogen (California OEHHA 2014).	No studies were found using biomarkers of exposure to sulfallate.
51-79-6	Urethane	Miscellaneous	The general population may be exposed to urethane via ingestion of fermented foods and alcoholic beverages. Urethane is used as a solvent for organic materials and co-solvent in the manufacture of pesticides, fumigants, and cosmetics. It has been found in foods made by a fermentation process, including ale, beer, bread, wine, soy sauce, yogurt, and olives (NLM 2011; NTP 2011). It is listed as a Proposition 65 carcinogen (California OEHHA 2014).	No studies were found using specific biomarkers to measure exposure to urethane. Sun et al. (2006) and Bartsch et al. (2000) describe measurement of etheno-DNA adducts in urine as markers of exposure to urethane, vinyl chloride, or endogenous oxidative stress processes. Bartsch et al. (2000) reviews measurement of etheno-DNA adducts in other organs.

CAS	Name	Chemical group	Exposure summary	Biomarker summary
NA	X-rays, gamma rays (ionizing radiation)	Miscellaneous	The greatest exposure of the general population to X-rays and gamma rays comes from natural terrestrial radiation. The next most significant source is the use of X-rays and radiopharmaceuticals in various medical diagnostic and therapeutic procedures. Exposures may also occur from the generation of energy by nuclear reactors or accidents at these facilities. Exposures from the atmospheric testing of nuclear weapons have diminished (NTP 2011).	No methods are currently available that detect internal ionizing radiation exposure retrospectively, although research is underway to develop a method that can detect recent exposure by evaluating expression of DNA-repair genes (Budworth et al. 2012).

BMI = body mass index; CDC = Centers for Disease Control and Prevention; EI = electron impact; ELISA = enzyme linked immunosorbent assay; EPA = Environmental Protection Agency; ESI = electrospray ionization; ETS = environmental tobacco smoke; FD = fluorescence detection; FI-CL = flow injection chemiluminescence; FID = flame ionization detection; GC = gas chromatography; HF = hollow fiber; HPLC = high-performance liquid chromatography; HR MS = high-resolution mass spectrometry; HS = headspace; IAC = immunoaffinity chromatography; IARC = International Agency for Research on Cancer; IOM = Institute of Medicine; LC = liquid chromatography; LLE = liquid-liquid extraction; LLOQ = lower limit of quantification; LOD = limit of detection; MS = mass spectrometry; MS/MS = tandem MS; NCI = negative-ion chemical ionization; NHANES = National Health and Nutrition Examination Survey; NIOSH = National Institute for Occupational Safety and Health; NTP = National Toxicology Program; PAH = polycyclic aromatic hydrocarbon; PFC = perfluorinated chemical; PIT MS = proton ion transfer MS; RIA = radio immunoassay; Rp= reverse phase; SIM = selected ion monitoring; SPE = solid phase extraction; SPME = solid phase micro extraction; TLC = thin layer chromatography; UPLC = ultra high-performance liquid chromatography; UV = ultraviolet detection; VOC = volatile organic compound.

Table S2. Guide to breast cancer cohort studies (studies assessing breast cancer incidence, recurrence, or survival).

Study name	Institution (PI)	Funder(s)	Study population	Study period	Measurements	Health outcomes
Avon Longitudinal Study of Parents and Children (ALSPAC; Children of the 90s)	University of Bristol (George Davey Smith)	UK Medical Research Council, Wellcome Trust, University of Bristol	13,761 pregnant women near Avon, England recruited in 1991-92, along with their children and partners.	Ongoing research: 1991-present	Measurements include blood, urine, hair, nail, saliva, placenta samples; lymphoblastoid cell lines; DNA; questionnaire and clinical data.	Outcomes include development and puberty outcomes in children and breast and other cancer in mothers.
Agricultural Health Study	NCI (Michael Alvanaja and Laura Beane Freeman), NIEHS (Dale Sandler and Jane Hoppin), US EPA (Kent Thomas), NIOSH (Cynthia Hines)	NCI, NIEHS, NIOSH, US EPA	89,000 private pesticide applicators (farmers) and their spouses in Iowa and North Carolina, as well as commercial pesticide applicators in Iowa.	Ongoing research: 1993-present	Measurements include surveys of smoking, drinking and diet; buccal rinse samples; information on occupational practices; lifestyle factors; pesticide exposure; family history of cancer; reproductive history; agricultural exposure; and general health.	Outcomes include cancer (breast, leukemia, myeloma, non-Hodgkin's lymphoma, melanoma of the skin, lung, colon, rectal, pancreas, bladder, and prostate), asthma, neurologic disease, injury, mortality, and adverse reproductive effects.
Arteriosclerosis Risk in Communities (ARIC)	Johns Hopkins (Elizabeth A. Platz), University of North Carolina (David Couper)	NIH	15,792 participants (55% female, 27% African-American) aged 45-64 in 1987 recruited from North Carolina, Mississippi, Minneapolis, and Maryland; 3,145 participants diagnosed with cancer by 2006.	Ongoing research: 1987-present	Measurements include blood and urine samples, clinical exams, food frequency questionnaires; tissue blocks to be collected from cancer cases.	This cohort was originally established to study arteriosclerosis, but has data on cancer incidence and recently received funding to collect and analyze more detailed information about cancer diagnoses and treatments.
Black Women's Health Study	Slone Epidemiology Center at Boston University (Lynn Rosenberg and Julie Palmer), Lombardi Cancer Center at Georgetown University (Lucile Adams-Campbell)	NIH	59,000 black women in US.	Ongoing research: 1995-present (funded through 2014)	Measurements include questionnaires every two years, medical records (if disease of interest), cancer registry data. Validation studies: dietary study (400), physical activity study (100+), and buccal cell samples (26,800).	Outcomes include breast and other cancers, and nonmalignant conditions that disproportionately affect black women.
Breakthrough Generations Study	University of London Institute of Cancer Research (Anthony Swerdlow and Alan Ashworth)	University of London Institute of Cancer Research, Breakthrough Breast Cancer	112,049 British women, ages 16-102 at recruitment; 30% are first-degree relatives of another study member.	Ongoing research: recruitment 2003-2011, follow-up expected to continue at least through 2050	Measurements include questionnaires about current exposures, historical exposures, and anthropomorphic measurements every 2 1/2 years, blood samples at enrollment (follow-up blood samples planned).	Outcomes include breast and other cancer incidence and mortality, as well as details including histology, grade information, and hormone receptor information. Information on other illnesses and cause of death also collected.
Breast Cancer Family Registry	National Cancer Institute (Sheri Dixon Schully), Columbia University (Mary Beth Terry), Northern California Cancer Center (Esther John), Fox Chase Cancer Center (Mary Daly), University of Utah (Saundra Buys), University of Melbourne (John Hopper), Cancer Care Ontario (Irene Andrulis)	NCI	40,000 participants from more than 13,000 families, enrolled from population-based case families, population-based control families, and clinic-based families.	Ongoing research: 1996-present	Measurements include family history information, epidemiological and clinical data, and biological specimens (blood and/or buccal samples and tumor tissue).	Outcomes include breast cancer.

Study name	Institution (PI)	Funder(s)	Study population	Study period	Measurements	Health outcomes
California Flight Attendants Study	Northern California Cancer Center (Peggy Reynolds)	California Breast Cancer Research Program	Residents of CA and members of the Association of Flight Attendants (AFA).	1988-1995	Measurements include work history information and matching of AFA members with the California Cancer Registry.	Outcomes include breast and other cancers.
California Teachers Study	City of Hope Comprehensive Cancer Center (Leslie Bernstein), California Department of Health Services, Northern California Cancer Center, UC Irvine, and USC	NIH, NCI, others	133,479 current and former public school teachers or administrators who participate in the California State Teachers Retirement System (STRS); ages range from 22 to 104 (mode 50-60). >12,000 cancer diagnoses (4,576 invasive breast cancer diagnoses, 1,091 in situ breast cancer diagnoses).	Ongoing research: 1995-present (funded through 2016)	Measurements include questionnaires, cancer registry data, mortality data, blood samples for 1,800 breast cancer cases and 2,600 controls, 3,000 buccal cell samples, 400 toenail samples, and a measurement substudy of 328 (304 with urine samples).	Outcomes include breast and other cancers, and general health.
Canadian National Breast Screening Study (CNBSS)	University of Toronto (Anthony Miller), Albert Einstein College of Medicine (Tom Rohan)	Canadian Breast Cancer Foundation	89,835 women living in 12 Canadian cities who were between 40-59 years at enrollment (1982-1987). 50,430 women age 40-49 and 39,405 age 50-59. 56,837 completed a self administered dietary questionnaire.	1980-1987 (recruitment); continued passive follow-up using national databases	Measurements include breast physical exam, mammography, pathology results, questionnaire data, food frequency questionnaire, demographic information, breast and other cancer incidence and cohort mortality.	Outcomes include breast, lung, and other cancers.
Canadian Partnership for Tomorrow	Various	Canadian Partnership Against Cancer, regional institutions	Over 250,000 Canadians in five regions of Canada.	Ongoing research: 2001-present	Measurements include blood, saliva, varied questionnaire and clinical data depending on sub study.	Outcomes include breast and other cancers.
Cancer Prevention Study 3 (CPS 3)	American Cancer Society (Alpa Patel)	American Cancer Society	Expected size of 500,000 men and women age 30-65 with no personal history of cancer; goal of at least 25% minority participation.	New research (enrollment ongoing, 20+ years follow-up planned)	Measurements include baseline survey, waist measurement and blood sample. Questionnaire data will be collected every 2-3 years. Participants reporting cancer will be verified through medical records or cancer registry linkage.	Outcomes include all causes of mortality and cancer incidence.
Cancer Prevention Study II (CPS II)	American Cancer Society (Susan Gapstur)	American Cancer Society	1.2 million American men and women enrolled in 1982.	Ongoing research: 1982-present	Measurements include the 1982 baseline questionnaire; additional questionnaires in 1992, 1997, and every two years thereafter (CPS-II Nutrition Cohort); blood samples (39,380) and buccal cells (from an additional 67,000).	Outcomes include mortality from breast and other cancers (1982-present). Incidence of breast cancer and other cancers since 1992 for CPS-II Nutrition Cohort (~98,000 women, ~84,000 men).

Study name	Institution (PI)	Funder(s)	Study population	Study period	Measurements	Health outcomes
Child Health and Development Study (CHDS)	Public Health Institute (Barbara Cohn)	NIH	Women living near Oakland, CA who were under the care of Kaiser Permanente Health Plan were enrolled when they first found out they were pregnant. 15,528 families, 20,530 pregnancies, 20,754 babies and 19,044 live births enrolled from 1959 to 1967.	Ongoing research: 1959-present	Measurements include prenatal maternal interviews, clinical assessments, biological specimens, placental pathologies, medical histories (physical and mental), developmental, emotional and behavioral assessments, and reproductive outcomes.	Outcomes include pregnancy and infant health and development, early experiences and disease/condition onset, and cancer and mortality in fathers, mothers and now-adult children.
CLUE I & II	Bloomberg School of Public Health, John Hopkins University (Kala Visvanathan)	Johns Hopkins University Bloomberg School of Public Health and NCI Intramural Program	25,802 participants in 1974, 32,898 participants in 1989 (overlap of 8395); over half female. Both samples have "reasonable representation of the general county population."	1974 to 2007 (some information from 1963 and 1965)	Measurements include blood sample, blood pressure and questionnaires.	Outcomes include breast and other cancers, CVD, and stroke.
Columbia, Missouri Serum Bank	NCI (Louise Brinton)	NCI	7,224 women living in and around Columbia, MO.	Enrollment in 1977-1987	Measurements include hormones measured in serum collected from 1977-1987 and follow-up questionnaires.	Outcomes of interest include breast cancer.
DES Combined Cohort Follow-up Study	Various researchers	NCI, CDC	Multiple cohort studies combined; NCI still follows approximately 15,000 members of the combined cohort study.	Ongoing research: 1971-present (individual cohorts); 1991-present (combined cohorts)	Measurements include questionnaires about reproductive health.	Outcomes include breast and other cancers and reproductive effects.
European Prospective Investigation into Cancer and Nutrition (EPIC)	IARC, WHO (Elio Riboli)	European Commission "Europe Against Cancer" Programme	520,000 men and women in 10 European countries (Denmark, France, Germany, Greece, Italy, The Netherlands, Norway, Spain, Sweden and UK); participants mostly age 20 or older at enrollment (1993-1999).	Ongoing research: 1992-present	Measurements include a baseline survey; anthropometric assessment (e.g. height, weight, hip and waist measurements); and blood samples.	Outcomes include chronic diseases, including breast and other cancers.
Framingham Heart Study	NHLBI (Daniel Levy and Christopher O'Donnell) and Boston University (Philip Wolf, William Kannel, Emelia Benjamin, Joanne Murabito, and Ralph D'Agostino)	NHLBI	Original cohort: 5,209 men and women from Framingham, MA ages 30 to 62 in 1948. Offspring cohort: 5,124 adult children of original cohort and their spouses, enrolled in 1971. Generation III cohort: 4,095 adult grandchildren. Omni Cohort 1: 506 minority men and women enrolled in 1994 to increase ethnic diversity of participants. Omni Cohort 2: 402 participants added to the original omni cohort.	Ongoing research: 1948-present	Measurements include medical histories (including hormone use), physical exams, and laboratory tests (plasma) every 2 years.	Outcomes include CVD and other chronic outcomes, including breast cancer.

Study name	Institution (PI)	Funder(s)	Study population	Study period	Measurements	Health outcomes
General COhort of Adults in NORway (CONOR)	Norwegian University of Science and Technology (Lars J. Vatten), The Norwegian Cancer Registry (Giske Ursin)	Norwegian Institute of Public Health, University of Tromsø, Norwegian University of Science and Technology in Trondheim, University of Bergen, and University of Oslo	185,000 participants in different parts of Norway.	Ongoing reseach: 1993 to present	Measurements include blood samples, extracted DNA, cancer data and smoking, body mass and physical activity data.	CONOR is a collaboration studying rare diseases and cancer; member studies may include other outcomes.
Great Lakes Human Health Effects Research Program (GLHHERP)	ATSDR (Heraline Hicks), multiple funded organizations	ATSDR	People residing in the Great Lakes basin (multiple cohorts, multiple studies).	Ongoing research: 1992-present	Measurements include surveys, gene function and biomarkers measured in blood, and morbidity/mortality data (depends on specific research project).	Outcomes include health effects related to fish consumption (depends on specific research project).
Hormones and Diet in the Etiology of Breast Cancer Risk (ORDET)	Instituto Nazionale Per lo Studio e la Cura dei Tumori (Franco Berrino, Paola Muti, Vittorio Krogh)	NCI, US Army Medical Research and Material Command	10,786 Italian women without any history of cancer or hormone therapy, age 35-69 at enrollment; about 130 breast cancer cases with blood samples.	1987-2003 (enrollment 1987-1992)	Measurements include questionnaires, anthropometric variables, blood samples, and urine samples.	Outcomes include incident breast cancer.
Iowa Women's Health Study	University of Minnesota (Kim Robien)	NCI	41,836 Iowa women, age 55-69 in 1986; >2,900 incident breast cancers by 2007.	Ongoing research: 1985-present	Measurements include surveys (one at baseline and 5 follow-ups), DNA (limited number of participants), and water samples.	Outcomes include CVD, chronic disease, and cancer, including breast cancer.
Janus Serum Bank	Cancer Registry of Norway, Institute of Population-based Cancer Research (Hilde Langseth)	Cancer Registry of Norway	317,000 Norwegians comprising blood donors in Oslo area and participants in past studies; 52,500 donors diagnosed with cancer by 2009.	Ongoing research: Specimens collected 1972-2004, with ongoing collection from earlier donors who have developed cancer since donating.	Measurements include blood samples.	Outcomes include cancers, recorded in Norwegian Cancer Registry.
Japan Public Health Center-Based Prospective Study (JPHC)	Research Center for Cancer Prevention and Screening, National Cancer Center (Shoichiro Tsugane)	Grant-in-Aid for Cancer Research from the Ministry of Health, Labor and Welfare, Japan	140,420 residents of 29 municipalities within 11 public health center (PHC) areas nationwide.	Ongoing research: 1990-present	Measurements include three surveys of lifestyle habits to date, at five-year intervals, blood samples and health check-up data from 60,000 people.	Outcomes include mortality, incidence of cancer, cerebrovascular disease and ischemic heart disease, diabetes mellitus, periodontal disease, age-related cataract, vertebral fracture, and other chronic diseases.
Kaiser Research Program on Genes, the Environment and Health (RPGEH)	Kaiser Permanente (Cathy Schaefer)	Community Benefit Program of Kaiser Permanente, Robert Wood Johnson Foundation, Wayne and Gladys Valley Foundation, Ellison Medical Foundation	500,000 Northern California Kaiser Permanente members (men and women). Current enrollment is 200,000.	Ongoing research: 2007-present	Measurements include medical, lifestyle, demographic, environmental and, in some cases, genetic information from saliva and blood samples.	Outcomes include CVD, cancer, diabetes, high blood pressure, Alzheimer’s disease, asthma and many others.

Study name	Institution (PI)	Funder(s)	Study population	Study period	Measurements	Health outcomes
Kathleen Cuningham Foundation Consortium for Research into Familial Aspects of Breast Cancer (kConFab) biospecimen and data resource	Various	Australian National Health and Medical Research Council, State Cancer Councils, National Breast Cancer Foundation	Members of 1,600 families with multiple breast cancer cases.	Ongoing research: 1997-present	Measurements include genetic, epidemiological, medical, psychosocial and clinical follow-up data, biospecimens.	Outcomes include familial breast, ovarian, prostate and pancreatic cancer.
Love/Avon Army of Women	Dr. Susan Love Research Foundation	Avon Foundation	1 million women (~373,000 as of July 2013).	Ongoing research: 2008-present	Measurements vary depending on research project.	Outcomes vary depending on research project.
Massachusetts Women's Health Study	New England Research Institutes (Sonja McKinlay)	NIH/NIA	8,000+ MA women born between 1926 and 1936 (age 45-54); follow up of all 2,572 premenopausal women.	1982-1987 and 1986-1996 for a sub-cohort with physiological measures	Measurements include cross-sectional survey (8,000+), telephone interviews every 9 months for 54 months (2,572) and then annually (~450).	Outcomes include timing of menopause and potentially menopause-related experiences.
Mayo Mammography Health Study	Mayo Clinic College of Medicine (Celine M. Vachon)	Mayo Clinic Cancer Center	19,924 women ages 35 and over, living in Minnesota, Iowa, and Wisconsin, without a history of breast cancer.	Ongoing research: 2003-present	Measurements include mammograms, self-administered questionnaire, and blood samples from over half of participants.	Outcomes include breast and other cancers.
Melbourne Collaborative Cohort Study (MCCS, a.k.a. Health 2020)	Cancer Council Victoria (Graham Giles)	Cancer Council Victoria	41,500 people (24,500 women and 17,000 men) age 40-69; southern European migrants (25% of participants) were deliberately over-sampled to increase the range of lifestyle exposures and genetic variation.	Ongoing research: 1990-present	Measurements include baseline interview, physical measurements, food frequency questionnaire and blood sample; follow-up questionnaire administered 3-4 years after baseline; second physical follow-up administered 12-14 years after baseline.	Outcomes include cancer and all-cause mortality.
MESA (Multi-Ethnic Study of Atherosclerosis; including MESA Family, MESA Air, and MESA Lung)	University of Washington - Coordinating Center (Richard Kronmal), Columbia, Johns Hopkins, Wake Forest, Northwestern, UCLA, University of Minnesota	NHLBI	6,814 multi-ethnic (White, Black, Hispanic, Chinese) men and women ages 45-84 from six field centers across the US.	2000-2009	Measurements include clinical examinations, blood samples, urine samples, dietary surveys, ECGs, and DNA.	Outcomes include CVD.
Michigan Long-Term PBB Study	Michigan Department of Community Health, Emory University (Michele Marcus)	CDC, NIH, and US EPA	4,000 Michigan residents accidentally exposed to PBB in 1973, including 1,900 women and their daughters.	1977 (cohort identified)-2011; 1996-1999 additional study on EDCs conducted	Measurements include blood samples, health outcomes, and interviews.	Outcomes include effects of PBB on exposed women's reproductive health and chronic diseases including breast cancer.
Multiethnic/Minority Cohort Study of Diet and Cancer	University of Hawaii-Manoa (Laurence Kolonel)	NCI	215,000 men and women primarily of African-American, Japanese, Latino, Native Hawaiian, and Caucasian origin.	1993-2008	Measurements include a baseline survey, a subset of dietary surveys, and blood and urine samples for a subset (70,000).	Outcomes include breast and other cancers.
New York University Women's Health Study (NYUWHS)	New York University School of Medicine (Anne Zeleniuch-Jacquotte)	NCI	14,274 women age 35-65 at enrollment (1985-1991).	Ongoing research: 1985-present	Measurements include baseline and follow-up questionnaires with a focus on environmental factors and a blood sample at recruitment.	Outcomes include breast cancer and other chronic diseases.

Study name	Institution (PI)	Funder(s)	Study population	Study period	Measurements	Health outcomes
Nurses' Health Study I (NHS I)	Channing/HMS/ Brigham and Women's Hospital (Susan Hankinson)	NCI, NIH	122,000 female, married registered nurses, age 30-55 in 1976, who resided in the 11 most populous states.	Ongoing research: 1976-present	Measurements include surveys every two years focusing on menopausal status, smoking and hormone use; dietary surveys (1980, 1984, 1986 and every four years since); Quality of Life surveys (1992 and every four years since); toenail samples (68,000 sets in 1982-1984); and blood (33,000 samples in 1989-1990 and 18,700 samples in 2000-2001).	Outcomes include breast and other cancers, CVD, and other chronic diseases and conditions.
Nurses' Health Study II (NHS II)	HSPH (Walter Willett)	Originally NIH	116,686 female registered nurses age 25-42 in 1989.	Ongoing research: 1989-present	Measurements include surveys every two years focusing on oral contraceptive use, diet, smoking, pregnancies, and menopausal status; food frequency surveys start in 1991 and continue every four years since; Quality of Life surveys in 1993 and 1997; and blood and urine samples (30,000) in late 1990s.	Outcomes include breast cancer and other diseases.
Nurses' Health Study III (NHS III)	Channing/HMS/HSPH (Walter Willett)	Internal funding	Female nurses (including RNs, NPs, and LPN/LVNs) and nursing students in the US and Canada (age 20-46). Goal of 100,000 participants.	New research (currently in recruitment/pilot study phase)	Measurements include web-based surveys every six months, and diet and lifestyle information for sub-study of women who become pregnant.	Outcomes include breast cancer and general chronic diseases.
Seventh-day Adventist Cohort Study: Cancer Epidemiology in Adventists - A Low Risk Group	Loma Linda University School of Health Research (Gary Fraser)	Loma Linda University, World Cancer Research Fund	71,000 white and 25,000 black adult Seventh-day Adventists in the US.	Ongoing research: 2002-present	Measurements include questionnaires with special attention paid to diet. Some blood, urine and subcutaneous fat samples.	Outcomes include cancers of the breast, prostate, and colon.
Seveso Women's Health Study	UC Berkeley (Brenda Eskenazi)	NIEHS	Women age 0-40 in 1976 who lived in Zones A or B during the Seveso Plant Explosion.	Ongoing research: 1996-present	Measurements include TCDD levels in serum, interviews focusing on reproductive history, gynecological examinations, bone density exams (subset), and clinical chemistries, including thyroid.	Outcomes include endometriosis, menstruation, menarche, menopause, fetal outcomes, breast cancer incidence, uterine function, ovarian function, diabetes and metabolic syndrome, bone density, and effects on women and neonatal thyroid hormones.
Shanghai Women's Health Study (SWHS)	Vanderbilt University (Wei Zheng)	NCI	74,942 Chinese women who were between ages 40 to 70 years at enrollment (1997-2000) and lived in urban Shanghai.	1997-2000	Measurements include surveys and biological samples (from 87.5% of participants).	Outcomes include breast and other cancers.

Study name	Institution (PI)	Funder(s)	Study population	Study period	Measurements	Health outcomes
Singapore Chinese Health Study	University of Minnesota (Jian-Min Yuan), National University of Singapore (Woon-Puay Koh), NIEHS (Stephanie J. London)	NCI	63,257 men and women, age 45–74, who were permanent residents or citizens of Singapore and who resided in government-built housing estates.	Ongoing research: 1993-present	Measurements include baseline survey, food frequency survey, demographics, current physical activity, reproductive history (women only), occupational exposure and medical history; and respiratory questionnaires; blood samples or buccal cell and spot urine samples (32,000); and blood, buccal, and urine samples from all incident cases of female breast and colorectal cancers beginning in April 1994.	Outcomes include respiratory effects and breast and other cancers.
Sister Study: Environmental and Genetic Risk Factors for Breast Cancer	NIEHS (Dale Sandler, Clarice Weinberg)	NIEHS, National Center on Minority Health and Health Disparities	50,000 women age 35 to 74 who have never been diagnosed with breast cancer but who have a biological sister who has been diagnosed with breast cancer.	Ongoing research: 2003-present	Measurements include baseline questionnaire data, fasting blood, first morning urine, household dust samples and toenail samples. Follow up includes health status questionnaire surveys (annual) and health, lifestyle and exposures surveys (every two to three years).	Outcomes include breast and other cancers, osteoporosis, CVD, diabetes, and/or autoimmune diseases.
Southern Community Cohort Study	Vanderbilt University (William Blot), Meharry Medical College, and IEI	NCI	86,000 residents, age 40 to 79, approximately two-thirds African American, of 12 southeastern US states.	Ongoing research: 2001-present	Measurements include baseline survey, blood samples, buccal samples and/or urine sample.	Outcomes include breast and other cancers and other common and chronic diseases.
Strong Heart Study	University of Oklahoma Health Sciences Center	NHLBI	4,500 American Indians from 13 tribes and communities in three geographic areas (AZ, OK, SD/ND).	Phase I (1989-1991); Phase II (1993-1995); Phase III (1998-1999); Phase IV (90 more families); Phase V (additional families, ongoing)	Measurements include clinical examinations during each phase including blood samples, urine samples, EKGs, and blood pressure measurements.	Outcomes include CVD.
Swedish Mammography Cohort (SMC)	National Institute of Environmental Medicine (Alicja Wolk)	National Institute of Environmental Medicine	Over 60,000 women living in two counties in central Sweden born between 1914 and 1948.	Ongoing research: 1987-present	Measurements include self-administered questionnaires, food consumption information, blood (subgroup), urine (subgroup), saliva (subgroup), and adipose tissue (subgroup).	Outcomes include chronic diseases, including breast and other cancers.
Women Physicians' Health Study	Emory University School of Medicine (Erica Frank)	Varied sources, including American Heart Association and CDC	4,501 female physicians (stratified sampling for original 10,000 selected), ages 30 to 70.	1993-1994	Measurements include questionnaires.	Outcomes include health measures and health-related activities, as well as demographics and professional characteristics of US female physicians.
Women's Health Initiative Study (WHI)	WHI (Staff; Ross Prentice)	NIH	161,808 generally healthy postmenopausal women ages 50-79 (68,132 clinical and 93,676 observational). 115,400 of these women are included in an extension study.	1991-2006; Extension Study until 2015	Measurements include a Randomized Clinical Trial (focusing on hormone therapy, diet, calcium/vitamin D), an Observational Study, a Community Prevention Study, clinical exams, blood samples, urine samples, and interviews.	Outcomes include CVD, breast and other cancers, and osteoporosis.

Study name	Institution (PI)	Funder(s)	Study population	Study period	Measurements	Health outcomes
Women's Lifestyle and Health	Department of Medical Epidemiology and Biostatistics, Tromso University, Norway (Elisabete Weiderpass Vainio)	Swedish Cancer Society	50,000 Swedish women age 30 to 49.	1991-1992, 2003-2004	Measurements include questionnaire on lifestyle factors.	Outcomes include cancer, CVD and other chronic diseases in young women.
Health, Eating, Activity, and Lifestyle Study (HEAL)	NCI (Rachel Ballard-Barbash), Fred Hutchinson Cancer Research Center (Anne McTiernan), City of Hope National Medical Center (Leslie Bernstein), New Mexico Tumor Registry (Charles Wiggins)	NCI	~1200 women with early-stage breast cancer recruited from Seattle area, New Mexico, and Southern California.	Ongoing research: 1996-present	Measurements include anthropometric measurements, hormone measurements, vitamin D, and genetic information (blood), mammographic density, and questionnaire including diet, physical activity, quality of life.	Outcomes include breast cancer recurrence and survival.
Life After Cancer Epidemiology (LACE)	Kaiser Foundation Research Institute (Bette Caan)	NCI	2,321 early stage breast cancer survivors (diagnosed 1997-2000) in Northern California or Utah, age 18-70, who had completed treatment other than adjuvant hormonal therapy and were free of recurrence.	2000-2004	Baseline data collected approximately two years post diagnosis and 5-6 years post diagnosis including annual questionnaires on demographics, medical history, anthropometry, diet, supplements, physical activity and quality of life.	Outcomes include breast cancer recurrence and mortality and overall mortality.
Pathways Study	Kaiser Foundation Research Institute (Lawrence Kushi)	NCI, DOD, American Cancer Society	Over 2,200 women who were at least 21 years old at breast cancer diagnosis with primary invasive breast cancer of any stage and no prior history of any cancer.	Ongoing research: 2006-present (recruitment 2006-2010)	Measurements include extensive baseline interview, blood and salvia samples; body measurements, self reported lifestyle updates, treatments, and outcomes every 12 to 24 months.	Outcomes include breast cancer survival, breast cancer recurrence, and response to chemotherapy.
Women's Environment, Cancer, and Radiation Epidemiology (WECARE) Study	Memorial Sloan-Kettering Cancer Center (Jonine Bernstein) and others	NIEHS	2,100 women with bilateral (700) and unilateral (1,400) breast cancer who were diagnosed prior to age 55.	Ongoing research: 2000-present	Measurements include questionnaire and blood samples for DNA analysis.	Outcomes include second (contralateral) primary breast cancer, with a focus on gene (ATM, BRCA 1/2)-environment (radiation) interactions.

Table S3. Guide to breast cancer cohort studies (studies assessing pubertal development).

Study name	Institution (PI)	Funder(s)	Study population	Study period	Measurements	Health outcomes
Johns Hopkins Collaborative Perinatal Study (JHCPS)	Johns Hopkins (Janet Hardy and Sam Shapiro), NIEHS (Matthew Longnecker, PI of DDE and PCBs Study)	NIEHS	Subset of NCCP mothers delivering at Johns Hopkins University Hospital between 1960-1965, their children until age 8, and grandchildren evaluated in 1992-1994.	1960-1994	Measurements include maternal blood samples, extensive questionnaires, language, speech, and behavior testing at 36 months, observations (in delivery room, 4 months, and 7 years), pediatric neurological exam at 12 months, psychological profiles, physical growth, and vision testing.	Outcomes include birth defects and other health endpoints to age 8.
North Carolina Infant Feeding Study/ North Carolina Menopause Study	NIEHS (Walter J. Rogan)	NIEHS	856 women enrolled during pregnancy (1978-1982), 600 children evaluated 1992-1997, and 514 women interviewed 2003-2004.	1978-2004	Measurements include PCB and DDE levels in blood and breast milk samples collected around delivery (856 women) and in blood samples collected around menopause (285 women).	Outcomes include childhood health and pubertal development of the children, and timing and other descriptors of menopause in the mothers.
The Stockholm Children Allergy and Environmental Prospective Birth Cohort Study (BAMSE)	Karolinska Institutet, Stockholm County Council, Astrid Lindgren Children's Hospital, Sachs Children's Hospital	Stockholm County Council, Vårdal Asthma and Allergy Foundation, Swedish Research Council, EU MeDALL project.	4089 children and their parents born 1994-1996 in Stockholm, Sweden.	Ongoing research: 1994-present	Measurements include blood and urine samples as well as questionnaire and clinical data.	This study focuses on allergies and asthma, but measurements include pubertal timing (age at menarche, age at voice change, Tanner stage).
Center for the Health Assessment of Mothers and Children of Salinas (CHAMACOS)	UC Berkeley (Brenda Eskenazi)	Various	536 children born in 2000-2001 followed from birth; 300 children age 9 added in 2010-2011.	Ongoing research: 1998-present (funded through 2014)	Measurements include blood, urine, breastmilk, house dust, and deciduous teeth samples, as well as evaluations of breast development in girls age 9-12.	Outcomes include birth weight, neurodevelopmental endpoints, pubertal timing, hormone function, obesity, asthma, and immune function.
Children's Health and the Environment in the Faroes (CHEF)	Harvard School of Public Health (Philippe Grandjean), Danish Institute of Public Health Department of Environmental Medicine (same), Faroese Hospital System Department of Occupational Medicine and Public Health (Pal Weihe), University of Southern Denmark	Arctic Monitoring and Assessment Program, DANCE, Danish Council for Strategic Research, Danish Medical Research Council, European Commission's Research Programme, US EPA, NIEHS	Cohort 1: 1022 children born 1986-1989; Cohort 2: 182 children born 1994-1995; Cohort 3: 656 children born in 1997-2000; Cohort 4: 148 children born 2000-2001; Cohort 5: 475 children born 2007-2009. All cohorts comprised children in the Faroe Islands and their mothers.	Ongoing research: 1986-present	Measurements include cord blood samples, multiple blood and hair samples from children, blood, hair, breast milk, and urine samples from mothers, and urine, hair, and semen samples from fathers, as well as questionnaire and health data. Biological samples have been analyzed for biomarkers of many organic chemicals and metals.	Outcomes include effects on growth and development, especially on neurobehavioral, cardiovascular, endocrine, and immunological functions. Includes data on puberty timing (Tanner staging, age at voice change).
Danish National Birth Cohort (DNBC)	Danish Ministry of Health Statens Serum Institut	Danish National Research Foundation	~97,000 children born 1997-2002 in Denmark and their mothers.	Ongoing research: 1997-present	Measurements include maternal blood samples (during pregnancy), cord blood samples, and infant blood samples, as well as questionnaire and clinical data.	Outcomes include pubertal timing (age at menarche, age at voice change, Tanner stage), allergies, birth defects, childhood cancers, and other childhood health outcomes.

Study name	Institution (PI)	Funder(s)	Study population	Study period	Measurements	Health outcomes
Duisburg Birth Cohort Study	Ruhr-Universität Bochum (Michael Wilhelm)	North Rhine-Westphalia State Agency for Nature, Environment, and Consumer Protection, Environmental Agency of the Federal Republic of Germany	234 children and their mothers in Duisburg, Germany.	Ongoing research: 2000-2011	Measurements include cord blood samples, blood and urine samples from mothers and children, and breast milk samples from mothers, as well as questionnaire and clinical data. Biological samples have been analyzed for some persistent organic pollutants, endocrine disruptors, and hormones.	Outcomes include measurements of child development, including pubertal timing and sex hormone levels.
Environmental and Genetic Determinants of Puberty	Breast Cancer and the Environment Research Program (BCERP), Mt. Sinai School of Medicine (Mary Wolff), Kaiser Permanente (Lawrence Kushi), and Cincinnati Children's Hospital Medical Center (Frank Biro)	NIEHS, NCI	>1200 girls from East Harlem, NY, SF Bay Area, and Cincinnati area; age 6-8 at first visit.	Ongoing research: 2003-present	Measurements include hormonal changes, obesity, diet, family history, psychosocial stressors, environmental exposures (measured with biomarkers and otherwise), and genetic polymorphisms.	Outcomes include breast stages, menarche, peak height velocity, adult height, and menstrual cyclicity.
German Infant Nutritional Intervention study – Plus influence of pollution and genetics on allergy development (GINIplus)	Helmholtz Zentrum München - Institute of Epidemiology I (Joachim Heinrich), University of Munich (Sibylle Koletzko), Kinderklinik und Poliklinik, Technische Universität München (Carl P. Bauer), Marien-Hospital Wesel, Department of Pediatrics (Dietrich Berdel), Institut für Umweltmedizinische Forschung Düsseldorf (Barbara Hoffmann)	Federal Ministry of Education and Research, Germany, Helmholtz Zentrum München - Institute of Epidemiology I	5,991 healthy, full-term newborns with and without family history of allergy born in Munich and Wesel, Germany, 1995-1998 (3,317 at ten-year follow-up).	Ongoing research: 1995-present	Measurements include blood and urine samples from children, as well as questionnaire, anthropometric, and clinical data.	This study is focused on allergies, but outcomes include pubertal timing (age at menarche, Tanner stage at 15).
Environment and Childhood Project (Infancia y Medio Ambiante, or INMA)	CREAL (Jordi Sunyer)	Multiple Spanish public health agencies and foundations	3,768 children and their mothers in seven areas of Spain.	Ongoing research: 1997-present (varies by cohort)	Measurements include biomarkers measured in cord blood, placenta, hair, and urine samples from children, biomarkers measured in maternal blood and urine, and questionnaires on parental occupation, diet, and lifestyle.	Outcomes include pubertal timing (assessed in ~400 children, planned for ~2000 more), growth, development, and asthma related outcomes.
Lessons in Epidemiology and Genetics of Adult Cancer from Youth (LEGACY Girls Study)	Columbia University (Mary Beth Terry), Cancer Prevention Institute of California (Esther John), Fox Chase Cancer Center (Mary Daly), University of Utah (Sandra Buys), Samuel Lunenfeld Research Institute of Mount Sinai Hospital (Irene Andrulis)	NIH	900 girls age 6-13 (449 enrolled as of October 2012) and a parent or guardian followed for up to five years; half of the girls are daughters of women enrolled in the Breast Cancer Family Registry, half have no family history of breast cancer.	New research: enrollment began 2012, five years of follow-up planned	Measurements include biomarkers and epigenetics in blood, urine, and saliva, anthropomorphic measurements, and surveys.	Outcomes include pubertal and psychosocial development.

Study name	Institution (PI)	Funder(s)	Study population	Study period	Measurements	Health outcomes
Influences of Life-style related factors on the Immune System and the development of Allergies in childhood – Plus the influence of traffic emissions and genetics (LISAplus)	Helmholtz Zentrum München - Institute of Epidemiology I (J. Heinrich)	Partly by Public Health Forschungsverbund Bayern (BMBF), IUF	1,467 newborns from Munich, 976 from Leipzig, 348 from Wesel, and 306 from Bad Honnef; enrolled 1997-1999.	Ongoing research: 1997-present	Measurements include cord blood and blood samples from children, dust and air samples, and questionnaire, anthropometric, and clinical data.	This study is focused on allergies, but outcomes include pubertal timing (age at menarche, Tanner stage at 15).
Multicentre Allergy Study (MAS)	Charité - University Medicine, Berlin (Ulrich Wahn), University Hospital Düsseldorf, St. Joseph's Hospital Freiberg, Johannes Gutenberg University Mainz, Technical University of Munich		1,314 newborns and parents enrolled in 1990 in Germany (Berlin, Munich, Freiburg, Mainz, Düsseldorf).	Ongoing research: 1990-present	Measurements include blood and urine samples, dust samples, and questionnaire and clinical data.	This study is focused on allergies, but outcomes include pubertal timing (age at menarche, Tanner stage).
Millennium Cohort Study, UK (MCS)	Institute of Education at University of London, Economic and Social Research Council (Heather Joshi)	UK Government (ESRC and others)	18,819 children and their families from across the UK.	Ongoing research: 2001-present; study team plans to follow cohort through adulthood	Measurements include surveys and basic tests to assess child's physical, cognitive, social, and emotional development.	Various health outcomes, early life dynamics.
National Children's Study	NIH (Steven Hirschfeld), NIEHS, CDC, US EPA	HHS, NIH, CDC, US EPA	Pregnant women and their partners and couples planning to become pregnant, and their children. Has a goal of studying 100,000 children and their parents from child's birth to age 21.	Ongoing research: 2007-present	Measurements include biospecimens from children and parents, indoor air, dust, soil, and drinking water samples, physical/clinical examinations, and behavioral observations.	Outcomes include general health and development.
New England NCPP (National Collaborative Perinatal Project) and follow-up New England Family Study	HMS, HSPH (Stephen Buka), Brown University	NIH and others	17,000 individuals from NCPP, followed from birth to age 40.	Ongoing research: 1959-present	Measurements include periodic physical and mental health assessments and a series of cognitive, behavioral and social tests.	Outcomes mainly include mental health disorders with developmental origins, as well as substance use, learning disabilities, attention-deficit/hyperactivity disorder (ADHD), and cardiovascular disease.
Prevention and Incidence of Asthma and Mite Allergy (PIAMA)	Utrecht University (Bert Brunekreef)	Asthma Foundation, ZONMW, MinVrom, RIVM++	>4000 pregnant women enrolled, children followed until age 8.	Ongoing research: 1996-2013	Measurements include blood and saliva samples from children and both parents, breast milk samples from mothers, and questionnaire and clinical data.	This study is focused on allergies and asthma, but outcomes include pubertal timing (age at voice change, pubertal stage).

References

Abe E, Duverneuil C, de la Grandmaison G, Alvarez JC. 2008. A fatal dichlorvos poisoning: concentrations in biological specimens. J Forensic Sci 53:997-1000.

Ahmed IS, Aboul-Einien MH. 2007. In vitro and in vivo evaluation of a fast-disintegrating lyophilized dry emulsion tablet containing griseofulvin. Eur J Pharm Sci 32:58-68.

Ahn H-S, Shin H-S. 2006. Determination of ethylene oxide-hemoglobin adduct by silylation and gas chromatography-electron impact-mass spectrometry. J Chromatogr B Analyt Technol Biomed Life Sci 843:202-208.

Airoldi L, Vineis P, Colombi A, Olgiati L, Dell'Osta C, Fanelli R, et al. 2005. 4-Aminobiphenyl-hemoglobin adducts and risk of smoking-related disease in never smokers and former smokers in the European Prospective Investigation into Cancer and Nutrition prospective study. Cancer Epidemiol Biomarkers Prev 14:2118-2124.

Akdemir C, Ulker OC, Basaran A, Ozkaya S, Karakaya A. 2010. Estimation of ochratoxin A in some Turkish populations: an analysis in urine as a simple, sensitive and reliable biomarker. Food Chem Toxicol 48:877-882.

Al Za'abi MA, Dehghanzadeh GH, Norris RL, Charles BG. 2006. A rapid and sensitive microscale HPLC method for the determination of indomethacin in plasma of premature neonates with patent ductus arteriousus. J Chromatogr B Analyt Technol Biomed Life Sci 830:364-367.

Albertini R, Clewell H, Himmelstein MW, Morinello E, Olin S, Preston J, et al. 2003. The use of non-tumor data in cancer risk assessment: reflections on butadiene, vinyl chloride, and benzene. Regul Toxicol Pharmacol 37:105-132.

Albertini RJ, Sram RJ, Vacek PM, Lynch J, Rossner P, Nicklas JA, et al. 2007. Molecular epidemiological studies in 1,3-butadiene exposed Czech workers: female-male comparisons. Chem Biol Interact 166:63-77.

Alexander J, Reistad R, Hegstad S, Frandsen H, Ingebrigtsen K, Paulsen JE, et al. 2002. Biomarkers of exposure to heterocyclic amines: approaches to improve the exposure assessment. Food Chem Toxicol 40:1131-1137.

Alwis KU, Blount BC, Silva LK, Smith MM, Loose K-H. 2008. Method for quantifying nitromethane in blood as a potential biomarker of halonitromethane exposure. Environ Sci Technol 42:2522-2527.

Alwis KU, Blount BC, Britt AS, Patel D, Ashley DL. 2012. Simultaneous analysis of 28 urinary VOC metabolites using ultra high performance liquid chromatography coupled with electrospray ionization tandem mass spectrometry (UPLC-ESI/MSMS). Anal Chim Acta 750:152-160.

Aman CS, Pastor A, Cighetti G, de la Guardia M. 2006. Development of a multianalyte method for the determination of anabolic hormones in bovine urine by isotope-dilution GC-MS/MS. Anal Bioanal Chem 386:1869-1879.

Ambrosone CB, Abrams SM, Gorlewska-Roberts K, Kadlubar FF. 2007. Hair dye use, meat intake, and tobacco exposure and presence of carcinogen-DNA adducts in exfoliated breast ductal epithelial cells. Arch Biochem Biophys 464:169-175.

Andersen WC, Roybal JE, Turnipseed SB. 2005. Liquid chromatographic determination of malachite green and leucomalachite green (LMG) residues in salmon with in situ LMG oxidation. J AOAC Int 88:1292-1298.

Arancibia V, Valderrama M, Madariaga A, Zúñiga MaC, Segura R. 2003. Extraction of nitrofurantoin and its toxic metabolite from urine by supercritical fluids. Quantitation by high performance liquid chromatography with UV detection. Talanta 61:377-383.

Arayasiri M, Mahidol C, Navasumrit P, Autrup H, Ruchirawat M. 2010. Biomonitoring of benzene and 1,3-butadiene exposure and early biological effects in traffic policemen. Sci Total Environ 408:4855-4862.

Aufrère MB, Hoener B, Vore ME. 1977. High-performance liquid-chromatographic assay for nitrofurantoin in plasma and urine. Clin Chem 23:2207-2212.

B'Hymer C, Cheever KL. 2010. Evaluation of a procedure for the simultaneous quantification of 4-ketocyclophosphamide, cyclophosphamide, and Ifosfamide in human urine. J Chromatogr Sci 48:328-333.

Bakke B, De Roos AJ, Barr DB, Stewart PA, Blair A, Freeman LB, et al. 2009. Exposure to atrazine and selected non-persistent pesticides among corn farmers during a growing season. J Expo Sci Environ Epidemiol 19:544-554.

Ball L, Jones A, Boogaard P, Will W, Aston P. 2005. Development of a competitive immunoassay for the determination of N-(2-hydroxypropyl)valine adducts in human haemoglobin and its application in biological monitoring. Biomarkers 10:127-137.

Baranczewski P, Gustafsson JA, Moller L. 2004. DNA adduct formation of 14 heterocyclic aromatic amines in mouse tissue after oral administration and characterization of the DNA adduct formed by 2-amino-9H-pyrido[2,3-b]indole (AalphaC), analysed by 32P_HPLC. Biomarkers 9:243-257.

Barr DB, Barr JR, Bailey SL, Lapeza CR, Beeson MD, Caudill SP, et al. 2000. Levels of methyleugenol in a subset of adults in the general U.S. population as determined by high resolution mass spectrometry. Environ Health Perspect 108:323-328.

Barr DB, Panuwet P, Nguyen JV, Udunka S, Needham LL. 2007. Assessing exposure to atrazine and its metabolites using biomonitoring. Environ Health Perspect 115:1474-1478.

Bartell SM, Calafat AM, Lyu C, Kato K, Ryan PB, Steenland K. 2010. Rate of decline in serum PFOA concentrations after granular activated carbon filtration at two public water systems in Ohio and West Virginia. Environ Health Perspect 118:222-228.

Bartsch H, Castegnaro M, Camus AM, Schouft A, Geneste O, Rojas M, et al. 1993. Analysis of DNA adducts in smokers' lung and urothelium by 32P-postlabelling: metabolic phenotype dependence and comparisons with other exposure markers. IARC Sci Publ:331-340.

Bartsch H, Nair J. 2000. New DNA-based biomarkers for oxidative stress and cancer chemoprevention studies. Eur J Cancer 36:1229-1234.

Benford D, Dinovi M, Setzer RW. 2010. Application of the margin-of-exposure (MoE) approach to substances in food that are genotoxic and carcinogenic e.g.: benzo[a]pyrene and polycyclic aromatic hydrocarbons. Food Chem Toxicol 48 Suppl 1:S42-48-S42-48.

Bergamini MF, Santos DP, Zanoni MV. 2010. Determination of isoniazid in human urine using screen-printed carbon electrode modified with poly-L-histidine. Bioelectrochemistry 77:133-138.

Berthet A, Bouchard M, Schupfer P, Vernez D, Danuser B, Huynh CK. 2011. Liquid chromatography-tandem mass spectrometry (LC/APCI-MS/MS) methods for the quantification of captan and folpet phthalimide metabolites in human plasma and urine. Anal Bioanal Chem 399:2243-2255.

Berthet A, Bouchard M, Danuser B. 2012a. Toxicokinetics of captan and folpet biomarkers in orally exposed volunteers. J Appl Toxicol 32:194-201.

Berthet A, Bouchard M, Vernez D. 2012b. Toxicokinetics of captan and folpet biomarkers in dermally exposed volunteers. J Appl Toxicol 32:202-209.

Bessette EE, Yasa I, Dunbar D, Wilkens LR, Le Marchand L, Turesky RJ. 2009. Biomonitoring of carcinogenic heterocyclic aromatic amines in hair: a validation study. Chem Res Toxicol 22:1454-1463.

Bessette EE, Spivack SD, Goodenough AK, Wang T, Pinto S, Kadlubar FF, et al. 2010. Identification of carcinogen DNA adducts in human saliva by linear quadrupole ion trap/multistage tandem mass spectrometry. Chem Res Toxicol.

Beyerbach A, Rothman N, Bhatnagar VK, Kashyap R, Sabbioni G. 2006. Hemoglobin adducts in workers exposed to benzidine and azo dyes. Carcinogenesis 27:1600-1606.

Birner G, Albrecht W, Neumann HG. 1990. Biomonitoring of aromatic amines. III: Hemoglobin binding of benzidine and some benzidine congeners. Arch Toxicol 64:97-102.

Bjellaas T, Olesen PT, Frandsen H, Haugen M, Stølen LH, Paulsen JE, et al. 2007a. Comparison of estimated dietary intake of acrylamide with hemoglobin adducts of acrylamide and glycidamide. Toxicol Sci 98:110-117.

Bjellaas T, Stølen LH, Haugen M, Paulsen JE, Alexander J, Lundanes E, et al. 2007b. Urinary acrylamide metabolites as biomarkers for short-term dietary exposure to acrylamide. Food Chem Toxicol 45:1020-1026.

Blair IA, Mansilla Tinoco R, Brodie MJ, Clare RA, Dollery CT, Timbrell JA, et al. 1985. Plasma hydrazine concentrations in man after isoniazid and hydralazine administration. Hum Toxicol 4:195-202.

Blair IA. 2010. Analysis of estrogens in serum and plasma from postmenopausal women: past present, and future. Steroids 75:297-306.

Blount BC, Kobelski RJ, McElprang DO, Ashley DL, Morrow JC, Chambers DM, et al. 2006. Quantification of 31 volatile organic compounds in whole blood using solid-phase microextraction and gas chromatography-mass spectrometry. J Chromatogr B Analyt Technol Biomed Life Sci 832:292-301.

Blount BC, McElprang DO, Chambers DM, Waterhouse MG, Squibb KS, Lakind JS. 2010. Methodology for collecting, storing, and analyzing human milk for volatile organic compounds. J Environ Monit 12:1265-1273.

Blum A, Gold MD, Ames BN, Jones FR, Hett EA, Dougherty RC, et al. 1978. Children absorb tris-BP flame retardant from sleepwear: urine contains the mutagenic metabolite, 2,3-dibromopropanol. Science 201:1020-1023.

Boogaard PJ. 2002. Use of haemoglobin adducts in exposure monitoring and risk assessment. J Chromatogr B Analyt Technol Biomed Life Sci 778:309-322.

Bowman MC, King JR, Holder CL. 1976. Benzidine and congeners: analytical chemical properties and trace analysis in five substances. Int J Environ Anal Chem 4:205-223.

Boysen G, Hecht SS. 2003. Analysis of DNA and protein adducts of benzo[a]pyrene in human tissues using structure-specific methods. Mutat Res 543:17-30.

Boysen G, Georgieva NI, Bordeerat NK, Sram RJ, Vacek P, Albertini RJ, et al. 2012. Formation of 1,2:3,4-diepoxybutane-specific hemoglobin adducts in 1,3-butadiene exposed workers. Toxicol Sci 125:30-40.

Bradman A, Castorina R, Barr DB, Chevrier J, Harnly ME, Eisen EA, et al. 2011. Determinants of organophosphorus pesticide urinary metabolite levels in young children living in an agricultural community. Int J Environ Res Public Health 8:1061-1083.

Brantsaeter AL, Haugen M, Mul Ad, Bjellaas T, Becher G, Klaveren JV, et al. 2008. Exploration of different methods to assess dietary acrylamide exposure in pregnant women participating in the Norwegian Mother and Child Cohort Study (MoBa). Food Chem Toxicol 46:2808-2814.

Brown WE, Burkert AL. 2002. Biomarkers of toluene diisocyanate exposure. Appl Occup Environ Hyg 17:840-845.

Brunborg G, Holme JA, Soderlund EJ, Hongslo JK, Vartiainen T, Lotjonen S, et al. 1991. Genotoxic effects of the drinking water mutagen 3-chloro-4-(dichloromethyl)-5-hydroxy-2[5H]-furanone (MX) in mammalian cells in vitro and in rats in vivo. Mutat Res 260:55-64.

Budworth H, Snijders AM, Marchetti F, Mannion B, Bhatnagar S, Kwoh E, et al. 2012. DNA repair and cell cycle biomarkers of radiation exposure and inflammation stress in human blood. PLoS ONE 7:e48619.

Busquets R, Jönsson JA, Frandsen H, Puignou L, Galceran MT, Skog K. 2009. Hollow fibre-supported liquid membrane extraction and LC-MS/MS detection for the analysis of heterocyclic amines in urine samples. Mol Nutr Food Res 53:1496-1504.

Cailleux A, Cogny M, Allain P. 1992. Blood isoprene concentrations in humans and in some animal species. Biochem Med Metab Biol 47:157-160.

Cailleux A, Moreau X, Delhumeau A, Allain P. 1993. Decrease of isoprene concentrations in blood during general anesthesia. Biochem Med Metab Biol 49:321-325.

Calafat AM, Barr DB, Pirkle JL, Ashley DL. 1999. Reference range concentrations of N-acetyl-S-(2-hydroxyethyl)-L-cysteine, a common metabolite of several volatile organic compounds, in the urine of adults in the United States. J Expo Anal Environ Epidemiol 9:336-342.

Calafat AM, Kuklenyik Z, Reidy JA, Caudill SP, Tully JS, Needham LL. 2007a. Serum concentrations of 11 polyfluoroalkyl compounds in the U.S. population: data from the national health and nutrition examination survey (NHANES). Environ Sci Technol 41:2237-2242.

Calafat AM, Wong LY, Kuklenyik Z, Reidy JA, Needham LL. 2007b. Polyfluoroalkyl chemicals in the U.S. population: data from the National Health and Nutrition Examination Survey (NHANES) 2003-2004 and comparisons with NHANES 1999-2000. Environ Health Perspect 115:1596-1602.

Caldow M, Sharman M, Kelly M, Day J, Hird S, Tarbin JA. 2009. Multi-residue determination of phenolic and salicylanilide anthelmintics and related compounds in bovine kidney by liquid chromatography-tandem mass spectrometry. J Chromatogr A 1216:8200-8205.

California OEHHA. 2014. Proposition 65 - list of chemicals. Available: http://www.oehha.ca.gov/prop65/prop65_list/Newlist.html [accessed 7 April 2014].

Cao LL, Yan CH, Yu XD, Tian Y, Zou XY, Lu DS, et al. 2012. Determination of polychlorinated biphenyls and organochlorine pesticides in human serum by gas chromatography with micro-electron capture detector. J Chromatogr Sci 50:145-150.

Cao Z, Swift TA, West CA, Rosano TG, Rej R. 2004. Immunoassay of estradiol: unanticipated suppression by unconjugated estriol. Clin Chem 50:160-165.

Carmella SG, Chen M, Han S, Briggs A, Jensen J, Hatsukami DK, et al. 2009. Effects of smoking cessation on eight urinary tobacco carcinogen and toxicant biomarkers. Chem Res Toxicol 22:734-741.

CDC. 2005. Third national report on human exposure to environmental chemicals. Available: http://cfpub.epa.gov/ncea/cfm/recordisplay.cfm?deid=2825 [accessed 8 April 2014].

CDC. 2006. Laboratory procedure manual: PCBs and persistent pesticides. Available: http://www.cdc.gov/nchs/data/nhanes/nhanes_03_04/l28_c_met_PCBs_and_Persistent_Pesticides.pdf [accessed 8 April 2014].

CDC. 2008a. 2003-2004 data documentation, code book, and frequencies: acrylamide and glycidamide (L06Age_C). Available: http://www.cdc.gov/nchs/nhanes/nhanes2003-2004/L06AGE_C.htm [accessed 7 April 2014].

CDC. 2008b. Laboratory procedure manual: volatile organic compounds (VOCs). Method No:13-OD; VO-BTHM-1.01. Available: http://www.cdc.gov/nchs/data/nhanes/nhanes_01_02/l04voc_b_met_in_blood.pdf [accessed 8 April 2014].

CDC. 2009. Fourth National Report on Human Exposure to Environmental Chemicals. Atlanta, GA:Centers For Disease Control and Prevention National Center for Health Statistics.

CDC. 2011. 2003 - 2004 data documentation, codebook, and frequencies:urinary current use pesticides (formerly priority pesticides, non-persistent pesticide metabolites) (L26UPP_C). Available: http://www.cdc.gov/nchs/nhanes/nhanes2003-2004/l26upp_c.htm [accessed 7 April 2014].

CDC. 2012a. Benzidine dyes action plan summary. Available: http://www.epa.gov/opptintr/existingchemicals/pubs/actionplans/benzidine.html [accessed 8 April 2014].

CDC. 2012b. National biomonitoring program factsheet: styrene. Available: http://www.cdc.gov/biomonitoring/Styrene_FactSheet.html [accessed 8 April 2014].

CDC. 2013. Biomonitoring summary: organochlorine pesticides overview. Available: http://www.cdc.gov/biomonitoring/ChlordaneHeptachlor_BiomonitoringSummary.html [accessed 8 April 2014].

Chen KM, El-Bayoumy K, Cunningham J, Aliaga C, Li H, Melikian AA. 2004. Detection of nitrated benzene metabolites in bone marrow of B6C3F1 mice treated with benzene. Chem Res Toxicol 17:370-377.

Cheng TJ, Huang YF, Ma YC. 2001. Urinary thiodiglycolic acid levels for vinyl chloride monomer-exposed polyvinyl chloride workers. J Occup Environ Med 43:934-938.

Chevrier C, Limon G, Monfort C, Rouget F, Garlantezec R, Petit C, et al. 2011. Urinary biomarkers of prenatal atrazine exposure and adverse birth outcomes in the PELAGIE birth cohort. Environ Health Perspect 119:1034-1041.

Choudhury S, Karara AH, Ace LN, McFarland VA. 1990. High-performance liquid chromatographic method for the determination of 3-methylcholanthrene in channel catfish plasma. J Chromatogr 534:208-213.

Cocker J, Cain JR, Baldwin P, McNally K, Jones K. 2009. A survey of occupational exposure to 4,4'-methylene-bis (2-chloroaniline) (MbOCA) in the UK. Ann Occup Hyg 53:499-507.

Cocker J. 2011. Biological monitoring for isocyanates. Ann Occup Hyg 55:127-131.

Cohen-Wolkowiez M, White NR, Bridges A, Benjamin DK, Jr., Kashuba AD. 2011. Development of a liquid chromatography-tandem mass spectrometry assay of six antimicrobials in plasma for pharmacokinetic studies in premature infants. J Chromatogr B Analyt Technol Biomed Life Sci 879:3497-3506.

Cooper EM, Covaci A, van Nuijs AL, Webster TF, Stapleton HM. 2011. Analysis of the flame retardant metabolites bis(1,3-dichloro-2-propyl) phosphate (BDCPP) and diphenyl phosphate (DPP) in urine using liquid chromatography-tandem mass spectrometry. Anal Bioanal Chem 401:2123-2132.

Coronado GD, Holte S, Vigoren E, Griffith WC, Barr DB, Faustman E, et al. 2011. Organophosphate pesticide exposure and residential proximity to nearby fields: evidence for the drift pathway. J Occup Environ Med 53:884-891.

Coronel MB, Sanchis V, Ramos AJ, Marin S. 2009. Assessment of the exposure to ochratoxin A in the province of Lleida, Spain. Food Chem Toxicol 47:2847-2852.

Cruz-Vera M, Lucena R, Cardenas S, Valcarcel M. 2009. One-step in-syringe ionic liquid-based dispersive liquid-liquid microextraction. J Chromatogr A 1216:6459-6465.

Csanády GA, Filser JG. 2001. Toxicokinetics of inhaled and endogenous isoprene in mice, rats, and humans. Chem Biol Interact 135-136:679-685.

Curwin BD, Hein MJ, Barr DB, Striley C. 2010. Comparison of immunoassay and HPLC-MS/MS used to measure urinary metabolites of atrazine, metolachlor, and chlorpyrifos from farmers and non-farmers in Iowa. J Expo Sci Environ Epidemiol 20:205-212.

Czene K, Osterman-Golkar S, Yun X, Li G, Zhao F, Pérez HL, et al. 2002. Analysis of DNA and hemoglobin adducts and sister chromatid exchanges in a human population occupationally exposed to propylene oxide: a pilot study. Cancer Epidemiol Biomarkers Prev 11:315-318.

Dalene M, Skarping G, Brorson T. 1990. Chromatographic determination of amines in biological fluids with special reference to the biological monitoring of isocyanates and amines. IV. Determination of 1,6-hexamethylenediamine in human urine using capillary gas chromatography and selective ion monitoring. J Chromatogr 516:405-413.

Dallinga JW, Pachen DM, Wijnhoven SW, Breedijk A, van 't Veer L, Wigbout G, et al. 1998. The use of 4-aminobiphenyl hemoglobin adducts and aromatic DNA adducts in lymphocytes of smokers as biomarkers of exposure. Cancer Epidemiol Biomarkers Prev 7:571-577.

Dangwal SK, Kadam VT. 1980. Determination of urinary nitrobenzene by the microdiffusion method. Am Ind Hyg Assoc J 41:146-148.

Davies NW, Veronese ME, McLean S. 1984. Mass spectrometric determination of N-hydroxyphenacetin in urine using multiple metastable peak monitoring following thin-layer chromatography. J Chromatogr 310:179-187.

De Alwis GKH, Needham LL, Barr DB. 2007. Automated solid phase extraction and quantitative measurement of 2,3-dibromo-1-propanol in urine using gas chromatography-mass spectrometry. Arch Environ Contam Toxicol 53:134-139.

de Cock J, Heederik D, Hoek F, Boleij J, Kromhout H. 1995. Urinary excretion of tetrahydrophtalimide in fruit growers with dermal exposure to captan. Am J Ind Med 28:245-256.

de Freitas Silva M, Schramm SG, Kano EK, Koono EE, Manfio JL, Porta V, et al. 2012. Metronidazole immediate release formulations: a fasting randomized open-label crossover bioequivalence study in healthy volunteers. Arzneimittelforschung 62:490-495.

de Jonge ME, van Dam SM, Hillebrand MJX, Rosing H, Huitema ADR, Rodenhuis S, et al. 2004. Simultaneous quantification of cyclophosphamide, 4-hydroxycyclophosphamide, N,N',N"-triethylenethiophosphoramide (thiotepa) and N,N',N"-triethylenephosphoramide (tepa) in human plasma by high-performance liquid chromatography coupled with electrospray ionization tandem mass spectrometry. J Mass Spectrom 39:262-271.

Del Santo P, Moneti G, Salvadori M, Saltutti C, Delle Rose A, Dolara P. 1991. Levels of the adducts of 4-aminobiphenyl to hemoglobin in control subjects and bladder carcinoma patients. Cancer Lett 60:245-251.

Delfino RJ, Gong H, Linn WS, Hu Y, Pellizzari ED. 2003. Respiratory symptoms and peak expiratory flow in children with asthma in relation to volatile organic compounds in exhaled breath and ambient air. J Expo Sci Environ Epidemiol 13:348-363.

Dickson LC, MacNeil JD, Reid J, Fesser AC. 2003. Validation of screening method for residues of diethylstilbestrol, dienestrol, hexestrol, and zeranol in bovine urine using immunoaffinity chromatography and gas chromatography/mass spectrometry. J AOAC Int 86:631-639.

Dittmann B, Renner G. 1977. 4-Acetaminophenoxyacetic acid, a new urinary metabolite of phenacetin. Naunyn Schmiedebergs Arch Pharmacol 296:87-89.

do Nascimento TG, Oliveira Ede J, Macedo RO. 2005. Simultaneous determination of ranitidine and metronidazole in human plasma using high performance liquid chromatography with diode array detection. J Pharm Biomed Anal 37:777-783.

Donald PR. 2010. Cerebrospinal fluid concentrations of antituberculosis agents in adults and children. Tuberculosis (Edinb) 90:279-292.

Du J, Hao L, Li Y, Lu J. 2007. Flow injection chemiluminescence determination of nitrofurazone in pharmaceutical preparations and biological fluids based on oxidation by singlet oxygen generated in N-bromosuccinimide-hydrogen peroxide reaction. Anal Chim Acta 582:98-102.

Duarte S, Bento J, Pena A, Lino CM, Delerue-Matos C, Oliva-Teles T, et al. 2010. Monitoring of ochratoxin A exposure of the Portuguese population through a nationwide urine survey--Winter 2007. Sci Total Environ 408:1195-1198.

Dybing E, Farmer PB, Andersen M, Fennell TR, Lalljie SPD, Müller DJG, et al. 2005. Human exposure and internal dose assessments of acrylamide in food. Food Chem Toxicol 43:365-410.

Dybing E, O'Brien J, Renwick AG, Sanner T. 2008. Risk assessment of dietary exposures to compounds that are genotoxic and carcinogenic--an overview. Toxicol Lett 180:110-117.

ECHA. 2011a. Annex XV – identification of 1,2,3-TCP as SVHC. Available: http://echa.europa.eu/documents/10162/435e1050-2ba6-4728-8efb-e378e7f93280 [accessed 8 April 2014].

ECHA. 2011b. Annex XV – identification of 1,2-dichloroethane as SVHC. Available: http://echa.europa.eu/documents/10162/57c002b0-db53-4f7c-ac5e-7c8dd46a53bf [accessed 8 April 2014].

ECHA. 2013. Candidate list of substances of very high concern for authorization. Available: http://echa.europa.eu/candidate-list-table [accessed 7 April 2014].

Eckert E, Drexler H, Goen T. 2010. Determination of six hydroxyalkyl mercapturic acids in human urine using hydrophilic interaction liquid chromatography with tandem mass spectrometry (HILIC-ESI-MS/MS). J Chromatogr B Analyt Technol Biomed Life Sci 878:2506-2514.

Eckert E, Schmid K, Schaller B, Hiddemann-Koca K, Drexler H, Goen T. 2011. Mercapturic acids as metabolites of alkylating substances in urine samples of German inhabitants. Int J Hyg Environ Health 214:196-204.

Eckert E, Leng G, Gries W, Goen T. 2012. A method for the simultaneous determination of mercapturic acids as biomarkers of exposure to 2-chloroprene and epichlorohydrin in human urine. J Chromatogr B Analyt Technol Biomed Life Sci 889-890:69-76.

Eckert E, Leng G, Gries W, Goen T. 2013. Excretion of mercapturic acids in human urine after occupational exposure to 2-chloroprene. Arch Toxicol.

Egeghy PP, Cohen Hubal EA, Tulve NS, Melnyk LJ, Morgan MK, Fortmann RC, et al. 2011. Review of pesticide urinary biomarker measurements from selected US EPA children's observational exposure studies. Int J Environ Res Public Health 8:1727-1754.

Environment Canada. 2011. Proposed risk management approach for hydrazine. Available: http://www.ec.gc.ca/ese-ees/default.asp?lang=En&n=BF03ABB4-1 [accessed 8 April 2014].

Erkekoğlu P, Sabuncuoğlu S, Aydin S, Sahin G, Giray B. 2010. Determination of seasonal variations in serum ochratoxin A levels in healthy population living in some regions of Turkey by enzyme-linked immunosorbent assay. Toxicon 55:507-513.

Espinosa-Mansilla A, Acedo-Valenzuela MI, Munoz de la Pena A, Canada Canada F, Salinas Lopez F. 2002. Determination of antitubercular drugs in urine and pharmaceuticals by LC using a gradient flow combined with programmed diode array photometric detection. Talanta 58:273-280.

European Commission. 2009. Commission regulation no. 552/2009 (REACH Annex XVII). Available: http://eur-lex.europa.eu/LexUriServ/LexUriServ.do?uri=OJ:L:2009:164:0007:0031:EN:PDF [accessed 8 April 2014].

Everett CJ, Matheson EM. 2010. Biomarkers of pesticide exposure and diabetes in the 1999-2004 national health and nutrition examination survey. Environ Int 36:398-401.

Favier B, Gilles L, Desage M, Latour J-F. 2003. [Analysis of cyclophosphamide in the urine of antineoplastic drugs handlers]. Bull Cancer (Paris) 90:905-909.

Fennell TR, MacNeela JP, Morris RW, Watson M, Thompson CL, Bell DA. 2000. Hemoglobin adducts from acrylonitrile and ethylene oxide in cigarette smokers: effects of glutathione S-transferase T1-null and M1-null genotypes. Cancer Epidemiol Biomarkers Prev 9:705-712.

Fernandez MF, Santa-Marina L, Ibarluzea JM, Exposito J, Aurrekoetxea JJ, Torne P, et al. 2007. Analysis of population characteristics related to the total effective xenoestrogen burden: a biomarker of xenoestrogen exposure in breast cancer. Eur J Cancer 43:1290-1299.

Filser JG, Hutzler C, Rampf F, Kessler W, Faller TH, Leibold E, et al. 2008. Concentrations of the propylene metabolite propylene oxide in blood of propylene-exposed rats and humans--a basis for risk assessment. Toxicol Sci 102:219-231.

Frandsen H, Frederiksen H, Alexander J. 2002. 2-Amino-1-methyl-6-(5-hydroxy-)phenylimidazo[4,5-b]pyridine (5-OH-PhIP), a biomarker for the genotoxic dose of the heterocyclic amine, 2-amino-1-methyl-6-phenylimidazo[4,5-b]pyridine (PhIP). Food Chem Toxicol 40:1125-1130.

Fransman W, Peelen S, Hilhorst S, Roeleveld N, Heederik D, Kromhout H. 2007. A pooled analysis to study trends in exposure to antineoplastic drugs among nurses. Ann Occup Hyg 51:231-239.

Friedman GD, Jiang SF, Udaltsova N, Chan J, Quesenberry CP, Jr., Habel LA. 2009. Pharmaceuticals that cause mammary gland tumors in animals: findings in women. Breast Cancer Res Treat 116:187-194.

Fromme H, Mosch C, Morovitz M, Alba-Alejandre I, Boehmer S, Kiranoglu M, et al. 2010. Pre- and postnatal exposure to perfluorinated compounds (PFCs). Environ Sci Technol 44:7123-7129.

Fujioka Y, Metsugi Y, Ogawara K, Higaki K, Kimura T. 2008. Evaluation of in vivo dissolution behavior and GI transit of griseofulvin, a BCS class II drug. Int J Pharm 352:36-43.

Funk WE, Waidyanatha S, Chaing SH, Rappaport SM. 2008. Hemoglobin adducts of benzene oxide in neonatal and adult dried blood spots. Cancer Epidemiol Biomarkers Prev 17:1896-1901.

Fustinoni S, Campo L, Manini P, Buratti M, Waidyanatha S, De Palma G, et al. 2008. An integrated approach to biomonitoring exposure to styrene and styrene-(7,8)-oxide using a repeated measurements sampling design. Biomarkers 13:560-578.

Fustinoni S, Manini P, Campo L, De Palma G, Andreoli R, Mutti A, et al. 2010. Assessing variability and comparing short-term biomarkers of styrene exposure using a repeated measurements approach. Toxicol Lett 192:40-44.

Gaber K, Harreus UA, Matthias C, Kleinsasser NH, Richter E. 2007. Hemoglobin adducts of the human bladder carcinogen o-toluidine after treatment with the local anesthetic prilocaine. Toxicology 229:157-164.

Gagne S. 2012. A reliable method by ultra-performance liquid chromatography coupled with tandem mass spectrometry to quantify and confirm simultaneously the presence of solvent metabolites in workers' urine. Rapid Commun Mass Spectrom 26:845-852.

Gammon MD, Santella RM, Neugut AI, Eng SM, Teitelbaum SL, Paykin A, et al. 2002. Environmental toxins and breast cancer on Long Island. I. Polycyclic aromatic hydrocarbon DNA adducts. Cancer Epidemiol Biomarkers Prev 11:677-685.

Gardner I, Bergin P, Stening P, Kenna JG, Caldwell J. 1996. Immunochemical detection of covalently modified protein adducts in livers of rats treated with methyleugenol. Chem Res Toxicol 9:713-721.

Garland WA, Hsiao KC, Pantuck EJ, Conney AH. 1977. Quantitative determination of phenacetin and its metabolite acetaminophen by GLC-chemical ionization mass spectrometry. J Pharm Sci 66:340-344.

Ge Z, Wang Z, Wei M. 2008. Measurement of the concentration of three antituberculosis drugs in the focus of spinal tuberculosis. Eur Spine J 17:1482-1487.

Gerbl U, Cichna M, Zsivkovits M, Knasmüller S, Sontag G. 2004. Determination of heterocyclic aromatic amines in beef extract, cooked meat and rat urine by liquid chromatography with coulometric electrode array detection. J Chromatogr B Analyt Technol Biomed Life Sci 802:107-113.

Gherardi M, Gordiani A, Gatto M. 2010. Development and validation of method for analysis of some ototoxic solvents in saliva matrix by headspace gas chromatography/mass spectrometry. J Chromatogr B Analyt Technol Biomed Life Sci 878:2391-2396.

Gladen BC, Zadorozhnaja TD, Chislovska N, Hryhorczuk DO, Kennicutt MC, Little RE. 2000. Polycyclic aromatic hydrocarbons in placenta. Hum Exp Toxicol 19:597-603.

Gobba F, Ghittori S, Imbriani M, Maestri L, Capodaglio E, Cavalleri A. 1997. The urinary excretion of solvents and gases for the biological monitoring of occupational exposure: a review. Sci Total Environ 199:3-12.

Gonzalez-Reche LM, Koch HM, Weiss T, Müller J, Drexler H, Angerer J. 2002. Analysis of ethenoguanine adducts in human urine using high performance liquid chromatography-tandem mass spectrometry. Toxicol Lett 134:71-77.

Gordon SM, Wallace LA, Brinkman MC, Callahan PJ, Kenny DV. 2002. Volatile organic compounds as breath biomarkers for active and passive smoking. Environ Health Perspect 110:689-698.

Gotelli GR, Kabra PM, Marton LJ. 1977. Determination of acetaminophen and phenacetin in plasma by high-pressure liquid chromatography. Clin Chem 23:957-959.

Gramatte T. 1994. Griseofulvin absorption from different sites in the human small intestine. Biopharm Drug Dispos 15:747-759.

Grimmer G, Dettbarn G, Seidel A, Jacob J. 2000. Detection of carcinogenic aromatic amines in the urine of non-smokers. Sci Total Environ 247:81-90.

Gu D, McNaughton L, Lemaster D, Lake BG, Gooderham NJ, Kadlubar FF, et al. 2010. A comprehensive approach to the profiling of the cooked meat carcinogens 2-amino-3,8-dimethylimidazo[4,5-f]quinoxaline, 2-amino-1-methyl-6-phenylimidazo[4,5-b]pyridine, and their metabolites in human urine. Chem Res Toxicol.

Gu D, Turesky RJ, Tao Y, Langouet SA, Nauwelaers GC, Yuan JM, et al. 2012. DNA adducts of 2-amino-1-methyl-6-phenylimidazo[4,5-b]pyridine and 4-aminobiphenyl are infrequently detected in human mammary tissue by liquid chromatography/tandem mass spectrometry. Carcinogenesis 33:124-130.

Guerbet M, Brisorgueuil E, Jolibois B, Caillard J-F, Gehanno J-F. 2007. Evaluation of urinary mutagenicity in azo dye manufacture workers. Int J Occup Med Environ Health 20:137-145.

Gürbay A, Girgin G, Sabuncuoglu SA, Sahin G, Yurdakök M, Yigit S, et al. 2009. Ochratoxin A: is it present in breast milk samples obtained from mothers from Ankara, Turkey? J Appl Toxicol.

Hagmar L, Wirfält E, Paulsson B, Törnqvist M. 2005. Differences in hemoglobin adduct levels of acrylamide in the general population with respect to dietary intake, smoking habits and gender. Mutat Res 580:157-165.

Hami M, Taibi F, Smagghe G, Soltani-Mazouni N. 2005. Comparative toxicity of three ecdysone agonist insecticides against the Mediterranean flour moth. Commun Agric Appl Biol Sci 70:767-773.

Hammond SK, Coghlin J, Gann PH, Paul M, Taghizadeh K, Skipper PL, et al. 1993. Relationship between environmental tobacco smoke exposure and carcinogen-hemoglobin adduct levels in nonsmokers. J Natl Cancer Inst 85:474-478.

Haneke K. 2002. Tetrabromobisphenol A bis(2,3-dibromopropyl ether) [21850-44-2]: review of toxicological literature. Available: http://ntp.niehs.nih.gov/ntp/htdocs/Chem_Background/ExSumPdf/TBBPA-BDPE_508.pdf [accessed 8 April 2014].

Harada KH, Koizumi A. 2009. Environmental and biological monitoring of persistent fluorinated compounds in Japan and their toxicities. Environ Health Prev Med 14:7-19.

Haraguchi K, Koizumi A, Inoue K, Harada KH, Hitomi T, Minata M, et al. 2009. Levels and regional trends of persistent organochlorines and polybrominated diphenyl ethers in Asian breast milk demonstrate POPs signatures unique to individual countries. Environ Int 35:1072-1079.

Hartmann EC, Boettcher MI, Schettgen T, Fromme H, Drexler H, Angerer J. 2008. Hemoglobin adducts and mercapturic acid excretion of acrylamide and glycidamide in one study population. J Agric Food Chem 56:6061-6068.

Hatfield TR, Roberts EC, Bell IF, Clunie JC, Kalla PJ, McKay DL. 1982. Urine monitoring of textile workers exposed to dichlorobenzidine-derived pigments. J Occup Med 24:656-658.

Health Canada. 1993. Styrene - PSL 1 (archived). Available: http://www.hc-sc.gc.ca/ewh-semt/pubs/contaminants/psl1-lsp1/styrene/index-eng.php [accessed 8 April 2014].

Health Canada. 1994. 1,2-Dichloroethane - PSL1 (archived). Available: http://www.hc-sc.gc.ca/ewh-semt/pubs/contaminants/psl1-lsp1/1_2_dichloroethane/index-eng.php - a23 [accessed 8 April 2014].

Health Canada. 2000. Priority substances list assessment report - 1,3-butadiene (archived). Available: http://www.hc-sc.gc.ca/ewh-semt/pubs/contaminants/psl2-lsp2/1_3_butadiene/index-eng.php - a2.3 [accessed 8 April 2014].

Health Canada. 2007a. Benzene - PSL 1 (archived). Available: http://www.hc-sc.gc.ca/ewh-semt/pubs/contaminants/psl1-lsp1/benzene/index-eng.php [accessed 8 April 2014].

Health Canada. 2007b. 3,3'-dichlorobenzidine - PSL 1 (archived). Available: http://www.hc-sc.gc.ca/ewh-semt/pubs/contaminants/psl1-lsp1/3-dichlorobenzidine/index-eng.php [accessed 8 April 2014].

Health Canada. 2007c. Benzidine - PSL 1 (archived). Available: http://www.hc-sc.gc.ca/ewh-semt/pubs/contaminants/psl1-lsp1/benzidine/index-eng.php [accessed 8 April 2014].

Hecht SS, Seow A, Wang M, Wang R, Meng L, Koh WP, et al. 2010. Elevated levels of volatile organic carcinogen and toxicant biomarkers in Chinese women who regularly cook at home. Cancer Epidemiol Biomarkers Prev 19:1185-1192.

Hedley AJ, Hui LL, Kypke K, Malisch R, van Leeuwen FX, Moy G, et al. 2010. Residues of persistent organic pollutants (POPs) in human milk in Hong Kong. Chemosphere 79:259-265.

Hedmer M, Höglund P, Cavallin-Ståhl E, Albin M, Jönsson BAG. 2008. Validation of urinary excretion of cyclophosphamide as a biomarker of exposure by studying its renal clearance at high and low plasma concentrations in cancer patients. Int Arch Occup Environ Health 81:285-293.

Heinig R, Zimmer D, Yeh S, Krol GJ. 2000. Development, validation and application of assays to quantify metrifonate and 2,2-dichlorovinyl dimethylphosphate in human body fluids. J Chromatogr B Biomed Sci Appl 741:257-269.

Hester TR, Ford NF, Gale PJ, Hammett JL, Raymond R, Turnbull D, et al. 1997. Measurement of 2,4-toluenediamine in urine and serum samples from women with Même or Replicon breast implants. Plast Reconstr Surg 100:1291-1298.

Heudorf U, Hartmann E, Angerer J. 2009. Acrylamide in children--exposure assessment via urinary acrylamide metabolites as biomarkers. Int J Hyg Environ Health 212:135-141.

Hines CJ, Deddens JA, Jaycox LB, Andrews RN, Striley CA, Alavanja MC. 2008. Captan exposure and evaluation of a pesticide exposure algorithm among orchard pesticide applicators in the Agricultural Health Study. Ann Occup Hyg 52:153-166.

Hirvonen A. 2005. Gene-environment interaction and biological monitoring of occupational exposures. Toxicol Appl Pharmacol 207:329-335.

Hoehle SI, Knudsen GA, Sanders JM, Sipes IG. 2009. Absorption, distribution, metabolism, and excretion of 2,2-bis(bromomethyl)-1,3-propanediol in male fischer-344 rats. Drug Metab Dispos 37:408-416.

Hoffer E, Tabak A, Shcherb I, Wiener A, Bentur Y. 2005. Monitoring of occupational exposure to methylene chloride: sampling protocol and stability of urine samples. J Anal Toxicol 29:794-798.

Honour JW. 2006. High-performance liquid chromatography for hormone assay. Methods Mol Biol 324:25-52.

Honour JW. 2010. Steroid assays in paediatric endocrinology. J Clin Res Pediatr Endocrinol 2:1-16.

Hooper DG, Bolton VE, Guilford FT, Straus DC. 2009. Mycotoxin detection in human samples from patients exposed to environmental molds. Int J Mol Sci 10:1465-1475.

Hoover RN, Hyer M, Pfeiffer RM, Adam E, Bond B, Cheville AL, et al. 2011. Adverse health outcomes in women exposed in utero to diethylstilbestrol. N Engl J Med 365:1304-1314.

Houde M, Martin JW, Letcher RJ, Solomon KR, Muir DC. 2006. Biological monitoring of polyfluoroalkyl substances: A review. Environ Sci Technol 40:3463-3473.

Hsu FF, Lakshmi V, Rothman N, Bhatnager VK, Hayes RB, Kashyap R, et al. 1996. Determination of benzidine, N-acetylbenzidine, and N,N'-diacetylbenzidine in human urine by capillary gas chromatography/negative ion chemical ionization mass spectrometry. Anal Biochem 234:183-189.

Hsu FF, Lakshmi VM, Zenser TV. 2009. Characterization of new metabolites from in vivo biotransformation of 2-amino-3-methylimidazo[4,5-f]quinoline in mouse by mass spectrometry. J Mass Spectrom 44:1359-1368.

Huang CC, Li CM, Wu CF, Jao SP, Wu KY. 2007. Analysis of urinary N-acetyl-S-(propionamide)-cysteine as a biomarker for the assessment of acrylamide exposure in smokers. Environ Res 104:346-351.

Huang CC, Shih WC, Wu CF, Chen MF, Chen YL, Lin YH, et al. 2008. Rapid and sensitive on-line liquid chromatographic/tandem mass spectrometric determination of an ethylene oxide-DNA adduct, N7-(2-hydroxyethyl)guanine, in urine of nonsmokers. Rapid Commun Mass Spectrom 22:706-710.

Huang L, Marzan F, Jayewardene AL, Lizak PS, Li X, Aweeka FT. 2009. Development and validation of a hydrophilic interaction liquid chromatography-tandem mass spectrometry method for determination of isoniazid in human plasma. J Chromatogr B Analyt Technol Biomed Life Sci 877:285-290.

Huang YF, Chen ML, Liou SH, Chen MF, Uang SN, Wu KY. 2011a. Association of CYP2E1, GST and mEH genetic polymorphisms with urinary acrylamide metabolites in workers exposed to acrylamide. Toxicol Lett 203:118-126.

Huang YF, Wu KY, Liou SH, Uang SN, Chen CC, Shih WC, et al. 2011b. Biological monitoring for occupational acrylamide exposure from acrylamide production workers. Int Arch Occup Environ Health 84:303-313.

Hurst HE, Ali MY. 2007. Analyses of (1-chloroethenyl)oxirane headspace and hemoglobin N-valine adducts in erythrocytes indicate selective detoxification of (1-chloroethenyl)oxirane enantiomers. Chem Biol Interact 166:332-340.

Huyck S, Ohman-Strickland P, Zhang L, Tong J, Xu XU, Zhang JJ. 2010. Determining times to maximum urine excretion of 1-aminopyrene after diesel exhaust exposure. J Expo Sci Environ Epidemiol 20:650-655.

IARC. 1979. Vinyl chloride. IARC Monogr Eval Carcinog Risks Hum 19:377.

IARC. 1981. Cyclophosphamide. IARC Monogr Eval Carcinog Risks Hum 26:165.

IARC. 1982. Benzene. IARC Monogr Eval Carcinog Risks Hum 29:93.

IARC. 1983a. Nithiazide. IARC Monogr Eval Carcinog Risks Hum 31:179.

IARC. 1983b. 3-amino-1-methyl-5h-pyrido[4,3-b]indole. IARC Monogr Eval Carcinog Risks Hum 31:255.

IARC. 1983c. Dibenzo[def,p]chrysene. IARC Monogr Eval Carcinog Risks Hum 32:343.

IARC. 1983d. Dibenz[a,h]anthracene. IARC Monogr Eval Carcinog Risks Hum 32:299.

IARC. 1983e. AF-2 (2-(2-furyl)-3-(5-nitro-2-furyl) acrylamide). IARC Monogr Eval Carcinog Risks Hum 30:47.

IARC. 1986a. 1,2-dichloropropane. IARC Monogr Eval Carcinog Risks Hum 41:131.

IARC. 1986b. Bracken fern. IARC Monogr Eval Carcinog Risks Hum 40:47.

IARC. 1989a. 4-nitropyrene. IARC Monogr Eval Carcinog Risks Hum 46:367-373.

IARC. 1989b. 6-nitrochrysene. IARC Monogr Eval Carcinog Risks Hum 46:267-276.

IARC. 1989c. 1,3-dinitropyrene. IARC Monogr Eval Carcinog Risks Hum 46:201-213.

IARC. 1989d. 2-nitrofluorene. IARC Monogr Eval Carcinog Risks Hum 46:277-289.

IARC. 1989e. 1,8-dinitropyrene. IARC Monogr Eval Carcinog Risks Hum 46:231-246.

IARC. 1990. Nitrofurantoin. IARC Monogr Eval Carcinog Risks Hum 50:211-231.

IARC. 1991a. Simazine. IARC Monogr Eval Carcinog Risks Hum 53:495-513.

IARC. 1991b. Captafol. IARC Monogr Eval Carcinog Risks Hum 53:353-369.

IARC. 1991c. Dichlorvos. IARC Monogr Eval Carcinog Risks Hum 53:267-307.

IARC. 1993a. IQ (2-amino-3-methylimidazo[4,5-f]quinoline). IARC Monogr Eval Carcinog Risks Hum 56:165-195.

IARC. 1993b. Ochratoxin A. IARC Monogr Eval Carcinog Risks Hum 56:489.

IARC. 1993c. 4,4'-Methylenebis(2-chloroaniline) (MOCA). IARC Monogr Eval Carcinog Risks Hum 57:271-303.

IARC. 1993d. Magenta and CI Basic Red 9. IARC Monogr Eval Carcinog Risks Hum 57:215-234.

IARC. 1994. Propylene oxide (PO). IARC Monogr Eval Carcinog Risks Hum 60.

IARC. 1996a. Nitrobenzene. IARC Monogr Eval Carcinog Risks Hum 65:381-408.

IARC. 1996b. 2-Nitrotoluene, 3-nitrotoluene and 4-nitrotoluene. IARC Monogr Eval Carcinog Risks Hum 65:409-435.

IARC. 1999a. Carbon tetrachloride. IARC Monogr Eval Carcinog Risks Hum 71 Pt 2:401-432.

IARC. 1999b. Acrylonitrile. IARC Monogr Eval Carcinog Risks Hum 71 Pt 1:43-108.

IARC. 1999c. 1,4-Dioxane. IARC Monogr Eval Carcinog Risks Hum 71 Pt 2:589-602.

IARC. 1999d. 1,2-Dibromo-3-chloropropane. IARC Monogr Eval Carcinog Risks Hum 71 Pt 2:479-500.

IARC. 1999e. Chloroprene. IARC Monogr Eval Carcinog Risks Hum 71 Pt 1:227-250.

IARC. 1999f. Ethylene dibromide (1,2-dibromoethane). IARC Monogr Eval Carcinog Risks Hum 71 Pt 2:641-669.

IARC. 1999g. Atrazine. IARC Monogr Eval Carcinog Risks Hum 73:59-113.

IARC. 1999h. Simazine. IARC Monogr Eval Carcinog Risks Hum 73:625-640.

IARC. 1999i. 1,2-Dichloroethane. IARC Monogr Eval Carcinog Risks Hum 71 Pt 2:501-529.

IARC. 1999j. Dichloromethane. IARC Monogr Eval Carcinog Risks Hum 71 Pt 1:251-315.

IARC. 1999k. Hydrazine. IARC Monogr Eval Carcinog Risks Hum 71 Pt 3:991-1013.

IARC. 1999l. Vinylidene chloride. IARC Monogr Eval Carcinog Risks Hum 71 Pt 3:1163-1180.

IARC. 2000a. ortho-toluidine. IARC Monogr Eval Carcinog Risks Hum 77:267-322.

IARC. 2000b. Nitromethane. IARC Monogr Eval Carcinog Risks Hum 77:487-501.

IARC. 2000c. 2,2-Bis(bromomethyl)propane-1,3-diol. IARC Monogr Eval Carcinog Risks Hum 77:455-468.

IARC. 2001a. 2,4-diaminoanisole sulfate and its salts. IARC Monogr Eval Carcinog Risks Hum 79:621-648.

IARC. 2001b. Chlordane and heptachlor. IARC Monogr Eval Carcinog Risks Hum 79:411-492.

IARC. 2001c. Some thyrotropic agents. IARC Monogr Eval Carcinog Risks Hum 79:i-iv, 1-725.

IARC. 2002. Styrene. IARC Monogr Eval Carcinog Risks Hum 82:437-550.

IARC. 2004. 3-chloro-4-(dichloromethyl)-5-hydroxy-2(5h)-furanone (MX). IARC Monogr Eval Carcinog Risks Hum 84:441-475.

IARC. 2010. Some aromatic amines, organic dyes, and related exposures. IARC Monogr Eval Carcinog Risks Hum 99:1-658.

IARC. 2012. Diethylstilbestrol. IARC Monogr Eval Carcinog Risks Hum 100A:175-218.

Iavicoli I, Brera C, Carelli G, Caputi R, Marinaccio A, Miraglia M. 2002. External and internal dose in subjects occupationally exposed to ochratoxin A. Int Arch Occup Environ Health 75:381-386.

Il'yasova D, McCarthy BJ, Erdal S, Shimek J, Goldstein J, Doerge DR, et al. 2009. Human exposure to selected animal neurocarcinogens: a biomarker-based assessment and implications for brain tumor epidemiology. J Toxicol Environ Health B Crit Rev 12:175-187.

Inoue S, Saito T, Mase H, Suzuki Y, Takazawa K, Yamamoto I, et al. 2007. Rapid simultaneous determination for organophosphorus pesticides in human serum by LC-MS. J Pharm Biomed Anal 44:258-264.

Jafari MT, Rezaei B, Zaker B. 2009. Ion mobility spectrometry as a detector for molecular imprinted polymer separation and metronidazole determination in pharmaceutical and human serum samples. Anal Chem 81:3585-3591.

Johnson ES, Langard S, Lin YS. 2007. A critique of benzene exposure in the general population. Sci Total Environ 374:183-198.

Jones AL, Van der Woord M, Bourrillon F. 2005. Use of a whole blood competitive immunoassay for the assessment of worker exposures to propylene oxide at three manufacturing facilities. Ann Occup Hyg 49:241-243.

Jones CR, Liu Y-Y, Sepai O, Yan H, Sabbioni G. 2005. Hemoglobin adducts in workers exposed to nitrotoluenes. Carcinogenesis 26:133-143.

Jones CR, Sepai O, Liu YY, Yan H, Sabbioni G. 2005. Urinary metabolites of workers exposed to nitrotoluenes. Biomarkers 10:10-28.

Jones DE, Magnin-Bissel G, Holladay SD. 2009. Detection of polycyclic aromatic hydrocarbons in the shed skins of corn snakes (Elaphe guttata). Ecotoxicol Environ Saf 72:2033-2035.

Käfferlein HU, Marczynski B, Mensing T, Brüning T. 2010. Albumin and hemoglobin adducts of benzo[a]pyrene in humans--analytical methods, exposure assessment, and recommendations for future directions. Crit Rev Toxicol 40:126-150.

Kaklamanos G, Theodoridis G, Dabalis T. 2009. Determination of anabolic steroids in bovine urine by liquid chromatography-tandem mass spectrometry. J Chromatogr B Analyt Technol Biomed Life Sci 877:2330-2336.

Karima H-K, Ridha G, Zied A, Chekib M, Salem M, Abderrazek H. 2009. Estimation of Ochratoxin A in human blood of healthy Tunisian population. Exp Toxicol Pathol.

Keen C, Coldwell M, McNally K, Baldwin P, McAlinden J, Cocker J. 2011. A follow up study of occupational exposure to 4,4'-methylene-bis(2-chloroaniline) (MbOCA) and isocyanates in polyurethane manufacture in the UK. Toxicol Lett.

Kirchherr H. 1993. Determination of hydrazine in human plasma by high-performance liquid chromatography. J Chromatogr 617:157-162.

Knudsen LE, Hansen AM. 2007. Biomarkers of intermediate endpoints in environmental and occupational health. Int J Hyg Environ Health 210:461-470.

Kobayashi M, Hanaoka T, Tsugane S. 2007. Validity of a self-administered food frequency questionnaire in the assessment of heterocyclic amine intake using 2-amino-1-methyl-6-phenylimidazo[4,5-b]pyridine (PhIP) levels in hair. Mutat Res 630:14-19.

Kolpin DW, Furlong ET, Meyer MT, Thurman EM, Zaugg SD, Barber LB, et al. 2002. Pharmaceuticals, hormones, and other organic wastewater contaminants in U.S. streams, 1999-2000: A national reconnaissance. Environ Sci Technol 36:1202-1211.

Kopp EK, Sieber M, Kellert M, Dekant W. 2008. Rapid and sensitive HILIC-ESI-MS/MS quantitation of polar metabolites of acrylamide in human urine using column switching with an online trap column. J Agric Food Chem 56:9828-9834.

Kotapati S, Matter BA, Grant AL, Tretyakova NY. 2011. Quantitative analysis of trihydroxybutyl mercapturic acid, a urinary metabolite of 1,3-butadiene, in humans. Chem Res Toxicol 24:1516-1526.

Kovács F, Sándor G, Ványi A, Domány S, Zomborszky-Kovács M. 1995. Detection of ochratoxin a in human blood and colostrum. Acta Vet Hung 43:393-400.

Kuklenyik Z, Needham LL, Calafat AM. 2005. Measurement of 18 perfluorinated organic acids and amides in human serum using on-line solid-phase extraction. Anal Chem 77:6085-6091.

Kulp KS, Knize MG, Fowler ND, Salmon CP, Felton JS. 2004. PhIP metabolites in human urine after consumption of well-cooked chicken. J Chromatogr B Analyt Technol Biomed Life Sci 802:143-153.

Kunisue T, Muraoka M, Ohtake M, Sudaryanto A, Minh NH, Ueno D, et al. 2006. Contamination status of persistent organochlorines in human breast milk from Japan: recent levels and temporal trend. Chemosphere 64:1601-1608.

Kushch I, Arendacká B, Stolc S, Mochalski P, Filipiak W, Schwarz K, et al. 2008. Breath isoprene--aspects of normal physiology related to age, gender and cholesterol profile as determined in a proton transfer reaction mass spectrometry study. Clin Chem Lab Med 46:1011-1018.

Kushnir MM, Rockwood AL, Bergquist J. 2010. Liquid chromatography-tandem mass spectrometry applications in endocrinology. Mass Spectrom Rev 29:480-502.

Kütting B, Uter W, Drexler H. 2008. The association between self-reported acrylamide intake and hemoglobin adducts as biomarkers of exposure. Cancer Causes Control 19:273-281.

Kütting B, Göen T, Schwegler U, Fromme H, Uter W, Angerer J, et al. 2009. Monoarylamines in the general population--a cross-sectional population-based study including 1004 Bavarian subjects. Int J Hyg Environ Health 212:298-309.

Kutz FW, Wood PH, Bottimore DP. 1991. Organochlorine pesticides and polychlorinated biphenyls in human adipose tissue. Rev Environ Contam Toxicol 120:1-82.

Labat L, Thomas J, Dehon B, Humbert L, Leleu B, Nisse C, et al. 2006. [Assessment of occupational exposure to ortho-toluidine using gas chromatography-mass spectrometry]. Acta Clin Belg Suppl:63-67.

Lakshmi VM, Hsu FF, Zenser TV. 2009. Identification of new 2-amino-3-methylimidazo[4,5-f]quinoline urinary metabolites from beta-naphthoflavone-treated mice. Drug Metab Dispos 37:1690-1697.

Lau C, Anitole K, Hodes C, Lai D, Pfahles-Hutchens A, Seed J. 2007. Perfluoroalkyl acids: a review of monitoring and toxicological findings. Toxicol Sci 99:366-394.

Laumbach R, Tong J, Zhang L, Ohman-Strickland P, Stern A, Fiedler N, et al. 2009. Quantification of 1-aminopyrene in human urine after a controlled exposure to diesel exhaust. J Environ Monit 11:153-159.

Lee CH, Kamijima M, Kim H, Shibata E, Ueyama J, Suzuki T, et al. 2007. 8-Hydroxydeoxyguanosine levels in human leukocyte and urine according to exposure to organophosphorus pesticides and paraoxonase 1 genotype. Int Arch Occup Environ Health 80:217-227.

Lee DH, Lee IK, Song K, Steffes M, Toscano W, Baker BA, et al. 2006. A strong dose-response relation between serum concentrations of persistent organic pollutants and diabetes: results from the National Health and Examination Survey 1999-2002. Diabetes Care 29:1638-1644.

Lee JH, Shin H-S. 2002. Determination of hemoglobin adducts formed in rats exposed orally with 3,3'-dichlorobenzidine by GC/MS-SIM. Toxicol Ind Health 18:191-199.

Lezamiz J, Barri T, Jönsson JA, Skog K. 2008. A simplified hollow-fibre supported liquid membrane extraction method for quantification of 2-amino-1-methyl-6-phenylimidazo[4,5-b]pyridine (PhIP) in urine and plasma samples. Anal Bioanal Chem 390:689-696.

Li B, Ricordel I, Schopfer LM, Baud F, Mégarbane B, Masson P, et al. 2010. Dichlorvos, chlorpyrifos oxon and Aldicarb adducts of butyrylcholinesterase, detected by mass spectrometry in human plasma following deliberate overdose. J Appl Toxicol 30:559-565.

Li B, Ricordel I, Schopfer LM, Baud F, Mégarbane B, Nachon F, et al. 2010. Detection of adduct on tyrosine 411 of albumin in humans poisoned by dichlorvos. Toxicol Sci 116:23-31.

Li C-M, Hu C-W, Wu K-Y. 2005. Quantification of urinary N-acetyl-S- (propionamide)cysteine using an on-line clean-up system coupled with liquid chromatography/tandem mass spectrometry. J Mass Spectrom 40:511-515.

Li CM, Hu CW, Wu KY. 2005. Quantification of urinary N-acetyl-S- (propionamide)cysteine using an on-line clean-up system coupled with liquid chromatography/tandem mass spectrometry. J Mass Spectrom 40:511-515.

Li F, Miao Y, Zhang L, Neuenswander SA, Douglas JT, Ma X. 2011. Metabolomic analysis reveals novel isoniazid metabolites and hydrazones in human urine. Drug Metab Pharmacokinet 26:569-576.

Li H, He J, Liu Q, Huo Z, Liang S, Liang Y, et al. 2011. Simultaneous determination of hydrochlorothiazide and reserpine in human urine by LC with a simple pretreatment. Chromatographia 73:171-175.

Lin SJ, Chen YR, Su YH, Tseng HI, Chen SH. 2006. Determination of indomethacin in plasma by micellar electrokinetic chromatography with UV detection for premature infants with patent ducts arteriosus. J Chromatogr B Analyt Technol Biomed Life Sci 830:306-313.

Lino CM, Baeta ML, Henri M, Dinis AMP, Pena AS, Silveira MIN. 2008. Levels of ochratoxin A in serum from urban and rural Portuguese populations and estimation of exposure degree. Food Chem Toxicol 46:879-885.

Liu W, Zhang J, Li C, Tang L, Zhang Z, Yang M. 2013. A novel composite film derived from cysteic acid and PDDA-functionalized graphene: enhanced sensing material for electrochemical determination of metronidazole. Talanta 104:204-211.

Llorca M, Farre M, Pico Y, Teijon ML, Alvarez JG, Barcelo D. 2010. Infant exposure of perfluorinated compounds: levels in breast milk and commercial baby food. Environ Int 36:584-592.

Lodovici M, Akpan V, Giovannini L, Migliani F, Dolara P. 1998. Benzo[a]pyrene diol-epoxide DNA adducts and levels of polycyclic aromatic hydrocarbons in autoptic samples from human lungs. Chem Biol Interact 116:199-212.

Long C, Mai Z, Zhu B, Zou X, Gao Y, Huang X. 2008. New oxidant used for the post-column derivatization determination of Malachite Green and Leucomalachite Green residues in cultured aquatic products by high-performance liquid chromatography. J Chromatogr A 1203:21-26.

Loper BL, Anderson KA. 2003. Determination of pyrethrin and pyrethroid pesticides in urine and water matrixes by liquid chromatography with diode array detection. J AOAC Int 86:1236-1240.

Lowry LK, Tolos WP, Boeniger MF, Nony CR, Bowman MC. 1980. Chemical monitoring of urine from workers potentially exposed to benzidine-derived azo dyes. Toxicol Lett 7:29-36.

Madhavan ND, Naidu KA. 1995. Polycyclic aromatic hydrocarbons in placenta, maternal blood, umbilical cord blood and milk of Indian women. Hum Exp Toxicol 14:503-506.

Magagnotti C, Orsi F, Bagnati R, Celli N, Rotilio D, Fanelli R, et al. 2000. Effect of diet on serum albumin and hemoglobin adducts of 2-amino-1-methyl-6-phenylimidazo[4,5-b]pyridine (PhIP) in humans. Int J Cancer 88:1-6.

Maître A, Berode M, Perdrix A, Romazini S, Savolainen H. 1993. Biological monitoring of occupational exposure to toluene diisocyanate. Int Arch Occup Environ Health 65:97-100.

Manabe S, Wada O. 1990. Identification of carcinogenic tryptophan pyrolysis products in human bile by high-performance liquid chromatography. Environ Mol Mutagen 15:229-235.

Manabe S, Suzuki M, Kusano E, Wada O, Asano Y. 1992. Elevation of levels of carcinogenic tryptophan pyrolysis products in plasma and red blood cells of patients with uremia. Clin Nephrol 37:28-33.

Mannila A, Kumpulainen E, Lehtonen M, Heikkinen M, Laisalmi M, Salo T, et al. 2007. Plasma and cerebrospinal fluid concentrations of indomethacin in children after intravenous administration. J Clin Pharmacol 47:94-100.

Margalho C, de Boer D, Gallardo E, Barroso M, Vieira DN. 2005. Determination of furosemide in whole blood using SPE and GC-EI-MS. J Anal Toxicol 29:309-313.

Martinez MA, Ballesteros S, Almarza E, Sanchez de la Torre C, Bua S. 2003. Acute nitrobenzene poisoning with severe associated methemoglobinemia: identification in whole blood by GC-FID and GC-MS. J Anal Toxicol 27:221-225.

McCauley LA, Lasarev M, Muniz J, Nazar Stewart V, Kisby G. 2008. Analysis of pesticide exposure and DNA damage in immigrant farmworkers. J Agromedicine 13:237-246.

McDonald JG, Matthew S, Auchus RJ. 2011. Steroid profiling by gas chromatography-mass spectrometry and high performance liquid chromatography-mass spectrometry for adrenal diseases. Horm Cancer 2:324-332.

Means JC, Olsen PD, Schoffers E. 2003. Development of an isotope dilution liquid chromatography/tandem mass spectrometry detection method for DNA adducts of selected aromatic amines. J Am Soc Mass Spectrom 14:1057-1066.

Mendas G, Vuletic M, Galic N, Drevenkar V. 2012. Urinary metabolites as biomarkers of human exposure to atrazine: Atrazine mercapturate in agricultural workers. Toxicol Lett 210:174-181.

Mendes P, Liang Q, Frost-Pineda K, Munjal S, Walk RA, Roethig HJ. 2009. The relationship between smoking machine derived tar yields and biomarkers of exposure in adult cigarette smokers in the US. Regul Toxicol Pharmacol 55:17-27.

Michail K, Moneeb MS. 2011. Determination of methotrexate and indomethacin in urine using SPE-LC-DAD after derivatization. J Pharm Biomed Anal 55:317-324.

Miekisch W, Schubert JK, Vagts DA, Geiger K. 2001. Analysis of volatile disease markers in blood. Clin Chem 47:1053-1060.

Mieth M, Kischkel S, Schubert JK, Hein D, Miekisch W. 2009. Multibed needle trap devices for on site sampling and preconcentration of volatile breath biomarkers. Anal Chem 81:5851-5857.

Mikes P, Korinek M, Linhart I, Krouzelka J, Dabrowska L, Stransky V, et al. 2010. Urinary N3 adenine DNA adducts in humans occupationally exposed to styrene. Toxicol Lett 197:183-187.

Miksa IR, Cummings MR, Poppenga RH. 2005. Multi-residue determination of anti-inflammatory analgesics in sera by liquid chromatography--mass spectrometry. J Anal Toxicol 29:95-104.

Minet E, Cheung F, Errington G, Sterz K, Scherer G. 2011. Urinary excretion of the acrylonitrile metabolite 2-cyanoethylmercapturic acid is correlated with a variety of biomarkers of tobacco smoke exposure and consumption. Biomarkers 16:89-96.

Minoia C, Turci R, Sottani C, Schiavi A, Perbellini L, Angeleri S, et al. 1998. Application of high performance liquid chromatography/tandem mass spectrometry in the environmental and biological monitoring of health care personnel occupationally exposed to cyclophosphamide and ifosfamide. Rapid Commun Mass Spectrom 12:1485-1493.

Mistri HN, Jangid AG, Sanyal M, Shrivastav P. 2007. Electrospray ionization LC-MS/MS validated method to quantify griseofulvin in human plasma and its application to bioequivalence study. J Chromatogr B Analyt Technol Biomed Life Sci 850:318-326.

Mitrowska K, Posyniak A, Zmudzki J. 2005. Determination of malachite green and leucomalachite green in carp muscle by liquid chromatography with visible and fluorescence detection. J Chromatogr A 1089:187-192.

Molina-Garcia L, Fernandez-de Cordova ML, Ruiz-Medina A. 2010. Sensitive determination of indomethacin in pharmaceuticals and urine by sequential injection analysis and optosensing. J AOAC Int 93:1443-1449.

Moretti M, Bonfiglioli R, Feretti D, Pavanello S, Mussi F, Grollino MG, et al. 2011. A study protocol for the evaluation of occupational mutagenic/carcinogenic risks in subjects exposed to antineoplastic drugs: a multicentric project. BMC Public Health 11:195.

Msagati TA, Nindi MM. 2006. The use of liquid membranes in the multi-residue extraction of stilbenes in a variety of biological matrices and their detection with LC-ES-MS. Ann Chim 96:635-646.

Mullins ME, Hammett-Stabler CA. 1998. Intoxication with nitromethane-containing fuels: don't be "fueled" by the creatinine. J Toxicol Clin Toxicol 36:315-320.

Muñoz K, Blaszkewicz M, Degen GH. 2009. Simultaneous analysis of ochratoxin A and its major metabolite ochratoxin alpha in plasma and urine for an advanced biomonitoring of the mycotoxin. J Chromatogr B Analyt Technol Biomed Life Sci.

Murray EB, Edwards JW. 1999. Micronuclei in peripheral lymphocytes and exfoliated urothelial cells of workers exposed to 4,4'-methylenebis-(2-chloroaniline) (MOCA). Mutat Res 446:175-180.

Murray S, Boobis AR. 1991. Combined assay for phenacetin and paracetamol in plasma using capillary column gas chromatography-negative-ion mass spectrometry. J Chromatogr 568:341-350.

Musshoff F, Junker H, Madea B. 2002. Simple determination of 22 organophosphorous pesticides in human blood using headspace solid-phase microextraction and gas chromatography with mass spectrometric detection. J Chromatogr Sci 40:29-34.

Muth P, Metz R, Siems B, Bolten WW, Vergin H. 1996. Sensitive determination of nitrofurantoin in human plasma and urine by high-performance liquid chromatography. J Chromatogr A 729:251-258.

Myers SR, Spinnato JA, Pinorini-Godly MT, Cook C, Boles B, Rodgers GC. 1996. Characterization of 4-aminobiphenyl-hemoglobin adducts in maternal and fetal blood-samples. J Toxicol Environ Health 47:553-566.

Nakata H, Nasu T, Abe S, Kitano T, Fan Q, Li W, et al. 2005. Organochlorine contaminants in human adipose tissues from China: mass balance approach for estimating historical Chinese exposure to DDTs. Environ Sci Technol 39:4714-4720.

Neal MS, Zhu J, Foster WG. 2008. Quantification of benzo[a]pyrene and other PAHs in the serum and follicular fluid of smokers versus non-smokers. Reprod Toxicol 25:100-106.

Neumann HG, Albrecht O, van Dorp C, Zwirner-Baier I. 1995. Macromolecular adducts caused by environmental chemicals. Clin Chem 41:1835-1840.

Nicolau I, Tian L, Menzies D, Ostiguy G, Pai M. 2012. Point-of-care urine tests for smoking status and isoniazid treatment monitoring in adult patients. PLoS ONE 7:e45913.

NIOSH. 1993. Manual of analytical methods: benzidine in urine (screening test). Method 8304. Available: http://www.cdc.gov/niosh/docs/2003-154/pdfs/8304.pdf [accessed 8 April 2014].

NIOSH. 1994a. Manual of analytical methods: benzidine in urine. Method 8306. Available: http://www.cdc.gov/niosh/docs/2003-154/pdfs/8306.pdf [accessed 8 April 2014].

NIOSH. 1994b. Manual of analytical methods: MBOC in urine. Method 8302. Available: http://www.cdc.gov/niosh/docs/2003-154/pdfs/8302.pdf [accessed 8 April 2014].

NIOSH. 2003. Manual of analytical methods: triazine herbicides and their metabolites in urine. Method 8315. Available: http://www.cdc.gov/niosh/docs/2003-154/pdfs/8315.pdf [accessed 8 April 2014].

NLM. 2004. TOXNET: Toxicology Data Network. ((Toxnet) TDN, ed):National Library of Medicine.

NLM. 2011. Hazardous substances data bank (HSDB). Available: http://toxnet.nlm.nih.gov/cgi-bin/sis/htmlgen?HSDB [accessed 8 April 2014].

NLM. 2013. Household Products Database. Available: http://householdproducts.nlm.nih.gov/ [accessed 7 April 2014].

NLM. 2014. PubChem. Available: http://pubchem.ncbi.nlm.nih.gov/ [accessed 8 April 2014].

NTP. 1978a. Technical report 53. Available: http://ntp.niehs.nih.gov/ntp/htdocs/LT_rpts/tr053.pdf [accessed 8 April 2014].

NTP. 1978b. Technical report 108. Available: http://ntp.niehs.nih.gov/ntp/htdocs/LT_rpts/tr108.pdf [accessed 8 April 2014].

NTP. 1978c. Technical report 91. Available: http://ntp.niehs.nih.gov/ntp/htdocs/LT_rpts/tr091.pdf [accessed 8 April 2014].

NTP. 1979. NTP Technical Series No. 146; bioassay of nithiazide for possible carcinogenicity CAS No. 139-94-6 National Cancer Institute Carcinogenesis Technical Report Series No. 146 (Natl Toxicol Program Tech Rep Ser).

NTP. 1988. Technical report 337. Available: http://ntp.niehs.nih.gov/ntp/htdocs/LT_rpts/tr337.pdf [accessed 8 April 2014].

NTP. 1989. Technical report 356. Available: http://ntp.niehs.nih.gov/ntp/htdocs/LT_rpts/tr356.pdf [accessed 8 April 2014].

NTP. 1992. Technical report 412. Available: http://ntp.niehs.nih.gov/ntp/htdocs/LT_rpts/tr412.pdf [accessed 8 April 2014].

NTP. 2004. NTP technical report on the toxicity studies of malachite green chloride and leucomalachite green (CAS Nos. 569-64-2 and 129-73-7) administered in feed to F344/N rats and B6C3F1 mice. Toxic Rep Ser:1-F10.

NTP. 2005. 11th Report on Carcinogens. Available: http://ntp.niehs.nih.gov/ntp/roc/toc11.htm [accessed 7 April 2014].

NTP. 2011. 12th Report on Carcinogens. Available: http://ntp.niehs.nih.gov/ntp/roc/twelfth/roc12.pdf [accessed 7 April 2014].

Ogawa M, Oyama T, Isse T, Yamaguchi T, Murakami T, Endo Y, et al. 2006. Hemoglobin adducts as a marker of exposure to chemical substances, especially PRTR class I designated chemical substances. J Occup Health 48:314-328.

OSHA. 2012. 1,3-butadiene. Available: http://www.osha.gov/SLTC/butadiene [accessed 7 April 2014].

Owen JA, Nakatsu SL, Condra M, Surridge DH, Fenemore J, Morales A. 1985. Sub-nanogram analysis of yohimbine and related compounds by high-performance liquid chromatography. J Chromatogr 342:333-340.

Pacifici GM. 2006. Placental transfer of antibiotics administered to the mother: a review. Int J Clin Pharmacol Ther 44:57-63.

Panuwet P, Restrepo PA, Magsumbol M, Jung KY, Montesano MA, Needham LL, et al. 2010. An improved high-performance liquid chromatography-tandem mass spectrometric method to measure atrazine and its metabolites in human urine. J Chromatogr B Analyt Technol Biomed Life Sci 878:957-962.

Patel G, Agrawal YK. 2003. Separation and trace estimation of benzidine and its macromolecular adducts using supercritical fluid chromatography. J Chromatogr B Analyt Technol Biomed Life Sci 795:157-165.

Peloquin CA. 2002. Therapeutic drug monitoring in the treatment of tuberculosis. Drugs 62:2169-2183.

Peluso M, Neri M, Margarino G, Mereu C, Munnia A, Ceppi M, et al. 2004. Comparison of DNA adduct levels in nasal mucosa, lymphocytes and bronchial mucosa of cigarette smokers and interaction with metabolic gene polymorphisms. Carcinogenesis 25:2459-2465.

Peluso M, Airoldi L, Munnia A, Colombi A, Veglia F, Autrup H, et al. 2008. Bulky DNA adducts, 4-aminobiphenyl-haemoglobin adducts and diet in the European Prospective Investigation into Cancer and Nutrition (EPIC) prospective study. Br J Nutr 100:489-495.

Perbellini L, Princivalle A, Cerpelloni M, Caprini A. 2003a. [Biological monitoring of occupational exposure to acrylonitrile]. G Ital Med Lav Ergon 25 Suppl:41-42.

Perbellini L, Princivalle A, Cerpelloni M, Pasini F, Brugnone F. 2003b. Comparison of breath, blood and urine concentrations in the biomonitoring of environmental exposure to 1,3-butadiene, 2,5-dimethylfuran, and benzene. Int Arch Occup Environ Health 76:461-466.

Pirali B, Negri S, Chytiris S, Perissi A, Villani L, La Manna L, et al. 2009. Perfluorooctane sulfonate and perfluorooctanoic acid in surgical thyroid specimens of patients with thyroid diseases. Thyroid 19:1407-1412.

Poli D, Manini P, Andreoli R, Franchini I, Mutti A. 2005. Determination of dichloromethane, trichloroethylene and perchloroethylene in urine samples by headspace solid phase microextraction gas chromatography-mass spectrometry. J Chromatogr B Analyt Technol Biomed Life Sci 820:95-102.

Pons G, Rey E, Richard MO, Vauzelle F, Francoual C, Moran C, et al. 1990. Nitrofurantoin excretion in human milk. Dev Pharmacol Ther 14:148-152.

Poullain-Termeau S, Crauste-Manciet S, Brossard D, Muhamed S, Nicolaos G, Farinotti R, et al. 2008. Effect of oil-in-water submicron emulsion surface charge on oral absorption of a poorly water-soluble drug in rats. Drug Deliv 15:503-514.

Preston A, Fodey T, Douglas A, Elliott CT. 2009. Monoclonal antibody development for acrylamide-adducted human haemoglobin; a biomarker of dietary acrylamide exposure. J Immunol Methods 341:19-29.

Prieto-Castello MJ, Cardona A, Marhuenda D, Roel JM, Corno A. 2010. Use of the CYP2E1 genotype and phenotype for the biological monitoring of occupational exposure to styrene. Toxicol Lett 192:34-39.

Ramesh A, Ravi PE. 2004. Negative ion chemical ionization-gas chromatographic-mass spectrometric determination of residues of different pyrethroid insecticides in whole blood and serum. J Anal Toxicol 28:660-666.

Reeuwijk HJ, Tjaden UR, van der Greef J. 1992. Simultaneous determination of furosemide and amiloride in plasma using high-performance liquid chromatography with fluorescence detection. J Chromatogr 575:269-274.

Reistad R, Rossland OJ, Latva-Kala KJ, Rasmussen T, Vikse R, Becher G, et al. 1997. Heterocyclic aromatic amines in human urine following a fried meat meal. Food Chem Toxicol 35:945-955.

Reska M, Ochsmann E, Kraus T, Schettgen T. 2010. Accurate quantification of mercapturic acids of styrene (PHEMAs) in human urine with direct sample injection using automated column-switching high-performance liquid chromatography coupled with tandem mass spectrometry. Anal Bioanal Chem 397:3563-3574.

Riano S, Alcudia-Leon MC, Lucena R, Cardenas S, Valcarcel M. 2012. Determination of non-steroidal anti-inflammatory drugs in urine by the combination of stir membrane liquid-liquid-liquid microextraction and liquid chromatography. Anal Bioanal Chem 403:2583-2589.

Richter E, Rösler S, Scherer G, Gostomzyk JG, Grübl A, Krämer U, et al. 2001. Haemoglobin adducts from aromatic amines in children in relation to area of residence and exposure to environmental tobacco smoke. Int Arch Occup Environ Health 74:421-428.

Riedel K, Scherer G, Engl J, Hagedorn H-W, Tricker AR. 2006. Determination of three carcinogenic aromatic amines in urine of smokers and nonsmokers. J Anal Toxicol 30:187-195.

Rieder J, Lirk P, Ebenbichler C, Gruber G, Prazeller P, Lindinger W, et al. 2001. Analysis of volatile organic compounds: possible applications in metabolic disorders and cancer screening. Wien Klin Wochenschr 113:181-185.

Riederer AM, Bartell SM, Barr DB, Ryan PB. 2008. Diet and nondiet predictors of urinary 3-phenoxybenzoic acid in NHANES 1999-2002. Environ Health Perspect 116:1015-1022.

Rihs HP, Triebig G, Werner P, Rabstein S, Heinze E, Pesch B, et al. 2008. Association between genetic polymorphisms in styrene-metabolizing enzymes and biomarkers in styrene-exposed workers. J Toxicol Environ Health A 71:866-873.

Ritieni A, Santini A, Mussap M, Ferracane R, Bosco P, Gazzolo D, et al. 2010. Simultaneous determination of mycotoxins in biological fluids by LC-MS/MS. Front Biosci (Elite Ed) 2:151-158.

Roberts KP, Jankowiak R, Small GJ. 2001. High-performance liquid chromatography interfaced with fluorescence line-narrowing spectroscopy for on-line analysis. Anal Chem 73:951-956.

Rodgers RM, Garvie-Gould C, Scott KF, Milam DF, Lynn RK. 1983. Metabolism, distribution, and excretion of the carcinogenic aromatic amine, 3,3'-dimethoxybenzidine in the rat. Formation of mutagenic urinary and biliary metabolites. Drug Metab Dispos 11:293-300.

Roethig HJ, Munjal S, Feng S, Liang Q, Sarkar M, Walk RA, et al. 2009. Population estimates for biomarkers of exposure to cigarette smoke in adult U.S. cigarette smokers. Nicotine Tob Res 11:1216-1225.

Roosens L, D'Hollander W, Bervoets L, Reynders H, Van Campenhout K, Cornelis C, et al. 2010. Brominated flame retardants and perfluorinated chemicals, two groups of persistent contaminants in Belgian human blood and milk. Environ Pollut 158:2546-2552.

Rosenberg C, Nikkila K, Henriks-Eckerman ML, Peltonen K, Engstrorm K. 2002. Biological monitoring of aromatic diisocyanates in workers exposed to thermal degradation products of polyurethanes. J Environ Monit 4:711-716.

Rosner W, Hankinson SE, Sluss PM, Vesper HW, Wierman ME. 2013. Challenges to the measurement of estradiol: an endocrine society position statement. J Clin Endocrinol Metab 98:1376-1387.

Rossella F, Campo L, Pavanello S, Kapka L, Siwinska E, Fustinoni S. 2009. Urinary polycyclic aromatic hydrocarbons and monohydroxy metabolites as biomarkers of exposure in coke oven workers. Occup Environ Med 66:509-516.

Rubies A, Cabrera A, Centrich F. 2007. Determination of synthetic hormones in animal urine by high-performance liquid chromatography/mass spectrometry. J AOAC Int 90:626-632.

Rudel RA, Camann DE, Spengler JD, Korn LR, Brody JG. 2003. Phthalates, alkylphenols, pesticides, polybrominated diphenyl ethers, and other endocrine-disrupting compounds in indoor air and dust. Environ Sci Technol 37:4543-4553.

Rudel RA, Attfield KR, Schifano JN, Brody JG. 2007. Chemicals causing mammary gland tumors in animals signal new directions for epidemiology, chemicals testing, and risk assessment for breast cancer prevention. Cancer 109:2635-2666.

Rudel RA, Fenton SE, Ackerman JM, Euling SY, Makris SL. 2011. Environmental exposures and mammary gland development: state of the science, public health implications, and research recommendations. Environ Health Perspect 119:1053-1061.

Rudge CV, Sandanger T, Rollin HB, Calderon IM, Volpato G, Silva JL, et al. 2012. Levels of selected persistent organic pollutants in blood from delivering women in seven selected areas of Sao Paulo State, Brazil. Environ Int 40:162-169.

Rueff J, Teixeira JP, Santos LS, Gaspar JF. 2009. Genetic effects and biotoxicity monitoring of occupational styrene exposure. Clin Chim Acta 399:8-23.

Rundle A, Tang D, Hibshoosh H, Schnabel F, Kelly A, Levine R, et al. 2002. Molecular epidemiologic studies of polycyclic aromatic hydrocarbon-DNA adducts and breast cancer. Environ Mol Mutagen 39:201-207.

Sakai T, Morita Y, Wakui C. 2002. Biological monitoring of workers exposed to dichloromethane, using head-space gas chromatography. J Chromatogr B Analyt Technol Biomed Life Sci 778:245-250.

Salem AA, Mossa HA, Barsoum BN. 2006. Application of nuclear magnetic resonance spectroscopy for quantitative analysis of miconazole, metronidazole and sulfamethoxazole in pharmaceutical and urine samples. J Pharm Biomed Anal 41:654-661.

Salem AA, Mossa HA. 2012. Method validation and determinations of levofloxacin, metronidazole and sulfamethoxazole in an aqueous pharmaceutical, urine and blood plasma samples using quantitative nuclear magnetic resonance spectrometry. Talanta 88:104-114.

Sanchez Mdel N, Garcia EH, Pavon JL, Cordero BM. 2012. Fast analytical methodology based on mass spectrometry for the determination of volatile biomarkers in saliva. Anal Chem 84:379-385.

Sapkota A, Halden RU, Dominici F, Groopman JD, Buckley TJ. 2006. Urinary biomarkers of 1,3-butadiene in environmental settings using liquid chromatography isotope dilution tandem mass spectrometry. Chem Biol Interact 160:70-79.

Sarkar M, Stabbert R, Kinser RD, Oey J, Rustemeier K, von Holt K, et al. 2006. CYP1A2 and NAT2 phenotyping and 3-aminobiphenyl and 4-aminobiphenyl hemoglobin adduct levels in smokers and non-smokers. Toxicol Appl Pharmacol 213:198-206.

Saugy M, Meuwly P, Munafo A, Rivier L. 1991. Rapid high-performance liquid chromatographic determination with fluorescence detection of furosemide in human body fluids and its confirmation by gas chromatography-mass spectrometry. J Chromatogr 564:567-578.

Schaut A, De Saeger S, Sergent T, Schneider YJ, Larondelle Y, Pussemier L, et al. 2008. Liquid chromatographic methods for biotransformation studies of ochratoxin A. Biomed Chromatogr 22:1013-1020.

Schecter A, Lucier GW, Cunningham ML, Abdo KM, Blumenthal G, Silver AG, et al. 2004. Human consumption of methyleugenol and its elimination from serum. Environ Health Perspect 112:678-680.

Scherer G, Urban M, Hagedorn H-W, Serafin R, Feng S, Kapur S, et al. 2010. Determination of methyl-, 2-hydroxyethyl- and 2-cyanoethylmercapturic acids as biomarkers of exposure to alkylating agents in cigarette smoke. J Chromatogr B Analyt Technol Biomed Life Sci 878:2520-2528.

Schettgen T, Broding HC, Angerer J, Drexler H. 2002. Hemoglobin adducts of ethylene oxide, propylene oxide, acrylonitrile and acrylamide-biomarkers in occupational and environmental medicine. Toxicol Lett 134:65-70.

Schettgen T, Kütting B, Hornig M, Beckmann MW, Weiss T, Drexler H, et al. 2004. Trans-placental exposure of neonates to acrylamide--a pilot study. Int Arch Occup Environ Health 77:213-216.

Schettgen T, Musiol A, Kraus T. 2008. Simultaneous determination of mercapturic acids derived from ethylene oxide (HEMA), propylene oxide (2-HPMA), acrolein (3-HPMA), acrylamide (AAMA) and N,N-dimethylformamide (AMCC) in human urine using liquid chromatography/tandem mass spectrometry. Rapid Commun Mass Spectrom 22:2629-2638.

Schettgen T, Musiol A, Alt A, Ochsmann E, Kraus T. 2009. A method for the quantification of biomarkers of exposure to acrylonitrile and 1,3-butadiene in human urine by column-switching liquid chromatography-tandem mass spectrometry. Anal Bioanal Chem 393:969-981.

Schettgen T, Heinrich K, Kraus T, Gube M. 2010. Determination of 2,5-toluylenediamine (2,5-TDA) and aromatic amines in urine after personal application of hair dyes: kinetics and doses. Arch Toxicol.

Schettgen T, Müller J, Fromme H, Angerer J. 2010. Simultaneous quantification of haemoglobin adducts of ethylene oxide, propylene oxide, acrylonitrile, acrylamide and glycidamide in human blood by isotope-dilution GC/NCI-MS/MS. J Chromatogr B Analyt Technol Biomed Life Sci 878:2467-2473.

Schmidt K, Stachel C, Gowik P. 2008. Development and in-house validation of an LC-MS/MS method for the determination of stilbenes and resorcylic acid lactones in bovine urine. Anal Bioanal Chem 391:1199-1210.

Scott PM. 2005. Biomarkers of human exposure to ochratoxin A. Food Addit Contam 22 Suppl 1:99-107.

Seidel A, Dahmann D, Krekeler H, Jacob J. 2002. Biomonitoring of polycyclic aromatic compounds in the urine of mining workers occupationally exposed to diesel exhaust. Int J Hyg Environ Health 204:333-338.

Seifart HI, Gent WL, Parkin DP, van Jaarsveld PP, Donald PR. 1995. High-performance liquid chromatographic determination of isoniazid, acetylisoniazid and hydrazine in biological fluids. J Chromatogr B, Biomed Appl 674:269-275.

Sennbro CJ, Lindh CH, Tinnerberg H, Welinder H, Littorin M, Jönsson BAG. 2004. Biological monitoring of exposure to toluene diisocyanate. Scand J Work Environ Health 30:371-378.

Sessink PJ, Wittenhorst BC, Anzion RB, Bos RP. 1997. Exposure of pharmacy technicians to antineoplastic agents: reevaluation after additional protective measures. Arch Environ Health 52:240-244.

Seyler TH, Bernert JT. 2011. Analysis of 4-aminobiphenyl in smoker's and nonsmoker's urine by tandem mass spectrometry. Biomarkers 16:212-221.

Shan G, Wengatz I, Stoutamire DW, Gee SJ, Hammock BD. 1999. An enzyme-linked immunosorbent assay for the detection of esfenvalerate metabolites in human urine. Chem Res Toxicol 12:1033-1041.

Shen S, Zhang F, Zeng S, Zheng J. 2009. An approach based on liquid chromatography/electrospray ionization-mass spectrometry to detect diol metabolites as biomarkers of exposure to styrene and 1,3-butadiene. Anal Biochem 386:186-193.

Shih WC, Chen MF, Huang CC, Uang SN, Shih TS, Liou SH, et al. 2007. Simultaneous analysis of urinary 4,4'-methylenebis(2-chloroaniline) and N-acetyl 4,4'-methylenebis(2-chloroaniline) using solid-phase extraction and liquid chromatography/tandem mass spectrometry. Rapid Commun Mass Spectrom 21:4073-4078.

Shin H-S, Ahn H-S. 2006. Determination of the propylene oxide-hemoglobin adduct by gas chromatography-electron impact ionization mass spectrometry. J Mass Spectrom 41:802-809.

Singh N, Golani A, Patel Z, Maitra A. 2008. Transfer of isoniazid from circulation to breast milk in lactating women on chronic therapy for tuberculosis. Br J Clin Pharmacol 65:418-422.

Singh VK, Patel DK, Ram S, Mathur N, Siddiqui MKJ, Behari JR. 2008. Blood levels of polycyclic aromatic hydrocarbons in children of Lucknow, India. Arch Environ Contam Toxicol 54:348-354.

Singh VK, Singh J, Anand M, Kumar P, Patel DK, Krishna Reddy MM, et al. 2008. Comparison of polycyclic aromatic hydrocarbon levels in placental tissues of Indian women with full- and preterm deliveries. Int J Hyg Environ Health 211:639-647.

Skipper PL, Tannenbaum SR, Ross RK, Yu MC. 2003. Nonsmoking-related arylamine exposure and bladder cancer risk. Cancer Epidemiol Biomarkers Prev 12:503-507.

Smith S, Burden H, Persad R, Whittington K, de Lacy Costello B, Ratcliffe NM, et al. 2008. A comparative study of the analysis of human urine headspace using gas chromatography-mass spectrometry. J Breath Res 2:037022.

Smith TJ, Bois FY, Lin YS, Brochot C, Micallef S, Kim D, et al. 2008. Quantifying heterogeneity in exposure-risk relationships using exhaled breath biomarkers for 1,3-butadiene exposures. J Breath Res 2:037018.

Sörgel F, Weissenbacher R, Kinzig-Schippers M, Hofmann A, Illauer M, Skott A, et al. 2002. Acrylamide: increased concentrations in homemade food and first evidence of its variable absorption from food, variable metabolism and placental and breast milk transfer in humans. Chemotherapy 48:267-274.

Sottani C, Tranfo G, Bettinelli M, Faranda P, Spagnoli M, Minoia C. 2004. Trace determination of anthracyclines in urine: a new high-performance liquid chromatography/tandem mass spectrometry method for assessing exposure of hospital personnel. Rapid Commun Mass Spectrom 18:2426-2436.

Sottani C, Rinaldi P, Leoni E, Poggi G, Teragni C, Delmonte A, et al. 2008. Simultaneous determination of cyclophosphamide, ifosfamide, doxorubicin, epirubicin and daunorubicin in human urine using high-performance liquid chromatography/electrospray ionization tandem mass spectrometry: bioanalytical method validation. Rapid Commun Mass Spectrom 22:2645-2659.

Stanczyk FZ, Clarke NJ. 2010. Advantages and challenges of mass spectrometry assays for steroid hormones. J Steroid Biochem Mol Biol 121:491-495.

Stone BG, Besse TJ, Duane WC, Evans CD, DeMaster EG. 1993. Effect of regulating cholesterol biosynthesis on breath isoprene excretion in men. Lipids 28:705-708.

Sugiura S, Nakanishi H, Asano M, Hashida T, Tanimura M, Hama T, et al. 2011. Multicenter study for environmental and biological monitoring of occupational exposure to cyclophosphamide in Japan. J Oncol Pharm Pract 17:20-28.

Sullins AK, Abdel-Rahman SM. 2013. Pharmacokinetics of antibacterial agents in the CSF of children and adolescents. Paediatr Drugs 15:93-117.

Sun H, Wang H, Ge X. 2012. Simultaneous determination of the combined drugs of ceftriaxone sodium, metronidazole, and levofloxacin in human urine by high-performance liquid chromatography. J Clin Lab Anal 26:486-492.

Sun X, Karlsson A, Bartsch H, Nair J. 2006. New ultrasensitive 32P-postlabelling method for the analysis of 3,N4-etheno-2'-deoxycytidine in human urine. Biomarkers 11:329-340.

Sundström M, Ehresman DJ, Bignert A, Butenhoff JL, Olsen GW, Chang S-C, et al. 2011. A temporal trend study (1972-2008) of perfluorooctanesulfonate, perfluorohexanesulfonate, and perfluorooctanoate in pooled human milk samples from Stockholm, Sweden. Environ Int 37:178-183.

Suyagh M, Collier PS, Millership JS, Iheagwaram G, Millar M, Halliday HL, et al. 2011. Metronidazole population pharmacokinetics in preterm neonates using dried blood-spot sampling. Pediatrics 127:e367-374.

Swenberg JA, Bordeerat NK, Boysen G, Carro S, Georgieva NI, Nakamura J, et al. 2011. 1,3-Butadiene: Biomarkers and application to risk assessment. Chem Biol Interact 192:150-154.

Szaniszló J, Ungváry G. 2001. Polycyclic aromatic hydrocarbon exposure and burden of outdoor workers in Budapest. J Toxicol Environ Health A 62:297-306.

Takayasu T, Ohshima T, Kondo T. 2001. Rapid analysis of pesticide components, xylene, o-dichlorbenzene, cresol and dichlorvos, in blood and urine by pulse heating-gas chromatography-mass spectrometry. Leg Med (Tokyo) 3:157-161.

Tanabe S, Kunisue T. 2007. Persistent organic pollutants in human breast milk from Asian countries. Environ Pollut 146:400-413.

Tas AC, van der Greef J, ten Noever de Brauw MC, Plomp TA, Maes RA, Hohn M, et al. 1986. LC/MS determination of bromazepam, clopenthixol, and reserpine in serum of a non-fatal case of intoxication. J Anal Toxicol 10:46-48.

Taucher J, Hansel A, Jordan A, Fall R, Futrell JH, Lindinger W. 1997. Detection of isoprene in expired air from human subjects using proton-transfer-reaction mass spectrometry. Rapid Commun Mass Spectrom 11:1230-1234.

Taylor NF. 2006. Urinary steroid profiling. Methods Mol Biol 324:159-175.

Thier R, Lewalter J, Selinski S, Bolt HM. 2001. Biological monitoring in workers in a nitrobenzene reduction plant: haemoglobin versus serum albumin adducts. Int Arch Occup Environ Health 74:483-488.

Thomas S, Sutton A, Garg U. 2010. Quantitation of indomethacin in serum and plasma using gas chromatography-mass spectrometry (GC-MS). Methods Mol Biol 603:297-305.

Thrall KD, Callahan PJ, Weitz KK, Edwards JA, Brinkman MC, Kenny DV. 2001. Design and evaluation of a breath-analysis system for biological monitoring of volatile compound. AIHAJ 62:28-35.

Tompkins EM, Jones DJ, Lamb JH, Marsden DA, Farmer PB, Brown K. 2008. Simultaneous detection of five different 2-hydroxyethyl-DNA adducts formed by ethylene oxide exposure, using a high-performance liquid chromatography/electrospray ionisation tandem mass spectrometry assay. Rapid Commun Mass Spectrom 22:19-28.

Toriba A, Kuramae Y, Chetiyanukornkul T, Kizu R, Makino T, Nakazawa H, et al. 2003. Quantification of polycyclic aromatic hydrocarbons (PAHs) in human hair by HPLC with fluorescence detection: a biological monitoring method to evaluate the exposure to PAHs. Biomed Chromatogr 17:126-132.

Toriba A, Kitaoka H, Dills RL, Mizukami S, Tanabe K, Takeuchi N, et al. 2007. Identification and quantification of 1-nitropyrene metabolites in human urine as a proposed biomarker for exposure to diesel exhaust. Chem Res Toxicol 20:999-1007.

Tran NL, Barraj LM, Murphy MM, Bi X. 2010. Dietary acrylamide exposure and hemoglobin adducts--National Health and Nutrition Examination Survey (2003-04). Food Chem Toxicol 48:3098-3108.

Tsai IL, Liu HY, Kuo PH, Wang JY, Shen LJ, Kuo CH. 2011. Quantitative determination of isoniazid in biological samples by cation-selective exhaustive injection-sweeping-micellar electrokinetic chromatography. Anal Bioanal Chem 401:2205-2214.

Tsatsakis AM, Barbounis MG, Kavalakis M, Kokkinakis M, Terzi I, Tzatzarakis MN. 2010. Determination of dialkyl phosphates in human hair for the biomonitoring of exposure to organophosphate pesticides. J Chromatogr B Analyt Technol Biomed Life Sci 878:1246-1252.

Turci R, Minoia C, Sottani C, Coghi R, Severi P, Castriotta C, et al. 2011. Occupational exposure to antineoplastic drugs in seven Italian hospitals: the effect of quality assurance and adherence to guidelines. J Oncol Pharm Pract 17:320-332.

Turner C, Spanel P, Smith D. 2006. A longitudinal study of breath isoprene in healthy volunteers using selected ion flow tube mass spectrometry (SIFT-MS). Physiol Meas 27:13-22.

Turnipseed SB, Andersen WC, Roybal JE. 2005. Determination and confirmation of malachite green and leucomalachite green residues in salmon using liquid chromatography/mass spectrometry with no-discharge atmospheric pressure chemical ionization. J AOAC Int 88:1312-1317.

US EPA. 2010a. Dyes derived from benzidine and its congeners. Available: http://www.epa.gov/oppt/existingchemicals/pubs/actionplans/DCB Action Plan_06232010.noheader.pdf [accessed 7 April 2014].

US EPA. 2010b. Exposure assessment tools and models: source ranking database. Available: http://www.epa.gov/opptintr/exposure/pubs/srd.htm [accessed 7 April 2014].

US EPA. 2011. Toluene diisocyanate (TDI) and related compounds action plan [RIN 2070-ZA14]. Available: http://www.epa.gov/oppt/existingchemicals/pubs/actionplans/tdi.pdf [accessed 7 April 2014].

US EPA. 2012. TSCA work plan chemicals. Available: http://www.epa.gov/oppt/existingchemicals/pubs/Work_Plan_Chemicals_Web_Final.pdf [accessed 8 April 2014].

US EPA. 2013a. Toluene diiscoyanate (TDI) action plan summary. Available: http://www.epa.gov/opptintr/existingchemicals/pubs/actionplans/tdi.html [accessed 8 April 2014].

US EPA. 2013b. Long-chain perfluorinated chemicals (PFCs) action plan summary. Available: http://www.epa.gov/opptintr/existingchemicals/pubs/actionplans/pfcs.html [accessed 8 April 2014].

US FDA. 2013. "Everything" Added to Food in the United States (EAFUS): A food additive database Available: http://www.fda.gov/Food/IngredientsPackagingLabeling/FoodAdditivesIngredients/ucm115326.htm [accessed 7 April 2014].

Ushiyama H, Wakabayashi K, Hirose M, Itoh H, Sugimura T, Nagao M. 1991. Presence of carcinogenic heterocyclic amines in urine of healthy volunteers eating normal diet, but not of inpatients receiving parenteral alimentation. Carcinogenesis 12:1417-1422.

Vacek PM, Albertini RJ, Sram RJ, Upton P, Swenberg JA. 2010. Hemoglobin adducts in 1,3-butadiene exposed Czech workers: female-male comparisons. Chem Biol Interact 188:668-676.

Van Hemelrijck MJ, Michaud DS, Connolly GN, Kabir Z. 2009. Secondhand smoking, 4-aminobiphenyl, and bladder cancer: two meta-analyses. Cancer Epidemiol Biomarkers Prev 18:1312-1320.

van Maanen MJ, Beijnen JH. 1999. Liquid chromatographic-mass spectrometric determination of the novel, recently identified thioTEPA metabolite, thioTEPA-mercapturate, in urine. J Chromatogr B Biomed Sci Appl 732:73-79.

van Maanen MJ, Smits KD, Beijnen JH. 2000. Simultaneous determination of thioTEPA, TEPA and a novel, recently identified thioTEPA metabolite, monochloroTEPA, in urine using capillary gas chromatography. J Chromatogr B Biomed Sci Appl 742:335-343.

Varona ME, Diaz-Criollo SM, Lancheros-Bernal AR, Murcia-Orjuela AM, Henao-Londono GL, Idrovo AJ. 2010. Organochlorine pesticide exposure among agricultural workers in Colombian regions with illegal crops: an exploration in a hidden and dangerous world. Int J Environ Health Res 20:407-414.

Vaughan GT, Kenyon RS. 1996. Monitoring for occupational exposure to 4,4'-methylenebis(2-chloroaniline) by gas chromatographic-mass spectrometric analysis of haemoglobin adducts, blood, plasma and urine. J Chromatogr B, Biomed Appl 678:197-204.

Vesper HW, Caudill SP, Osterloh JD, Meyers T, Scott D, Myers GL. 2010. Exposure of the U.S. population to acrylamide in the National Health and Nutrition Examination Survey 2003-2004. Environ Health Perspect 118:278-283.

Viberg P, Wahlund KG, Skog K. 2006. On-line capillary based quantitative analysis of a heterocyclic amine in human urine. J Chromatogr A 1133:347-352.

Villarini M, Dominici L, Piccinini R, Fatigoni C, Ambrogi M, Curti G, et al. 2011. Assessment of primary, oxidative and excision repaired DNA damage in hospital personnel handling antineoplastic drugs. Mutagenesis 26:359-369.

Vitali M, Ensabella F, Stella D, Guidotti M. 2006. Exposure to organic solvents among handicraft car painters: a pilot study in Italy. Ind Health 44:310-317.

Vodicka P, Koskinen M, Arand M, Oesch F, Hemminki K. 2002a. Spectrum of styrene-induced DNA adducts: the relationship to other biomarkers and prospects in human biomonitoring. Mutat Res 511:239-254.

Vodicka P, Stetina R, Koskinen M, Soucek P, Vodickova L, Hlavac P, et al. 2002b. New aspects in the biomonitoring of occupational exposure to styrene. Int Arch Occup Environ Health 75 Suppl:S75-85.

von Sassen W, Castro-Parra M, Musch E, Eichelbaum M. 1985. Determination of isoniazid, acetylisoniazid, acetylhydrazine and diacetylhydrazine in biological fluids by high-performance liquid chromatography. J Chromatogr 338:113-122.

von Stedingk H, Rydberg P, Törnqvist M. 2010. A new modified Edman procedure for analysis of N-terminal valine adducts in hemoglobin by LC-MS/MS. J Chromatogr B Analyt Technol Biomed Life Sci.

von Stedingk H, Vikstrom AC, Rydberg P, Pedersen M, Nielsen JK, Segerback D, et al. 2011. Analysis of hemoglobin adducts from acrylamide, glycidamide, and ethylene oxide in paired mother/cord blood samples from Denmark. Chem Res Toxicol 24:1957-1965.

Waidyanatha S, Zheng Y, Rappaport SM. 2003. Determination of polycyclic aromatic hydrocarbons in urine of coke oven workers by headspace solid phase microextraction and gas chromatography-mass spectrometry. Chem Biol Interact 145:165-174.

Waksman JC, Phillips SD. 2004. Biologic markers of exposure to chlorinated solvents. Clin Occup Environ Med 4:413-421, v-413-421, v.

Walters DG, Young PJ, Agus C, Knize MG, Boobis AR, Gooderham NJ, et al. 2004. Cruciferous vegetable consumption alters the metabolism of the dietary carcinogen 2-amino-1-methyl-6-phenylimidazo[4,5-b]pyridine (PhIP) in humans. Carcinogenesis 25:1659-1669.

Wang CJ, Shao H, Shang M, Zhang W. 2009. [Association between CYP2B6, CYP2D6, GSTP1 genetic polymorphisms and urinary styrene metabolites in professional workers]. Zhonghua Lao Dong Wei Sheng Zhi Ye Bing Za Zhi 27:589-592.

Wang J, Liang Q, Mendes P, Sarkar M. 2011. Is 24h nicotine equivalents a surrogate for smoke exposure based on its relationship with other biomarkers of exposure? Biomarkers 16:144-154.

Wang X, Vernikovskaya DI, Nanovskaya TN, Rytting E, Hankins GD, Ahmed MS. 2013. A liquid chromatography method with single quadrupole mass spectrometry for quantitative determination of indomethacin in maternal plasma and urine of pregnant patients. J Pharm Biomed Anal 78-79:123-128.

Ward EM, Sabbioni G, DeBord DG, Teass AW, Brown KK, Talaska GG, et al. 1996. Monitoring of aromatic amine exposures in workers at a chemical plant with a known bladder cancer excess. J Natl Cancer Inst 88:1046-1052.

Weisel CP, Kim H, Haltmeier P, Klotz JB. 1999. Exposure estimates to disinfection by-products of chlorinated drinking water. Environ Health Perspect 107:103-110.

Weisel CP. 2010. Benzene exposure: an overview of monitoring methods and their findings. Chem Biol Interact 184:58-66.

Whyatt RM, Barr DB, Camann DE, Kinney PL, Barr JR, Andrews HF, et al. 2003. Contemporary-use pesticides in personal air samples during pregnancy and blood samples at delivery among urban minority mothers and newborns. Environ Health Perspect 111:749-756.

Wu J, Hou H, Ritz B, Chen Y. 2010. Exposure to polycyclic aromatic hydrocarbons and missed abortion in early pregnancy in a Chinese population. Sci Total Environ 408:2312-2318.

Wu X, Zhang G, Wu Y, Hou X, Yuan Z. 2007. Simultaneous determination of malachite green, gentian violet and their leuco-metabolites in aquatic products by high-performance liquid chromatography-linear ion trap mass spectrometry. J Chromatogr A 1172:121-126.

Wu YY, Shi WX, Chen SQ. 2009. [Determination of beta-estradiol, bisphenol A, diethylstilbestrol and salbutamol in human urine by GC/MS]. Zhejiang Da Xue Xue Bao Yi Xue Ban 38:235-241.

Xu J, Jin H, Zhu H, Zheng M, Wang B, Liu C, et al. 2013. Oral bioavailability of rifampicin, isoniazid, ethambutol, and pyrazinamide in a 4-drug fixed-dose combination compared with the separate formulations in healthy chinese male volunteers. Clin Ther 35:161-168.

Xu YP, Liu LQ, Li QS, Peng CF, Chen W, Xu CL. 2009. Development of an immunochromatographic assay for rapid detection of 1-Aminohydantoin in urine specimens. Biomed Chromatogr 23:308-314.

Yardim Y, Keskin E, Levent A, Ozsoz M, Senturk Z. 2010. Voltammetric studies on the potent carcinogen, 7,12-dimethylbenz[a]anthracene: Adsorptive stripping voltammetric determination in bulk aqueous forms and human urine samples and detection of DNA interaction on pencil graphite electrode. Talanta 80:1347-1355.

Yardim Y, Levent A, Ekin S, Keskin E, Oto G, Senturk Z. 2012. Determination of 7,12-Dimethylbenz[a]anthracene in Orally Treated Rats by High-Performance Liquid Chromatography and Transfer Stripping Voltammetry. Comb Chem High Throughput Screen.

Yong LC, Schulte PA, Kao CY, Giese RW, Boeniger MF, Strauss GH, et al. 2007. DNA adducts in granulocytes of hospital workers exposed to ethylene oxide. Am J Ind Med 50:293-302.

Yoxall V, Wilson J, Ioannides C. 2004. An improved method for the extraction of mutagens from human urine and cooked meat using blue rayon. Mutat Res 559:121-130.

Zayas B, Stillwell SW, Wishnok JS, Trudel LJ, Skipper P, Yu MC, et al. 2007. Detection and quantification of 4-ABP adducts in DNA from bladder cancer patients. Carcinogenesis 28:342-349.

Zenzes MT, Bielecki R, Reed TE. 1999. Detection of benzo(a)pyrene diol epoxide-DNA adducts in sperm of men exposed to cigarette smoke. Fertil Steril 72:330-335.

Zhang Z, Wang D, Zhang L, Du M, Chen G. 2008. Determination of diuretics in human urine by hollow fiber-based liquid-liquid-liquid microextraction coupled to high performance liquid chromatography. The Analyst 133:1187-1194.

Zhao C, Vodicka P, Sram RJ, Hemminki K. 2001. DNA adducts of 1,3-butadiene in humans: relationships to exposure, GST genotypes, single-strand breaks, and cytogenetic end points. Environ Mol Mutagen 37:226-230.

Zhao WJ, Liu W, Chen JB, Zhou ZM, Yang MM. 2011. Use of cloud point extraction with derivatizing reagent for the extraction and determination of isoniazid. J Chromatogr Sci 49:154-158.

Zhou P, Wu Y, Yin S, Li J, Zhao Y, Zhang L, et al. 2011. National survey of the levels of persistent organochlorine pesticides in the breast milk of mothers in China. Environ Pollut 159:524-531.

Zhou Z, Chen L, Liu P, Shen M, Zou F. 2010. Simultaneous determination of isoniazid, pyrazinamide, rifampicin and acetylisoniazid in human plasma by high-performance liquid chromatography. Anal Sci 26:1133-1138.

Zhou ZM, Zhao DY, Wang J, Zhao WJ, Yang MM. 2009. Study of cloud point extraction and high-performance liquid chromatographic determination of isoniazid based on the formation of isonicotinylhydrazone. J Chromatogr A 1216:30-35.

Zhu J, Rashid A, Cleary K, Abbruzzese JL, Friess H, Takahashi S, et al. 2006. Detection of 2-amino-1-methyl-6-phenylimidazo [4,5-b]-pyridine (PhIP)-DNA adducts in human pancreatic tissues. Biomarkers 11:319-328.

Zhu K, Wang P, Lin Y, Xiao S, Mei S. 2007. [Simultaneous determination of residues of malachite green, crystal violet and their leuco metabolites in aquatic products by liquid chromatography-tandem mass spectrometry]. Se Pu 25:66-69.

Ziegler E, Mason HJ, Baxter PJ. 2002. Occupational exposure to cytotoxic drugs in two UK oncology wards. Occup Environ Med 59:608-612.

Ziegler RG, Faupel-Badger JM, Sue LY, Fuhrman BJ, Falk RT, Boyd-Morin J, et al. 2010. A new approach to measuring estrogen exposure and metabolism in epidemiologic studies. J Steroid Biochem Mol Biol 121:538-545.

Zou Y, Li Y, Jin H, Tang H, Zou D, Liu M, et al. 2012. Determination of estrogens in human urine by high-performance liquid chromatography/diode array detection with ultrasound-assisted cloud-point extraction. Anal Biochem 421:378-384.

Zwirner-Baier I, Neumann HG. 1999. Polycyclic nitroarenes (nitro-PAHs) as biomarkers of exposure to diesel exhaust. Mutat Res 441:135-144.
